# The role of colour flows in matrix element computations and Monte Carlo simulations

**DOI:** 10.1007/JHEP11(2021)045

**Published:** 2021-11-08

**Authors:** Stefano Frixione, Bryan R. Webber

**Affiliations:** 1 INFN, Sezione di Genova, Via Dodecaneso 33, I-16146 Genoa, Italy; 2 PH Department, TH Division, CERN, CH-1211 Geneva 23, Switzerland; 3 Cavendish Laboratory, University of Cambridge, J.J. Thomson Avenue, Cambridge, CB3 0HE UK

**Keywords:** NLO Computations, QCD Phenomenology

## Abstract

We discuss how colour flows can be used to simplify the computation of matrix elements, and in the context of parton shower Monte Carlos with accuracy beyond leading-colour. We show that, by systematically employing them, the results for tree-level matrix elements and their soft limits can be given in a closed form that does not require any colour algebra. The colour flows that we define are a natural generalization of those exploited by existing Monte Carlos; we construct their representations in terms of different but conceptually equivalent quantities, namely colour loops and dipole graphs, and examine how these objects may help to extend the accuracy of Monte Carlos through the inclusion of subleading-colour effects. We show how the results that we obtain can be used, with trivial modifications, in the context of QCD+QED simulations, since we are able to put the gluon and photon soft-radiation patterns on the same footing. We also comment on some peculiar properties of gluon-only colour flows, and their relationships with established results in the mathematics of permutations.

ArXiv ePrint: 2106.13471

## Contents

**1 Introduction** **1**

**2 Summary of basic concepts** **3**

**3 Colour loops** **7**

**4 Extended colour space, and the flow representation** **10**

**5 Tree-level matrix elements** **11**
- 5.1 Quark-only matrix elements 11
- 5.2 Quark-gluon matrix elements 12
  - 5.2.1 Summing over flows 17
- 5.3 Gluon-only matrix elements 20
  - 5.3.1 Summing over flows 22

**6 Soft limits** **23**
- 6.1 Quark-only matrix elements 24
- 6.2 Quark-gluon matrix elements 25
- 6.3 Gluon-only matrix elements 27
- 6.4 General considerations 28
- 6.5 Examples 29
  - 6.5.1 $\bar{q}q \to gg$ 29
  - 6.5.2 $\bar{q}q \to q'\bar{q}'g$ 31

**7 Monte Carlo simulation** **35**
- 7.1 Single-parton showers 36
  - 7.1.1 Angular-ordered shower 36
- 7.2 Dipole showers 39
  - 7.2.1 Dipole graphs 39
  - 7.2.2 Self-connected gluons 41
- 7.3 Examples 42
  - 7.3.1 $\bar{q}q \to gg$ 42
  - 7.3.2 $\bar{q}q \to q'\bar{q}'g$ 44
- 7.4 Closed colour flows versus dipole graphs 46
- 7.5 Subevents 49

**8 The mixed QCD-QED case** **50**

**9 Conclusions** **54**

JHEP11(2021)045

**A On gluon-only colour flows and colour factors** **57**
A.1 Colour flows 58
A.2 Colour factors 61

**B Equivalence of closed colour flows and dipole graphs** **64**

JHEP11(2021)045

## 1 Introduction

Barring an unlikely turn of events, searches for new physics at the LHC will increasingly rely upon high-precision methods, on both the experimental (with the collection of large-statistics samples and the reduction of systematics) and the theoretical sides. An obvious benefit of high-precision results is that they also allow one to carry out progressively more stringent checks on the (supposedly) well-known Standard Model physics, which in turn help stress-test, and ultimately hone, theory predictions.

From the theoretical viewpoint, meeting the precision targets that current and future experimental data demand almost always requires performing computations that are perturbative in the series expansion in terms of a coupling constant (the so-called fixed-order predictions) at the highest possible accuracy. At the LHC, one is obviously dominated by QCD effects, but electroweak contributions must nowadays be considered as well, especially in the large transverse momentum regions where some characteristic new-physics phenomena are expected to show up. In the past few years, the progress in fixed-order technologies has been extremely significant, for example with next-to-next-to-leading order (NNLO) results becoming routinely available. That being said, fixed-order predictions cannot generally be directly compared to experimental data: even when they are fully differential (a feature that is more difficult to achieve the higher the perturbative accuracy of the computation), hadronization and some acceptance corrections generally need be applied.

It is therefore desirable that such corrections also be carried out at the highest possible accuracy, so as not to spoil that of the underlying fixed-order calculations. This operation more often than not involves the matching and/or merging of fixed-order results with parton-shower Monte Carlo (PSMC) simulations. Unfortunately, the systematic application of matching and merging techniques is currently limited to next-to-leading order (NLO) at most. Furthermore, the PSMCs employed in the context of these techniques are formally of leading-logarithmic (LL) and leading-colour (LC) accuracy, although in practice they perform (in comparison to analytically-resummed computations) better than that.

Another issue that emerges when one considers large-statistics data samples is that many-jet observables become accessible; loosely speaking, this may also be viewed theoretically as a high-precision problem, although of a different nature from those posed by higher-order computations. In particular, here one must be able to calculate the matrix elements[1] associated with a large number of partons in an efficient way. The complexity of this task grows rapidly, and sooner or later one must consider approximated, as opposed to exact, matrix-element results.

The primary aim of this paper is to illustrate how several of the problems outlined thus far can be addressed by using colour flows. This applies to both tree-level matrix element computations, and to the inclusion of beyond-LC effects in PSMCs. We argue that by employing colour flows at both the matrix-element and the PSMC level one has the advantage of a language common to the two cases, which should facilitate the interplay between them in the attempt at increasing the overall precision of the final predictions. Here, we focus mainly on the use of colour flows in tree-level matrix elements to provide a full-colour treatment of the initial conditions for parton showers. The same approach can in principle be applied in the subsequent evolution of the showers. We remark that the extension of PSMCs beyond leading (logarithmic and/or colour) accuracy is the subject of much ongoing activity (see e.g. refs. [1–16]) in directions that are possibly orthogonal to the methods discussed here; a comparison between all these developments is beyond the scope of the present work.

We point out that while the concept of colour flow is well known, our work exploits it at the level of matrix elements (i.e. of amplitudes squared), in contrast to what is typically done in the literature (see e.g. refs. [17–22] and references therein), where the emphasis is at the amplitude level. By doing so, we are naturally led to introduce quantities (such as closed flows and secondary flows) that help to express the matrix elements in a very compact way that does not require any further colour algebra, and is independent of whether the original amplitudes are written in terms of a fundamental or a colour-flow basis. An interesting question that thus emerges is whether similar expressions could be obtained for matrix elements beyond the tree-level (which, at one loop, would be a natural complement of the real-emission subtraction procedure presented in ref. [23]). While the general ideas on colour flows we employ here are certainly valid beyond tree-level, there are specific aspects of the representations of loop amplitudes in terms of colour bases (see e.g. refs. [24–29]) that require an explicit analysis, and whose implications are therefore left to future work.

This paper is organized as follows. In view of the fact that we often rely on the methods of ref. [23], in section 2 we give a brief summary of the results of that paper that are directly relevant here. In section 3, we define the colour loops that stem from a given choice of colour flows. In section 4 we discuss the so-called flow representation of matrix elements in geometrical terms. Sections 5 and 6 present the results for tree-level matrix elements and their soft limits, respectively, expressed in terms of elementary quantities associated with colour flows. In section 7 we show how the results obtained for the matrix elements could be employed in the context of PSMCs that include beyond-LC effects, and we propose two different but equivalent ideas that generalize the concept of colour connections used in existing PSMCs. In section 8 we illustrate how colour flows provide a natural language to deal with the simultaneous perturbative expansion in two parameters,

[1]Note that throughout this paper we use the term "matrix element" to denote the modulus-squared of the sum of amplitudes relevant to the computation of a cross section.

JHEP11(2021)045

using the QCD+QED case as a definite example. Finally, in section 9 we summarize our findings. The appendices collect some technical material. In appendix A miscellaneous results are presented that are relevant to gluon-only colour flows, with the reinterpretation of some of their properties in the context of the mathematics of permutations, whereas appendix B gives some additional information on an idea introduced in section 7 that is particularly relevant to dipole-based showers.

## 2 Summary of basic concepts

The present work makes extensive use of the results of ref. [23]. For the reader who is not familiar with that paper, in this section we give a very brief summary of the ideas that underpin the derivations that will follow later. Henceforth, eq. (x.y) of ref. [23] will be denoted by eq. (**I**.x.y).

All partons[2] that participate in a hard scattering are regarded as outgoing. For a process with $q$ quark-antiquark pairs and $n$ gluons

$$0 \longrightarrow 2q+n \tag{2.1}$$

the labelling conventions for the $i^{th}$ parton are as follows:

$$-2q \le i \le -q-1 \quad\longrightarrow\quad \text{antiquarks}\,, \tag{2.2}$$
$$-q \le i \le -1 \quad\longrightarrow\quad \text{quarks}\,, \tag{2.3}$$
$$1 \le i \le n \quad\longrightarrow\quad \text{gluons}\,. \tag{2.4}$$

Tree-level scattering amplitudes are written as sums of products of dual (or colour-ordered) amplitudes times colour factors. For any given choice of the representation of the SU($N$) colour algebra, colour factors are in one-to-one correspondence with colour flows, which are ordered sets of parton labels, and can be obtained from the latter by means of simple rules. For the process of eq. (2.1), the colour flows have the following form:

$$\gamma = \bigcup_{p=1}^{q} \gamma_p\,, \tag{2.5}$$

$$\gamma_p = \Big(-p\,;\sigma(t_{p-1}+1),\ldots\sigma(t_p);\mu(-p-q)\Big)\,. \tag{2.6}$$

Here, $\sigma$ and $\mu$ denote permutations (including cyclic ones) of the first $n$ and $q$ integers,[3] respectively, while the set of $q+1$ integers $t_p$

$$t = \{t_0,\ldots t_q\}\ , \tag{2.7}$$
$$0 = t_0 \le t_1 \le \ldots t_{q-1} \le t_q = n\,, \tag{2.8}$$

is a partition of the ordered set of the first $n$ integers into $q$ subsets of ordered integers, with the $p^{th}$ cell of the partition defined to be[4]

$$(t_{p-1}+1, t_{p-1}+2, \ldots t_p-1, t_p)\ , \qquad 1 \le p \le q\,. \tag{2.9}$$

[2] Assumed to be massless; the generalization to massive partons can be made along similar lines.

[3] In eq. (2.6) and elsewhere, $\mu(-p-q)$ is symbolic for $-\mu(p)-q$.

[4] Equation (2.9) applies only to the non-trivial case $t_{p-1} < t_p$; when $t_{p-1} = t_p$, the $p^{th}$ cell of the partition is $(t_{p-1}, t_p)$. See also footnote 5.

JHEP11(2021)045

The set of $m$-integer permutations is denoted by $P_m$; the set of the partitions of eqs. (2.7) and (2.8) is denoted by $T_{n|q}$. Thus, the set of colour flows relevant to the process of eq. (2.1) is given by eq. (**I**.3.27)

$$\mathcal{F}_{2q;n} = \Big(P_n, P_q, T_{n|q}\Big) \; , \tag{2.10}$$

whose number of elements is equal to

$$|\mathcal{F}_{2q;n}| = |P_n|\,|P_q|\,\Big|T_{n|q}\Big| = n!\,q! \begin{pmatrix} n+q-1 \\ q-1 \end{pmatrix} = q\,(n+q-1)!\,. \tag{2.11}$$

Each of the ordered sets in $\gamma_p$ in eq. (2.5) is called an open colour line, that connects quark $-p$ with antiquark $\mu(-p-q)$ and features the emission of gluon $\sigma(t_{p-1}+1)$ (which is the closest to the quark) up to gluon $\sigma(t_p)$ (which is the closest to the antiquark). Denoting by

$$\{a_i\}_{i=1}^{n} \; , \qquad a_i \in \Big\{1, \ldots N^2-1\Big\} \; , \tag{2.12}$$

$$\{a_i\}_{i=-2q}^{-1} \; , \qquad a_i \in \{1, \ldots N\} \; , \tag{2.13}$$

the colours of gluons, and of quarks and antiquarks, respectively, the colour flow of eq. (2.5) is associated with the following colour factor in the fundamental representation[5]

$$\begin{aligned}\Lambda\left(\{a_i\},\gamma\right) = N^{-\rho(\gamma)} &\left(\lambda^{a_{\sigma(t_0+1)}} \ldots \lambda^{a_{\sigma(t_1)}}\right)_{a_{-1}a_{\mu(-1-q)}} \\ &\times \left(\lambda^{a_{\sigma(t_1+1)}} \ldots \lambda^{a_{\sigma(t_2)}}\right)_{a_{-2}a_{\mu(-2-q)}} \\ &\times \ldots \\ &\times \left(\lambda^{a_{\sigma(t_{q-1}+1)}} \ldots \lambda^{a_{\sigma(t_q)}}\right)_{a_{-q}a_{\mu(-2q)}} \; ,\end{aligned} \tag{2.14}$$

where

$$\rho\left(\gamma\right) = \min\left\{ q-1, \, \sum_{p=1}^{q} \delta\Big(-p-q, \mu(-p-q)\Big) \right\} \, . \tag{2.15}$$

The integer $\rho(\gamma)$ is equal to the number of open colour lines that coincide with flavour lines,[6] minus one in the case of maximal coincidence. Given a colour configuration, i.e. a specific choice for the colours of quarks, antiquarks, and gluons, the scattering amplitude is written as was anticipated, i.e.

$$\mathcal{A}^{(2q;n)}(a_{-2q}, \ldots a_n) = \sum_{\gamma \in \mathcal{F}_{2q;n}} \Lambda\left(\{a_i\},\gamma\right) \widehat{\mathcal{A}}^{(2q;n)}\left(\gamma\right) \; , \tag{2.16}$$

where $\widehat{\mathcal{A}}^{(2q;n)}(\gamma)$ is the dual amplitude associated with the colour flow $\gamma$. In ref. [23] it has been found convenient to represent amplitudes in a vector space, called colour space, and

[5] If an open colour line does not feature any gluons, the corresponding colour factor is a Kronecker $\delta$ — see eq. (**I**.3.23) and eq. (**I**.3.24). Furthermore, throughout this paper $\lambda$ matrices are normalized as the SU(3) generators, per appendix A of ref. [23]..

[6] In the unequal-flavour case; see eq. (**I**.3.30)–eq. (**I**.3.32) when there are at least two equal flavour pairs.

JHEP11(2021)045

denoted by $S_{\{a\}}$. The set of all colour configurations corresponds to a complete basis of orthonormal vectors in $S_{\{a\}}$, thus[7]

$$|a_{-2q}, \dots a_n\rangle \in S_{\{a\}}\,, \tag{2.17}$$

$$\langle b_{-2q}, \dots b_n | a_{-2q}, \dots a_n\rangle = \prod_{i=-2q}^{n} \delta_{a_i b_i}\,, \tag{2.18}$$

$$\sum_{\{a_i\}_{i=-2q}^{n}} |a_{-2q}, \dots a_n\rangle\langle a_{-2q}, \dots a_n| = I\,. \tag{2.19}$$

The colour space is separable into the subspaces $S^{(p)}_{\{a\}}$ relevant to each parton $p$ that enters the hard process

$$S_{\{a\}} = \bigotimes_{p=-2q}^{n} S^{(p)}_{\{a\}}\,, \tag{2.20}$$

$$|a_{-2q}, \dots a_n\rangle = \bigotimes_{p=-2q}^{n} |a_p\rangle\,, \qquad |a_p\rangle \in S^{(p)}_{\{a\}}\,. \tag{2.21}$$

The dimensions of these vector spaces are

$$\left|S^{(p)}_{\{a\}}\right| = \begin{cases} N & -2q \le p \le -1 \\ N^2 - 1 & 1 \le p \le n \end{cases} \quad \Longrightarrow \quad \left|S_{\{a\}}\right| = N^{2q}\left(N^2 - 1\right)^n\,. \tag{2.22}$$

With this, one can introduce vectors associated with amplitudes at given colour configurations or colour flows, thus

$$|\mathcal{A}^{(2q;n)}(a_{-2q}, \dots a_n)\rangle = \mathcal{A}^{(2q;n)}(a_{-2q}, \dots a_n)\,|a_{-2q}, \dots a_n\rangle\,, \tag{2.23}$$

$$|\mathcal{A}^{(2q;n)}(\gamma)\rangle = \sum_{\{a_i\}_{i=-2q}^{n}} \Lambda\left(\{a_i\}, \gamma\right)\, \widehat{\mathcal{A}}^{(2q;n)}(\gamma)\,|a_{-2q}, \dots a_n\rangle\,, \tag{2.24}$$

from which one can obtain the colour-summed amplitude

$$|\mathcal{A}^{(2q;n)}\rangle = \sum_{\{a_i\}_{i=-2q}^{n}} |\mathcal{A}^{(2q;n)}(a_{-2q}, \dots a_n)\rangle \tag{2.25}$$

$$= \sum_{\gamma \in \mathcal{F}_{2q;n}} |\mathcal{A}^{(2q;n)}(\gamma)\rangle\,. \tag{2.26}$$

By interfering a colour-summed amplitude with its complex conjugate one obtains the matrix element that, multiplied by flux, average, and phase-space factors gives the physical cross section

$$\mathcal{M}^{(2q;n)} \equiv \langle \mathcal{A}^{(2q;n)} | \mathcal{A}^{(2q;n)}\rangle = \sum_{\gamma', \gamma \in \mathcal{F}_{2q;n}} \mathcal{M}^{(2q;n)}\left(\gamma', \gamma\right)\,, \tag{2.27}$$

with

$$\mathcal{M}^{(2q;n)}\left(\gamma', \gamma\right) = \langle \mathcal{A}^{(2q;n)}\left(\gamma'\right) | \mathcal{A}^{(2q;n)}\left(\gamma\right)\rangle \tag{2.28}$$

$$= \widehat{\mathcal{A}}^{(2q;n)}\left(\gamma'\right)^{\star}\, C\left(\gamma', \gamma\right)\, \widehat{\mathcal{A}}^{(2q;n)}\left(\gamma\right)\,. \tag{2.29}$$

[7] Throughout this paper, summation and product indices may have ranges that include zero; we understand the corresponding contributions to be null.

JHEP11(2021)045

The r.h.s. of eq. (2.29) is expressed in terms of scalar quantities, namely the dual amplitudes already introduced, and the colour-flow matrix element

$$C\left(\gamma',\gamma\right)=\sum_{\{a_i\}_{i=-2q}^{n}}\Lambda\left(\{a_i\},\gamma'\right)^{\star}\Lambda\left(\{a_i\},\gamma\right)\,. \tag{2.30}$$

Equations (2.27)–(2.30) give us the opportunity to stress a fact left implicit so far. Namely, a colour flow is a quantity defined at the amplitude level. In the computation of cross sections, what is relevant is always a pair of two flows, one on each side of the cut. Such a pair, i.e.

$$(\gamma',\gamma) \tag{2.31}$$

in the expressions above, is called a *closed colour flow*. In eq. (2.31), it is understood that $\gamma'$ $(\gamma)$ is the flow entering the amplitude on the left-hand side (right-hand side) of the cut. Because of this, when it is necessary to distinguish between the colour flows on either side of the cut they will be called L-flows and R-flows.

The formulae written so far are relevant to the process of eq. (2.1). Although in principle that could be used to describe purely gluonic processes as well (i.e. when $q=0$), in practice such a case entails significant simplifications that justify the use of a slightly different notation. In particular, for a purely-gluonic $n$-body process

$$0\;\longrightarrow\;n\,, \tag{2.32}$$

the analogue of the colour flow of eq. (2.5) features a single colour line, denoted as follows:

$$\sigma\equiv\left(\sigma(1),\ldots\sigma(n)\right)\,. \tag{2.33}$$

Furthermore, in view of the fact that its associated colour factor, i.e. the analogue of eq. (2.14), reads in the fundamental representation

$$\Lambda\left(\{a_i\},\sigma\right)=\mathrm{Tr}\Big(\lambda^{a_{\sigma(1)}}\ldots\lambda^{a_{\sigma(n)}}\Big)\,, \tag{2.34}$$

the information embedded in the colour flow must be invariant under cyclic permutations. Therefore, the set of the $n$-gluon colour flows is the set of the non-cyclic $n$-integer permutations, which we shall denote by

$$P_n'\,, \tag{2.35}$$

and which constitutes the analogue of the set introduced in eq. (2.10). These, and the formal replacements

$$\mathcal{A}^{(2q;n)}\longrightarrow\mathcal{A}^{(n)}\,, \tag{2.36}$$

$$\mathcal{M}^{(2q;n)}\longrightarrow\mathcal{M}^{(n)}\,, \tag{2.37}$$

are all that is needed in order to convert the expressions for the quark-gluon matrix elements and related quantities into those relevant to the gluon-only case.

JHEP11(2021)045

## 3 Colour loops

In the context of current parton shower Monte Carlos, colour flows stemming from matrix element computations are responsible for giving crucial information on the initial conditions for the showers (and for hadronization); therefore, ultimately they have a major role in the determination of the radiation pattern.

In retrospect, this might appear strange for two reasons. Firstly, as was discussed in section 2, the quantities that are relevant to matrix elements are not flows, but closed flows. Secondly, such flows are Born-level (i.e. $m$-body) objects, while even the first-emission radiation pattern is determined, in cross section computations, by $(m+1)$-body objects. As far as the first item is concerned, the key point is that existing PSMCs only consider closed flows for which the L-flow coincides with the R-flow; in other words, only the diagonal of the colour-flow matrix is taken into account, which is consistent with the leading-$N$ nature of the MCs. Obviously, then, the information on the closed flow is redundant, and that of the flow is used instead. In turn, this immediately suggests that if one is interested in going beyond the leading-$N$ approximation in MCs, retaining the information on closed flows (e.g. in determining colour connections) will be essential. As far as the second observation above is concerned, this has again to do with the leading-$N$ character of PSMCs, which is such that the radiation pattern at the $(m+1)$-body level can be described by $m$-body quantities. Beyond the leading-$N$ this is not true any longer. However, we shall show how one can circumvent this difficulty, and thus to keep on working with $m$-body objects.

Before proceeding, we need to define precisely colour loops, which will be used throughout this paper. Firstly, colour loops are properties of a given closed colour flow. The set of all colour loops relevant to the closed flow $(\gamma', \gamma)$ will be denoted by

$$\mathcal{L}(\gamma', \gamma)\,. \tag{3.1}$$

Each loop is defined iteratively, as follows:

**0.** Write the L- and R-flow as two vertical sets of indices (the former to the left of the latter). Each open colour line that belongs to these flows is a separate subset of such sets. The relative order of these subsets is irrelevant.

**1.** Start from either a quark or a gluon that belongs to the R-flow; this is the first element of the loop.

**2.** Go down one place in the R-flow; a gluon or an antiquark is found.

**3.** Jump to the L-flow, landing on the element whose label is identical to that of the gluon or the antiquark of the previous step.

**4.** Go up one place in the L-flow; a gluon or a quark is found.

**5.** Jump to the R-flow, landing on the element whose label is identical to that of the gluon or the quark of the previous step.

JHEP11(2021)045

6. When the landing element coincides with the first element of the loop then go to step **7**, otherwise iterate steps **2**–**5**.

7. A loop is the ordered set composed of the starting element and of all of the landing elements subsequently found, except for the last one (because it coincides with the starting element).

Once a loop has been constructed, the next one (if it exists) can be found by following again steps 1–7, with the condition that the starting element in step 1 must not be already included as an R-element of one of the loops previously constructed. We call L- and R-elements of a given loop $\ell \in \mathcal{L}$ the landing elements when jumping from right to left and from left to right, respectively; on top of those, the starting element of the loop is included in the set of R-elements as well.

By construction, colour loops have the following properties:

**i.** The sets of R-elements of any two loops are disjoint (i.e. have an empty intersection); the same is true for the L-elements.

**ii.** All quarks are R-elements, and all antiquarks are L-elements.

**iii.** A quark label will appear only once, i.e. it belongs to a single loop; the same is true for antiquarks.

**iv.** A gluon label will appear twice, either in the same loop or in two different loops. In the former case, one instance corresponds to an R-element, and the other to an L-element (i.e. the gluon label cannot correspond to two L- or two R-elements).

These properties will turn out to be important in deriving results for the soft limit of matrix elements. We point out that the idea that underpins the construction of colour loops as given above is based on the usual procedure of following colour (as opposed to anticolour) indices.[8] This is straightforward in the case of quarks and antiquarks; for gluons, one needs to consider the pair of $\lambda$ matrices associated with any gluon index, and to replace them with the first term of the Fierz identity. Roughly speaking, this corresponds to "splitting" colour lines by replacing each gluon there with a $q\bar{q}$ pair (of a fictitious flavour, different from all of the other flavours); this idea will play an important role starting from section 5.2.

We can also introduce the idea of distance within a colour loop. More precisely, for any two parton labels $k$ and $l$ that belong to a loop $\ell$

$$k\,,l \,\in\, \ell \,\in\, \mathcal{L}(\gamma',\gamma)\,, \tag{3.2}$$

the distance between $k$ and $l$, denoted by

$$\delta_\ell(k,l)\,, \tag{3.3}$$

is defined as the number of partons in $\ell$ between $k$ and $l$; thus, if $k$ and $l$ are contiguous, the distance is equal to zero. Furthermore, according to item ii. above and taking into account

[8]Needless to say, this is a purely conventional choice: one might choose the anticolour instead, and the procedure would be unchanged except for the formal replacements left vs right and up vs down.

JHEP11(2021)045

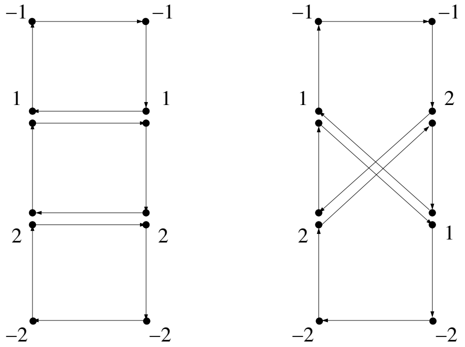

**Figure 1**. Colour loop sets for $0 \to q\bar{q}gg$: $\mathcal{L}(\gamma_1, \gamma_1)$ (left) and $\mathcal{L}(\gamma_1, \gamma_2)$ (right).

that colour loops alternate L- and R-elements, the distance between any quark and any antiquark is an even number, whereas the distance between two quarks or two antiquarks is an odd number.

• *Example.* Consider the case of $0 \to q\bar{q}gg$. According to the all-outgoing labeling of section 2, this process is $0 \to q_{-1}\bar{q}_{-2}g_1g_2$ (i.e. $\bar{q}_{-1}q_{-2} \to g_1g_2$ in a physical configuration), with $q = 1$ and $n = 2$. The set of flows, eq. (2.10), is

$$\mathcal{F}_{2;2} = \Big\{ \big( -1; 1, 2; -2 \big), \big( -1; 2, 1; -2 \big) \Big\} \equiv \Big\{ \gamma_1, \gamma_2 \Big\} . \tag{3.4}$$

The colour loops corresponding to $\mathcal{L}(\gamma_1, \gamma_1)$ and $\mathcal{L}(\gamma_1, \gamma_2)$ are shown in figure 1. Clearly the diagram for $\mathcal{L}(\gamma_2, \gamma_2)$ is that for $\mathcal{L}(\gamma_1, \gamma_1)$ with gluons 1 and 2 interchanged. Thus the sets of colour loops, eq. (3.1), associated with these flows are

$$\mathcal{L}(\gamma_1, \gamma_1) = \Big\{ \big( -1, 1 \big), \big( 1, 2 \big), \big( 2, -2 \big) \Big\} \quad \Longrightarrow \quad |\mathcal{L}(\gamma_1, \gamma_1)| = 3 \, , \tag{3.5}$$

$$\mathcal{L}(\gamma_1, \gamma_2) = \Big\{ \big( -1, 2, 1, -2, 2, 1 \big) \Big\} \quad \Longrightarrow \quad |\mathcal{L}(\gamma_1, \gamma_2)| = 1 \, , \tag{3.6}$$

$$\mathcal{L}(\gamma_2, \gamma_2) = \Big\{ \big( -1, 2 \big), \big( 2, 1 \big), \big( 1, -2 \big) \Big\} \quad \Longrightarrow \quad |\mathcal{L}(\gamma_2, \gamma_2)| = 3 \, . \tag{3.7}$$

For example, with

$$\ell = \Big\{ \big( -1, 2, 1, -2, 2, 1 \big) \Big\} \tag{3.8}$$

we have

$$\delta_\ell(-1, 2) = 0 \, , \quad \delta_\ell(-1, 1) = 1 \, , \quad \delta_\ell(-1, -2) = 2 \, , \quad \delta_\ell(-1, 2) = 3 \, , \quad \delta_\ell(-1, 1) = 4 \, , \tag{3.9}$$

$$\delta_\ell(2, 1) = 0 \, , \quad \delta_\ell(2, -2) = 1 \, , \quad \delta_\ell(2, 2) = 2 \, , \quad \delta_\ell(2, 1) = 3 \, , \tag{3.10}$$

$$\delta_\ell(1, -2) = 0 \, , \quad \delta_\ell(1, 2) = 1 \, , \quad \delta_\ell(1, 1) = 2 \, , \tag{3.11}$$

$$\delta_\ell(-2, 2) = 0 \, , \quad \delta_\ell(-2, 1) = 1 \, , \tag{3.12}$$

$$\delta_\ell(2, 1) = 0 \, . \tag{3.13}$$

Consistently with the notation used throughout this paper for the number of elements of a set (see e.g. eq. (2.11)), in eqs. (3.5)–(3.7) we have denoted by

$$|\mathcal{L}(\gamma', \gamma)| \tag{3.14}$$

JHEP11(2021)045

the number of colour loops of the closed flow $(\gamma', \gamma)$; this quantity will appear throughout this paper.

The construction of colour loops works also in the case of gluon-only amplitudes. Given a closed flow $(\sigma', \sigma)$, steps 0–7 previously described are unchanged (with "quark" or "antiquark" there to be ignored), except for the necessity of including a cyclicity condition, which is simply achieved in the following way:

- Going down one place from the last element in the R-flow means arriving at the first element of that flow.
- Going up one place from the first element in the L-flow means arriving at the last element of that flow.

## 4 Extended colour space, and the flow representation

The colour-anticolour structure of gluons suggests the possibility of working in a vector space larger than that introduced in eq. (2.20). Let $p$ be any gluon; we consider an $N^2$-dimensional vector space $S^{(p)}_{\{ij\}}$ which embeds $S^{(p)}_{\{a\}}$

$$S^{(p)}_{\{a\}} \subset S^{(p)}_{\{ij\}}\,, \qquad \left|S^{(p)}_{\{ij\}}\right| = N^2\,. \tag{4.1}$$

The relationship between $S_{\{a\}}$ and $S_{\{ij\}}$ is established as follows. Let

$$\left\{|ij\rangle\right\}_{i,j=1}^{N} \subset S^{(p)}_{\{ij\}} \tag{4.2}$$

be an orthonormal basis of $S^{(p)}_{\{ij\}}$, so that

$$\langle kl|ij\rangle = \delta_{ik}\delta_{jl}\,, \qquad \sum_{ij}|ij\rangle\langle ij| = I\,. \tag{4.3}$$

The vectors $|a\rangle$ that constitute an orthonormal basis of $S^{(p)}_{\{a\}}$ are then

$$|a\rangle = T_F^{-1/2}\sum_{ij}\lambda^a_{ij}|ij\rangle\,. \tag{4.4}$$

Therefore (see footnote 5 for the normalization)

$$\langle b|a\rangle = T_F^{-1}\sum_{ijkl}\lambda^b_{lk}\lambda^a_{ij}\langle kl|ij\rangle = T_F^{-1}\,\mathrm{Tr}\left(\lambda^b\lambda^a\right) = \delta^{ab}\,, \tag{4.5}$$

which shows that eq. (4.4) behaves as expected. Furthermore

$$\begin{aligned}\mathcal{P}^{(p)}_{\parallel} \equiv \sum_a |a\rangle\langle a| &= T_F^{-1}\sum_a\sum_{ijkl}\lambda^a_{lk}\lambda^a_{ij}|kl\rangle\langle ij| \\ &= \sum_{ijkl}\left(\delta_{ik}\delta_{jl} - \frac{1}{N}\delta_{ij}\delta_{kl}\right)|kl\rangle\langle ij|\,. \end{aligned}\tag{4.6}$$

JHEP11(2021)045

Equation (4.6) suggests defining the vector

$$|\perp\rangle = \frac{1}{\sqrt{N}}\sum_i |ii\rangle \quad\Longrightarrow\quad \langle\perp|\perp\rangle = 1\,, \tag{4.7}$$

so that, given eq. (4.3), from eq. (4.6) one obtains

$$\mathcal{P}_{\parallel}^{(p)} = I - \mathcal{P}_{\perp}^{(p)}\,, \tag{4.8}$$

$$\mathcal{P}_{\perp}^{(p)} = |\perp\rangle\langle\perp|\,. \tag{4.9}$$

It also follows that

$${\mathcal{P}_{\perp}^{(p)}}^2 = \mathcal{P}_{\perp}^{(p)}\,, \qquad {\mathcal{P}_{\parallel}^{(p)}}^2 = \mathcal{P}_{\parallel}^{(p)}\,, \qquad \mathcal{P}_{\parallel}^{(p)}\mathcal{P}_{\perp}^{(p)} = \mathcal{P}_{\perp}^{(p)}\mathcal{P}_{\parallel}^{(p)} = 0\,. \tag{4.10}$$

Therefore, $\mathcal{P}_{\parallel}^{(p)}$ and $\mathcal{P}_{\perp}^{(p)}$ are projectors onto the (physical) $S_{\{a\}}^{(p)}$ and (unphysical) $S_{\{ij\}}^{(p)} \setminus S_{\{a\}}^{(p)}$ subspaces of $S_{\{ij\}}^{(p)}$, respectively. We stress that the definition of $\mathcal{P}_{\parallel}^{(p)}$ in eq. (4.6) is usually an expression for the identity (see eq. (2.19)). This is correct: by construction, $\mathcal{P}_{\parallel}^{(p)}$ *is* the identity in the subspace $S_{\{a\}}^{(p)}$, but not in the larger space $S_{\{ij\}}^{(p)}$. For future use, we note that by direct computation one obtains

$$\mathcal{P}_{\perp}^{(p)}|ij\rangle = \frac{\delta_{ij}}{\sqrt{N}}|\perp\rangle \quad\Longrightarrow\quad \sum_{ij}\mathcal{P}_{\perp}^{(p)}|ij\rangle = \sqrt{N}|\perp\rangle = \sum_{ij}\delta_{ij}|ij\rangle\,. \tag{4.11}$$

In sections 5.2 and 5.3 we shall show how the use of $S_{\{ij\}}^{(p)}$ is in one-to-one correspondence with what is usually called the flow representation for amplitudes, which in the present context will acquire a natural geometrical meaning.

## 5 Tree-level matrix elements

In this section, we present results for tree-level matrix elements, regarded as quantities where all partons are hard and well separated from each other.

### 5.1 Quark-only matrix elements

The processes of interest are those of eq. (2.1), with $n = 0$ there. Because of this, the colour factors are simply products of Kronecker $\delta$'s (see footnote 5) which, when employed in the computation of the colour-flow matrix element of eq. (2.30), are contracted with each other, alternating one Kronecker $\delta$ from the R-flow with one Kronecker $\delta$ from the L-flow. Eventually, one finds a $\delta$ whose right index is equal to the left index of the $\delta$ from which the contraction had started, thus resulting in the trace of the identity matrix, equal to $N$. It is easy to convince oneself that the indices encountered when contracting the $\delta$'s are nothing but the colour labels of the partons that enter the colour loop to which any of these partons belong (bear in mind that any quark or antiquark appears in a single loop). Therefore, each loop is responsible for a contribution equal to $N$ to the colour-flow matrix element which implies, from eqs. (2.14) and (2.29)

$$\mathcal{M}^{(2q;0)}\left(\gamma',\gamma\right) = \widehat{\mathcal{A}}^{(2q;0)}\left(\gamma'\right)^{\star}\,\widehat{\mathcal{A}}^{(2q;0)}\left(\gamma\right)\,N^{-\rho(\gamma')-\rho(\gamma)}\,N^{|\mathcal{L}(\gamma',\gamma)|}\,, \tag{5.1}$$

JHEP11(2021)045

where the quantity $|\mathcal{L}(\gamma',\gamma)|$ has been introduced in eq. (3.14). Summing eq. (5.1) over flows is trivial: by using eq. (2.27) we obtain

$$\mathcal{M}^{(2q;0)} = \sum_{\gamma',\gamma\in\mathcal{F}_{2q;0}} \widehat{\mathcal{A}}^{(2q;0)}(\gamma')^\star\, \widehat{\mathcal{A}}^{(2q;0)}(\gamma)\, N^{-\rho(\gamma')-\rho(\gamma)}\, N^{|\mathcal{L}(\gamma',\gamma)|}\,. \tag{5.2}$$

### 5.2 Quark-gluon matrix elements

The remarkable simplicity of eq. (5.1) stems from the trivial nature of the colour factor relevant to quark-only processes. The presence of gluons complicates the picture; however, we shall show in this section that results for quark-gluon matrix elements can essentially be cast in the same form as eq. (5.1), by introducing quantities that we shall call secondary colour flows, and that can be derived from the given colour flows.

The starting point is the vector $|\mathcal{A}^{(2q;n)}(\gamma)\rangle \in S_{\{a\}}$ that represents the amplitude for a given flow $\gamma$, eq. (2.24). Consider one of the gluons that enter the process; it is not restrictive to assume its label to be 1. There exists a quark with label $-p_1$ such that

$$\gamma = \Big(-p_1;\ldots,1,\ldots;\mu(-p_1-q)\Big)\,\hat\gamma\,. \tag{5.3}$$

In other words, gluon 1 belongs to the colour line that begins at quark $-p_1$ and ends at antiquark $\mu(-p_1-q)$. Write the colour factor associated with $\gamma$ as follows:

$$\Lambda(\gamma) = N^{-\rho(\gamma)}\left(\Lambda^{(A)}\lambda^{a_1}\Lambda^{(B)}\right)_{a_{-p_1}a_{\mu(-p_1-q)}}\hat\Lambda(\hat\gamma)\,, \tag{5.4}$$

with $\Lambda^{(A)}$ and $\Lambda^{(B)}$ suitable products of $\lambda$ matrices; the definition of $\hat\Lambda(\hat\gamma)$ can be derived from eq. (2.14), but will not be needed here. Equation (2.24) can then be re-written more compactly as follows:

$$\begin{aligned}|\mathcal{A}^{(2q;n)}(\gamma)\rangle &= \sum_{\{a_i\}_{i=-2q}^n}\left(\Lambda^{(A)}\lambda^{a_1}\Lambda^{(B)}\right)_{a_{-p_1}a_{\mu(-p_1-q)}}|a_1\rangle\otimes|v\rangle \\ &= T_F^{-1/2}\sum_{ij}\sum_{\{a_i\}_{i=-2q}^n}\left(\Lambda^{(A)}\lambda^{a_1}\Lambda^{(B)}\right)_{a_{-p_1}a_{\mu(-p_1-q)}}\lambda^{a_1}_{ij}|ij\rangle\otimes|v\rangle\,,\end{aligned} \tag{5.5}$$

where

$$|v\rangle = N^{-\rho(\gamma)}\,\hat\Lambda(\hat\gamma)\,\widehat{\mathcal{A}}^{(2q;n)}(\gamma)\bigotimes_{p\neq 1}|a_p\rangle\,, \tag{5.6}$$

and we have used eq. (4.4), understanding $|ij\rangle \in S^{(1)}_{\{ij\}}$. By means of the Fierz identity one obtains

$$\begin{aligned}&T_F^{-1/2}\sum_{ij}\sum_{a_1}\left(\Lambda^{(A)}\lambda^{a_1}\Lambda^{(B)}\right)_{a_{-p_1}a_{\mu(-p_1-q)}}\lambda^{a_1}_{ij}|ij\rangle \\ &= 2^{-1/2}\sum_{ij}\left(\Lambda^{(A)}_{a_{-p_1}j}\Lambda^{(B)}_{ia_{\mu(-p_1-q)}} - \frac{1}{N}\left(\Lambda^{(A)}\Lambda^{(B)}\right)_{a_{-p_1}a_{\mu(-p_1-q)}}\delta_{ij}\right)|ij\rangle \\ &= 2^{-1/2}\sum_{ij}\left(\Lambda^{(A)}_{a_{-p_1}j}\Lambda^{(B)}_{ia_{\mu(-p_1-q)}} - \frac{1}{N}\left(\Lambda^{(A)}\Lambda^{(B)}\right)_{a_{-p_1}a_{\mu(-p_1-q)}}\mathcal{P}^{(1)}_\perp\right)|ij\rangle\,,\end{aligned} \tag{5.7}$$

having used eq. (4.11).

JHEP11(2021)045

The vector on the l.h.s. of eq. (5.7) belongs to $S^{(1)}_{\{a\}}$. It is written in the r.h.s. as the difference of two vectors, the second of which belongs to $S^{(1)}_{\{ij\}} \setminus S^{(1)}_{\{a\}}$. In other words, a physical vector is written as the difference of two unphysical vectors, constructed so that their unphysical components cancel each other. The colour factors associated with these two vectors are also interesting. In that of the first term on the rightmost side of eq. (5.7) the gluon is replaced by a $Q\bar{Q}$ pair with colour and anticolour indices equal to $i$ and $j$, respectively, whereas in that of the second term the gluon disappears altogether. Thus, these colour factors are those one would obtain by employing eq. (2.14) with flows[9]

$$\bar{\gamma}_a = \Big( -p_1; \ldots, \bar{Q} \Big) \Big( Q, \ldots ; \mu(-p_1 - q) \Big)\, \hat{\gamma}\,, \tag{5.8}$$

$$\bar{\gamma}_b = \Big( -p_1; \ldots, \not{1}, \ldots ; \mu(-p_1 - q) \Big)\, \hat{\gamma}\,, \tag{5.9}$$

potentially up to an overall power of $N$, which can however be easily fixed. Namely, the original colour flow $\gamma$ has a pre-factor equal to $N^{-\rho(\gamma)}$, which here is part of the definition of $|v\rangle$ in eq. (5.6). For $\rho(\gamma)$ to be equal to $\rho(\bar{\gamma}_a)$, it is sufficient that the flavour of the fictitious pair $Q\bar{Q}$ be different from all of the other quark flavours relevant to the current process, which is a condition that we can impose by construction. Conversely, since the flavour structure of $\bar{\gamma}_b$ is identical to that of $\gamma$, then $\rho(\gamma) = \rho(\bar{\gamma}_b)$. This however does not take into account the factor $-1/N$ that appears in eq. (5.7); indeed, it will turn out to be convenient (in particular, in view of its negative sign) to account for that factor separately from $\rho(\gamma)$.

The procedure that leads one from eq. (5.5) to eq. (5.7) can be iterated, by considering all of the other gluons one after another. At each step, the number of contributions will grow by a factor of two (two being the number of terms on the r.h.s. of the Fierz identity), with each gluon either replaced by a fictitious quark-antiquark pair, or eliminated; with $n$ gluons, the overall number of contributions will be thus equal to $2^n$. In order to enumerate them, and to construct the analogues of the flows in eqs. (5.8) and (5.9), we introduce the following notation. Let

$$S^{(n)}_k \tag{5.10}$$

be the set of all unordered subsets of $\{1, \ldots n\}$ of length $k$. In other words

$$s_k \in S^{(n)}_k \quad \Longrightarrow \quad s_k = \{j_1, \ldots j_k\} \subseteq \{1, \ldots n\}\,. \tag{5.11}$$

Note that the definition of $S^{(n)}_k$ includes the special cases

$$S^{(n)}_0 = \Big\{ \{\} \Big\}\,, \qquad S^{(n)}_n = \Big\{ \{1, \ldots n\} \Big\}\,. \tag{5.12}$$

Note also that

$$\Big| S^{(n)}_k \Big| = \begin{pmatrix} n \\ k \end{pmatrix}\,, \tag{5.13}$$

[9]For the sake of clarity, with abuse of notation in eq. (5.8) we have used the same symbol $Q$ for a quark and an antiquark, and their labels. This is temporary, and a more precise notation will be adopted soon.

JHEP11(2021)045

and thus that

$$\sum_{k=0}^{n} \left| S_k^{(n)} \right| = 2^n \,. \tag{5.14}$$

The set $S_k^{(n)}$ encompasses all possible choices of $k$ gluon labels out of those of the given $n$ gluons; an element $s_k \in S_k^{(n)}$ is one specific such choice. Then, for a given colour flow $\gamma$ consider the $2^n$-element set

$$\bigcup_{k=0}^{n} \bigcup_{s_k \in S_k^{(n)}} \left\{ \bar{\gamma}_{\not{s}_k} \right\} . \tag{5.15}$$

Each $\bar{\gamma}_{\not{s}_k}$ element is called a *secondary colour flow*, and is constructed by taking $\gamma$ and removing from it the $k$ gluons associated with the labels in $s_k$. As far as the other $n-k$ gluons are concerned, eq. (5.8) tells us that they need be replaced by fictitious quark-antiquark pairs. Since such pairs are essentially a bookkeeping device, it is easier to continue using the same gluon labels, with a bar on top. For future reference, we point out that according to the procedure of section 3 exactly the same colour loops will emerge from closed secondary flows regardless of whether quark-antiquark labels, or barred gluon labels, are employed.

It is a matter of simple algebra to see that the iteration of eq. (5.7) leads to the following result:

$$\begin{aligned} \left|\mathcal{A}^{(2q;n)}(\gamma)\right\rangle = \widehat{\mathcal{A}}^{(2q;n)}(\gamma) \sum_{k=0}^{n} \sum_{s_k \in S_k^{(n)}} \frac{(-1)^k}{2^{n/2} N^k} \sum_{\{a_i\}_{i=-2q}^{-1}} \sum_{\{i_p, j_p\}_{p=1}^{n}} \Lambda\left(\{aij\}, \bar{\gamma}_{\not{s}_k}\right) \\ \times \mathcal{P}_{\perp}^{(s_k)} \left| i_1 j_1, \ldots i_n j_n \right\rangle \bigotimes_{p=-2q}^{-1} \left| a_p \right\rangle , \end{aligned} \tag{5.16}$$

with the sets $S_k^{(n)}$ introduced in eq. (5.10), and having defined

$$\mathcal{P}_{\perp}^{(s_k)} = \bigotimes_{p \in s_k} \mathcal{P}_{\perp}^{(p)} . \tag{5.17}$$

The special case (see eq. (5.12))

$$\mathcal{P}_{\perp}^{(s_0)} = I \,, \qquad s_0 = \{\} \tag{5.18}$$

is understood. Note that, apart from a factor $N^{-\rho(\gamma)}$, the colour factor $\Lambda$ in eq. (5.16) is a string of $n-k+q$ Kronecker $\delta$s. Concerning the $-1/N$ factors mentioned above, they collectively give rise to the $(-1)^k/N^k$ factor on the r.h.s. of eq. (5.16). As one can see, it is extremely easy to keep track of them, by means of the summation index $k$, which essentially counts the number of gluons whose labels are removed (thanks to the second term in the Fierz identity) from the secondary flows. As the colour factors render clear, such gluons become colourless; therefore, they are what in the literature are usually called U(1) gluons. Equation (5.16) gives a geometrical interpretation to such objects. Namely, U(1) gluons are associated with vectors that live in the

$$\bigotimes_{p \in s_k} S_{\{ij\}}^{(p)} \setminus S_{\{a\}}^{(p)} \tag{5.19}$$

JHEP11(2021)045

subspace, which one projects onto by means of $\mathcal{P}_\perp^{(s_k)}$. Therefore, U(1)-gluon vectors are orthogonal to physical-gluon vectors, which belong to the $S_{\{a\}}^{(p)}$ subspaces; it is therefore natural to expect that the interferences between such terms vanish.

The $k = 0$ term in eq. (5.16) is what is usually called the "flow representation" of the given amplitude. That equation clarifies the extent to which this is an improper terminology (strictly, two representations are two different ways for writing the *same* object). As can be seen there, the fundamental and flow representations differ by non-null vectors, which have at least one component in the U(1)-gluon subspaces.

We now turn to computing the analogue of eq. (5.1), i.e. the interference of two amplitudes

$$\mathcal{M}^{(2q;n)}(\gamma',\gamma) = \langle \mathcal{A}^{(2q;n)}(\gamma') | \mathcal{A}^{(2q;n)}(\gamma)\rangle \,. \tag{5.20}$$

Equation (5.16) implies an immediate simplification. By using it to write symbolically

$$\langle \mathcal{A}^{(2q;n)}(\gamma')| = \sum_{k=0}^{n} \sum_{s_k\in S_k^{(n)}} \langle w(s_k)|\mathcal{P}_\perp^{(s_k)} \,, \tag{5.21}$$

one obtains (see eq. (5.18))

$$\mathcal{M}^{(2q;n)}(\gamma',\gamma) = \langle w(s_0) | \mathcal{A}^{(2q;n)}(\gamma)\rangle \,, \tag{5.22}$$

since

$$\mathcal{P}_\perp^{(s_k)} |\mathcal{A}^{(2q;n)}(\gamma)\rangle = 0 \qquad \forall\, s_k \neq s_0 \tag{5.23}$$

owing to the fact that

$$\bigotimes_{p\in s_k} \mathcal{P}_\parallel^{(p)} |\mathcal{A}^{(2q;n)}(\gamma)\rangle = |\mathcal{A}^{(2q;n)}(\gamma)\rangle \,, \tag{5.24}$$

and eq. (4.10). From eq. (5.16) one can read the explicit form of $\langle w(s_0)|$

$$\begin{aligned}\langle w(s_0)| = {}& \widehat{\mathcal{A}}^{(2q;n)}(\gamma')^\star \frac{1}{2^{n/2}} \sum_{\{a_i'\}_{i=-2q}^{-1}} \sum_{\{i_p',j_p'\}_{p=1}^{n}} \Lambda\left(\{a'i'j'\},\bar{\gamma}'_{\not s_0}\right)^\star \\ & \times \langle i_1' j_1', \ldots i_n' j_n'| \bigotimes_{p=-2q}^{-1} \langle a_p'| \,. \end{aligned} \tag{5.25}$$

In the computation of eq. (5.22), one therefore finds

$$\prod_{p=-2q}^{-1} \langle a_p' | a_p\rangle = \prod_{p=-2q}^{-1} \delta_{a_p a_p'} \tag{5.26}$$

and

$$\begin{aligned} & \langle i_1' j_1', \ldots i_n' j_n' |\, \mathcal{P}_\perp^{(s_k)}\, | i_1 j_1, \ldots i_n j_n\rangle \\ & \quad = \langle i_1' j_1', \ldots i_n' j_n' |\, \mathcal{P}_\perp^{(s_k)^2}\, | i_1 j_1, \ldots i_n j_n\rangle \\ & \quad = \frac{1}{N^k} \prod_{p'\in s_k} \delta_{i_{p'}' j_{p'}'} \prod_{p\in s_k} \delta_{i_p j_p} \prod_{r\in s_k} \langle \perp_r | \perp_r\rangle \prod_{s\notin s_k} \langle i_s' j_s' | i_s j_s\rangle \\ & \quad = \frac{1}{N^k} \prod_{p'\in s_k} \delta_{i_{p'}' j_{p'}'} \prod_{p\in s_k} \delta_{i_p j_p} \prod_{s\notin s_k} \delta_{i_s' i_s} \delta_{j_s' j_s} \,, \end{aligned} \tag{5.27}$$

JHEP11(2021)045

having employed the leftmost part of eq. (4.11). The colour factor $\Lambda(\{aij\}, \bar{\gamma}_{\not{s}_k})$ in eq. (5.16) does not depend on any of the indices $i_p$ and $j_p$ if $p \in s_k$. One can therefore carry out the sums over these indices, exploiting the second Kronecker $\delta$ in eq. (5.27): this results in a factor $N^k$, that cancels an identical factor in the denominator of eq. (5.27). Conversely, the dependence on the indices that appear in the leftmost Kronecker $\delta$ in eq. (5.27) is non trivial, owing to the colour factor in eq. (5.25). However, it can be simplified by observing that

$$\sum_{\{i'_p,j'_p\}_{p=1}^{n}} \Lambda\left(\{a'i'j'\}, \bar{\gamma}'_{\not{s}_0}\right)^{\star} \prod_{p\in s_k} \delta_{i'_p j'_p} = \sum_{\{i'_p,j'_p\}_{p\notin s_k}} \Lambda\left(\{a'i'j'\}, \bar{\gamma}'_{\not{s}_k}\right)^{\star} . \tag{5.28}$$

By putting everything back together, and by exploiting the rightmost Kronecker $\delta$ in eq. (5.27) one finally obtains

$$\begin{aligned}\mathcal{M}^{(2q;n)}(\gamma',\gamma) = \widehat{\mathcal{A}}^{(2q;n)}(\gamma')^{\star}\widehat{\mathcal{A}}^{(2q;n)}(\gamma) \sum_{k=0}^{n} \sum_{s_k\in S_k^{(n)}} \frac{(-1)^k}{2^n} N^{-k} \\ \times \sum_{\{a_i\}_{i=-2q}^{-1}} \sum_{\{i_p j_p\}_{p\notin s_k}} \Lambda\left(\{aij\}, \bar{\gamma}'_{\not{s}_k}\right)^{\star} \Lambda\left(\{aij\}, \bar{\gamma}_{\not{s}_k}\right) .\end{aligned} \tag{5.29}$$

The colour factor in eq. (5.29) has the same form as any colour factor in a quark-only process. By using the result of section 5.1, one obtains

$$\begin{aligned}&\sum_{\{a_i\}_{i=-2q}^{-1}} \sum_{\{i_p j_p\}_{p\notin s_k}} \Lambda\left(\{aij\}, \bar{\gamma}'_{\not{s}_k}\right)^{\star} \Lambda\left(\{aij\}, \bar{\gamma}_{\not{s}_k}\right) \\ &\qquad = N^{-\rho(\gamma)-\rho(\gamma')} N^{\left|\mathcal{L}(\bar{\gamma}'_{\not{s}_k}, \bar{\gamma}_{\not{s}_k})\right|} ,\end{aligned} \tag{5.30}$$

and therefore the sought final result

$$\begin{aligned}\mathcal{M}^{(2q;n)}(\gamma',\gamma) = \widehat{\mathcal{A}}^{(2q;n)}(\gamma')^{\star}\widehat{\mathcal{A}}^{(2q;n)}(\gamma) N^{-\rho(\gamma)-\rho(\gamma')} \\ \sum_{k=0}^{n} \sum_{s_k\in S_k^{(n)}} \frac{(-1)^k}{2^n} N^{-k} N^{\left|\mathcal{L}(\bar{\gamma}'_{\not{s}_k}, \bar{\gamma}_{\not{s}_k})\right|} .\end{aligned} \tag{5.31}$$

The similarity of this result to that of eq. (5.1) is striking. It implies that quark-gluon matrix elements have essentially the same structure as quark-only ones, provided one expresses them by means of secondary colour flows, thereby introducing U(1) gluons into the picture. The role of the latter is quite prominent in eq. (5.31): their number is equal to the summation index $k$, and they induce alternating signs ($(-1)^k$) in the summands. We stress that this is true also on the diagonal of the colour-flow matrix (i.e. when $\gamma' = \gamma$), which is a real number squared. Furthermore, U(1) gluons give eq. (5.31) a hierarchy in powers of $1/N$. Unfortunately, this hierarchy is not absolute for off-diagonal elements. This is due to the fact that while

$$\left|\mathcal{L}(\bar{\gamma}_{\not{s}_k}, \bar{\gamma}_{\not{s}_k})\right| \geq \left|\mathcal{L}(\bar{\gamma}_{\not{s}_l}, \bar{\gamma}_{\not{s}_l})\right| \qquad \text{when} \quad k < l\,, \tag{5.32}$$

an analogous inequality does not hold true in general for a generic off-diagonal $(\gamma', \gamma)$ closed flow.

### 5.2.1 Summing over flows

The matrix elements relevant to a given closed flow given in eq. (5.31) need be summed, in most physical applications, over flows. Owing to the summations over secondary flows, this operation is less trivial than its quark-only counterpart, which had led immediately to the result of eq. (5.2). We perform it in this section, showing in the process that it leads naturally to the definition of linear combinations of dual amplitudes where U(1) gluons play a special role.

The relevant summations can be carried out directly on the scalar quantities on the r.h.s. of eq. (5.31); this implies two sums, as both the L- and R-flows must be summed. It is therefore more convenient to arrive at the sought result by summing amplitudes, as opposed to interferences of amplitudes, since this entails carrying out a single sum; in other words, we exploit eq. (2.26) rather than the rightmost side of eq. (2.27). Thus, we consider

$$|\mathcal{A}^{(2q;n)}\rangle = \sum_{\gamma\in\mathcal{F}_{2q;n}} |\mathcal{A}^{(2q;n)}(\gamma)\rangle\,, \tag{5.33}$$

where the summands on the r.h.s. are given in eq. (5.16). The key idea is the following: so far, secondary colour flows are quantities derived from some given colour flows. However, they have a structure which is perfectly well-defined in its own right; therefore, when a sum over flows has to be carried out, it is possible to exchange the order in which the sums over flows and secondary flows are performed, thus inverting the logic, since the former are now seen as quantities derived from the latter.

In order to do this, one starts by introducing a notation for the sets of $q$-quark, $(n-k)$-gluon colour flows as follows:

$$\begin{aligned} s_k = \{j_1,\ldots j_k\} \in S_k^{(n)} \\ \longrightarrow\quad \mathcal{F}^{(s_k)}_{2q;n-k} \equiv \mathcal{F}_{2q;n-k}\Big(\text{gluon labels in } \{1,\ldots n\}\setminus\{j_1,\ldots j_k\}\Big)\,. \end{aligned} \tag{5.34}$$

Thus, $\mathcal{F}^{(s_k)}_{2q;n-k}$ is identical to $\mathcal{F}_{2q;n-k}$ up to the labels of the gluons that enter its flows, which must be different from the indices $j_i$ that belong to the given set $s_k$. This implies

$$\bar{\gamma}_{\not{s}_k} \in \mathcal{F}^{(s_k)}_{2q;n-k}\,. \tag{5.35}$$

With this, one can generalize the operator $I_+$ of ref. [23] (see appendix B there). In particular

$$\begin{aligned} s_k \in S_k^{(n)}\,, \bar{\gamma} \in \mathcal{F}^{(s_k)}_{2q;n-k}\,, \{i_1,\ldots i_k\} \subseteq \{-1,\ldots -q\} \cup \{1,\ldots n\} \\ \longrightarrow\quad I_+^{(s_k)}(i_1,\ldots i_k)\bar{\gamma} = I_+^{(j_1)}(i_1) I_+^{(j_2)}(i_2)\ldots I_+^{(j_k)}(i_k)\bar{\gamma}\,, \end{aligned} \tag{5.36}$$

for any $k \ge 1$; when $k=0$, $I_+$ is defined to be the identity. The single-label $I_+$ operator employed $k$ times on the rightmost side of eq. (5.36) coincides with that ref. [23], except for a minor notational detail: here, the upper index $j_\alpha$ indicates the label of the gluon to be inserted to the right of position $i_\alpha$; this index was not necessary in ref. [23], because $j_\alpha = n+1$ there. Implicit in eq. (5.36) is the fact that the $I_+$ operators are to be used

JHEP11(2021)045

in a right-to-left order: first the gluon with label $j_k$ is inserted, then that with label $j_{k-1}$, and so forth up to $j_1$. As far as the values that the position indices $i_\alpha$ can assume are concerned: for the first gluon to be inserted ($j_k$) one has $q+n-k$ possibilities (i.e. to the right of each quark and of each gluon in the given flow $\bar{\gamma}$); for the second one, the possibilities are $q+n-k+1$, since that gluon is now inserted in the flow $I_+^{(j_k)}(i_k)\bar{\gamma}$ which has one additional gluon w.r.t. the original one $\bar{\gamma}$. The procedure is then iterated till the last gluon to be inserted ($j_1$). Since the $I_+$ operator of eq. (5.36) is single-valued given the position parameters $i_\alpha$, the dimension of the set that contains all possible flows generated by $I_+$ acting on a given $\bar{\gamma}$ is equal to the dimension of the codomain of the acceptable values of the $i_\alpha$ parameters. We have therefore

$$\left|\bigcup_{i_1\ldots i_k}\left\{I_+^{(s_k)}(i_1,\ldots i_k)\bar{\gamma}\right\}\right| = (n+q-1)(n+q-2)\ldots(n+q-k)\,, \tag{5.37}$$

for any given flow $\bar{\gamma}\in\mathcal{F}_{2q;n-k}^{(s_k)}$. Also

$$\bar{\gamma}\,,\bar{\gamma}'\in\mathcal{F}_{2q;n-k}^{(s_k)}\,,\quad \bar{\gamma}\neq\bar{\gamma}'\quad\Longrightarrow\quad I_+^{(s_k)}(i_1,\ldots i_k)\bar{\gamma}\neq I_+^{(s_k)}(i_1,\ldots i_k)\bar{\gamma}\,. \tag{5.38}$$

Finally

$$I_+^{(s_k)}(i_1,\ldots i_k)\bar{\gamma}\in\mathcal{F}_{2q;n}\,. \tag{5.39}$$

Taken together, the above facts imply that

$$\mathcal{F}_{2q;n} = \bigcup_{\bar{\gamma}\in\mathcal{F}_{2q;n-k}^{(s_k)}}\;\bigcup_{i_1\ldots i_k}\left\{I_+^{(s_k)}\left(i_1,\ldots i_k\right)\bar{\gamma}\right\}\,,\qquad\forall\,0\le k\le n\;\;\text{and}\;\;s_k\in S_k^{(n)}\,, \tag{5.40}$$

and

$$\left(I_+^{(s_k)}\left(i_1,\ldots i_k\right)\bar{\gamma}\right)_{\not{s_k}} = \bar{\gamma}\,,\qquad\forall\,\bar{\gamma}\in\mathcal{F}_{2q;n-k}^{(s_k)}\;\;\text{and}\;\;\{i_1,\ldots i_k\}\,. \tag{5.41}$$

Therefore, for any function $F$ that is summed over all flows and depends on a $q$-quark, $n$-gluon flow and on one of its secondary flows, the following identity holds:

$$\sum_{\gamma\in\mathcal{F}_{2q;n}} F\left(\gamma,\bar{\gamma}_{\not{s_k}}\right) = \sum_{\bar{\gamma}\in\mathcal{F}_{2q;n-k}^{(s_k)}}\;\sum_{i_1\ldots i_k} F\left(I_+^{(s_k)}(i_1,\ldots i_k)\bar{\gamma},\bar{\gamma}\right)\,, \tag{5.42}$$

for any $k$ and $s_k\in S_k^{(n)}$; the number of summands on the two sides of eq. (5.42) is the same, owing to eqs. (2.11), (5.37), and (5.40).

We now consider the sum on the r.h.s. of eq. (5.33), by replacing the amplitude there with its expression given in eq. (5.16). There are no cross-constraints between the sum over $\gamma$ and those over $k$ and $s_k$, and this implies the latter two can be used as the outermost ones, which leads us to the following result:

$$\begin{aligned}|\mathcal{A}^{(2q;n)}\rangle = \sum_{k=0}^{n}\sum_{s_k\in S_k^{(n)}}\frac{(-1)^k}{2^{n/2}N^k}\sum_{\gamma\in\mathcal{F}_{2q;n}}\widehat{\mathcal{A}}^{(2q;n)}(\gamma)\sum_{\{a_i\}_{i=-2q}^{-1}}\sum_{\{i_p,j_p\}_{p=1}^{n}}\Lambda\left(\{aij\}\,,\bar{\gamma}_{\not{s_k}}\right)\\ \times\,\mathcal{P}_\perp^{(s_k)}\,|i_1j_1,\ldots i_nj_n\rangle\bigotimes_{p=-2q}^{-1}|a_p\rangle\,.\end{aligned} \tag{5.43}$$

JHEP11(2021)045

In this expression, the part to the right of the sum over $\mathcal{F}_{2q;n}$ (including it) is in the same form as the l.h.s. of eq. (5.42). Thus, by employing the r.h.s. of that equation instead we arrive at

$$|\mathcal{A}^{(2q;n)}\rangle = \sum_{k=0}^{n} \sum_{s_k \in S_k^{(n)}} \frac{(-1)^k}{2^{n/2} N^k} \sum_{\bar{\gamma} \in \mathcal{F}_{2q;n-k}^{(s_k)}} \bar{\mathcal{A}}^{(2q;n)}(\bar{\gamma}) \sum_{\{a_i\}_{i=-2q}^{-1}} \sum_{\{i_p, j_p\}_{p=1}^{n}} \Lambda(\{aij\}, \bar{\gamma})$$
$$\times \mathcal{P}_{\perp}^{(s_k)} |i_1 j_1, \ldots i_n j_n\rangle \bigotimes_{p=-2q}^{-1} |a_p\rangle , \tag{5.44}$$

where we have defined the following linear combinations of the standard (because of eq. (5.39)) dual amplitudes:

$$\bar{\gamma} \in \mathcal{F}_{2q;n-k}^{(s_k)} \quad \Longrightarrow \quad \bar{\mathcal{A}}^{(2q;n)}(\bar{\gamma}) = \sum_{i_1 \ldots i_k} \widehat{\mathcal{A}}^{(2q;n)} \left( I_+^{(s_k)}(i_1, \ldots i_k) \bar{\gamma} \right) . \tag{5.45}$$

In words: for a given $q$-quark-antiquark, $(n-k)$-gluon colour flow $\bar{\gamma}$, an amplitude $\bar{\mathcal{A}}^{(2q;n)}(\bar{\gamma})$ is associated with it that is obtained by summing the dual amplitudes relevant to the colour flows constructed by inserting $k$ gluons in $\bar{\gamma}$ in all possible manners. Those extra gluons are colourless as far as the total amplitude is concerned, which is consistent with their (implicit) role in $\bar{\gamma}$ as U(1) gluons.

Starting from eq. (5.44), we can repeat the procedure that has brought us from eq. (5.20) to eq. (5.31), to obtain

$$\mathcal{M}^{(2q;n)} = \sum_{k=0}^{n} \sum_{s_k \in S_k^{(n)}} \frac{(-1)^k}{2^n} N^{-k}$$
$$\times \sum_{\bar{\gamma}, \bar{\gamma}' \in \mathcal{F}_{2q;n-k}^{(s_k)}} \bar{\mathcal{A}}^{(2q;n)}(\bar{\gamma}')^{\star} \, \bar{\mathcal{A}}^{(2q;n)}(\bar{\gamma}) \, N^{-\rho(\bar{\gamma})-\rho(\bar{\gamma}')} N^{|\mathcal{L}(\bar{\gamma}', \bar{\gamma})|} . \tag{5.46}$$

As was already the case for the matrix elements at given closed colour flow (eq. (5.31)), eq. (5.46) is very similar to its quark-only counterpart, eq. (5.2). The same comments made about eq. (5.31) vs eq. (5.1) in this regard apply here, with the additional observations on the $\bar{\mathcal{A}}^{(2q;n)}(\bar{\gamma})$ amplitudes of eq. (5.45). Note that eqs. (5.31) and (5.46) share an important feature, namely: not only do quantities associated with different numbers of U(1) gluons not interfere with each other but also, for the interference to have a non-zero result, the identities of such gluons must be the same on both sides of the cut (technically, the set $s_k$ is relevant to both the L- and R-flow).

We remark that expressions whose content is analogous to that of eq. (5.44) appear in ref. [19], where use is made of the same linear combinations of dual amplitudes as in eq. (5.45). What we have shown here is that: *a)* at the level of amplitudes, the structure of eq. (5.44) is already present at fixed colour flow (eq. (5.16)); *b)* the number of summands associated with different sets of U(1) gluons (the differences being in their numbers and/or identities) is equal to $2^n$ (see eq. (5.14)) at the level of both the amplitudes (eqs. (5.16) and (5.44)) *and the matrix elements* (eqs. (5.31) and (5.46)). This fact is non-trivial: one

JHEP11(2021)045

can show that by naively (i.e. without using the properties of projectors) squaring eq. (5.44) to obtain eq. (5.46), an individual term with $k$ U(1) gluons in the latter expression results from the sum of $3^k$ terms, $3^k - 1$ of which mutually cancel.

### 5.3 Gluon-only matrix elements

The results of section 5.2 show that ultimately gluons can be made to behave as extra quark-antiquark pairs in quark-gluon amplitudes. This is a strong hint that the same property will hold true also in the case of gluon-only amplitudes, i.e. when there are no quarks or antiquarks to start with. In this section, we shall prove that this is indeed the case. At the end of the day this will not matter when summing over colour flows, since in that case Ward identities will ensure that U(1)-gluon contributions vanish. However, at fixed flows U(1) gluons do play a non-trivial role, which puts gluon-only amplitudes on the same footing as quark-only ones, as has already happened with quark-gluon amplitudes.

Let

$$\sigma = (\sigma(1), \ldots \sigma(n)) \tag{5.47}$$

be a generic $n$-gluon colour flow. We define two operators, $q\mathrm{R}$ and $q\mathrm{L}$, that by acting on $\sigma$ result in the following 2-quark-antiquark, $n$-gluon colour flows:

$$q\mathrm{R}\,(\sigma) \equiv \sigma_{q\mathrm{R}} = (-1; \sigma\,(1)\,, \ldots \sigma\,(n)\,; -4)\,(-2; -3)\ , \tag{5.48}$$

$$q\mathrm{L}\,(\sigma) \equiv \sigma_{q\mathrm{L}} = (-1; -4)\,(-2; \sigma(1), \ldots \sigma(n); -3)\ . \tag{5.49}$$

Here, following the conventions of eqs. (2.2)–(2.4), we have introduced two quark-antiquark pairs of different flavours, labelled by $(-1, -3)$ and $(-2, -4)$. Thus, according to eq. (2.14) (see also footnote 5), the associated colour factors are

$$\Lambda(\{a_i\}, \sigma_{q\mathrm{R}}) = \left(\lambda^{a_1}\lambda^{a_{\sigma(2)}} \ldots \lambda^{a_{\sigma(n)}}\right)_{a_{-1}a_{-4}} \delta_{a_{-2}a_{-3}}\,, \tag{5.50}$$

$$\Lambda(\{a_i\}, \sigma_{q\mathrm{L}}) = \delta_{a_{-1}a_{-4}}\left(\lambda^{a_1}\lambda^{a_{\sigma(2)}} \ldots \lambda^{a_{\sigma(n)}}\right)_{a_{-2}a_{-3}}\,, \tag{5.51}$$

which among other things rely on the fact that

$$\rho\Big(q\mathrm{R}(\sigma)\Big) = \rho\Big(q\mathrm{L}(\sigma)\Big) = 0 \tag{5.52}$$

for any $\sigma$. With these definitions, for any closed colour flow $(\sigma', \sigma)$ we have

$$C\,(\sigma', \sigma) \equiv \sum_{\{a_i\}_{i=1}^{n}} \Lambda\,(\{a_i\}, \sigma')^\star\,\Lambda\,(\{a_i\}, \sigma) \tag{5.53}$$

$$= C\left(\sigma'_{q\mathrm{L}}, \sigma_{q\mathrm{R}}\right) \equiv \sum_{\{a_i\}_{i=-4}^{n}} \Lambda\left(\{a_i\}, \sigma'_{q\mathrm{L}}\right)^\star \Lambda(\{a_i\}, \sigma_{q\mathrm{R}})\,. \tag{5.54}$$

JHEP11(2021)045

Furthermore, the definitions of eqs. (5.48) and (5.49) respect the colour-loop structure. In other words, by using the results of section 3, it is straightforward to prove the following properties:

- The closed colour flow $(\sigma', \sigma)$ has the same number of colour loops as $(\sigma'_{q\mathrm{L}}, \sigma_{q\mathrm{R}})$

$$\left|\mathcal{L}\left(\sigma', \sigma\right)\right| = \left|\mathcal{L}\left(\sigma'_{q\mathrm{L}}, \sigma_{q\mathrm{R}}\right)\right| . \tag{5.55}$$

- The colour loops in $\mathcal{L}(\sigma', \sigma)$ and $\mathcal{L}(\sigma'_{q\mathrm{L}}, \sigma_{q\mathrm{R}})$ are pairwise identical, except for that which contains $\sigma(n)$ as an R-element, and that which contains $\sigma'(1)$ as an L-element;[10] these differ *only* because the loops in $\mathcal{L}(\sigma'_{q\mathrm{L}}, \sigma_{q\mathrm{R}})$ include the fictitious quarks and antiquarks labelled $-1, \ldots -4$.

- Given any loop $\ell_1 \in \mathcal{L}(\sigma', \sigma)$, let $\ell_2 \in \mathcal{L}(\sigma'_{q\mathrm{L}}, \sigma_{q\mathrm{R}})$ be the loop corresponding to it according to the previous item, so that for any two gluon labels $k$ and $l$ we have

$$k, l \in \ell_1 \quad \Longleftrightarrow \quad k, l \in \ell_2 \, . \tag{5.56}$$

Then the distance introduced in eq. (3.3) fulfills the property

$$\delta_{\ell_1}(k, l) = \delta_{\ell_2}(k, l) + 2i \, , \tag{5.57}$$

with $i$ an integer. In other words, the distance between $k$ and $l$ has the same parity in the $\ell_1$ and $\ell_2$ loops.

Equations (5.53)–(5.57) are sufficient to allow one to repeat, with only notational changes, what has been done in section 5.2. The analogue of eq. (5.16) reads as follows:

$$\begin{aligned} \left|\mathcal{A}^{(n)}(\sigma)\right\rangle = \widehat{\mathcal{A}}^{(n)}(\sigma) \sum_{k=0}^{n} \sum_{s_k \in S_k^{(n)}} \frac{(-1)^k}{2^{n/2} N^k} \sum_{\{a_i\}_{i=-4}^{-1}} \sum_{\{i_p, j_p\}_{p=1}^{n}} \Lambda\left(\{aij\}, \bar{\sigma}_{q\mathrm{R}\not{s}_k}\right) \\ \times \mathcal{P}_{\perp}^{(s_k)} \left|i_1 j_1, \ldots i_n j_n\right\rangle \bigotimes_{p=-4}^{-1} \left|a_p\right\rangle . \end{aligned} \tag{5.58}$$

Note a technical peculiarity: because of the necessity to use the $q\mathrm{R}$ and $q\mathrm{L}$ operators, $|\mathcal{A}^{(n)}(\sigma)\rangle$ is a vector that lives in the

$$\bigotimes_{p=-4}^{-1} S_{\{a\}}^{(p)} \bigotimes_{p=1}^{n} S_{\{ij\}}^{(p)} \tag{5.59}$$

vector space. However, the fictitious-quark degrees of freedom only play the role of giving the correct normalization and index-contraction conditions when an interference of amplitudes is considered. More significantly, the contributions to eq. (5.58) with $k \geq 1$ (i.e. with at least one U(1) gluon) do *not* vanish. This is contrary to what one normally expects, but in fact that expectation is based on results fully summed over colour degrees of freedom. In

[10] These loops may actually coincide.

JHEP11(2021)045

other words, not summing over those, and fixing e.g. the colour flow, the contributions due to U(1) gluons are non-trivial. The implication of this is that, at fixed flows, the flow and the fundamental representations do not coincide for gluon-only amplitudes, thus making the latter behave exactly as their quark-gluon counterparts. As we shall see later, this symmetry is lifted upon summing over flows, and this thanks to the dual Ward identities, which have no analogue in the quark-gluon case.

By interfering the amplitude of eq. (5.58) with an analogous one on the left-hand side of the cut (relevant to an L-flow equal to $\sigma'$), one obtains the analogue of eq. (5.31), namely

$$\begin{aligned} \mathcal{M}^{(n)}(\sigma',\sigma) &= \widehat{\mathcal{A}}^{(n)}(\sigma')^\star \widehat{\mathcal{A}}^{(n)}(\sigma) \\ &\times \sum_{k=0}^{n} \sum_{s_k \in S_k^{(n)}} \frac{(-1)^k}{2^n} N^{-k} N^{\left|\mathcal{L}(\bar{\sigma}'_{q\mathrm{L}\,\not{s}_k},\bar{\sigma}_{q\mathrm{R}\,\not{s}_k})\right|}\,, \end{aligned} \tag{5.60}$$

having used eq. (5.52). As was the case at the amplitude level, U(1) gluons do contribute non-trivially to eq. (5.60).

### 5.3.1 Summing over flows

The sum over colour flows of the results of eqs. (5.58) and (5.60) follows the same steps as in section 5.2.1. Therefore, one readily arrives at the analogue of eq. (5.44), namely

$$\begin{aligned} |\mathcal{A}^{(n)}\rangle = \sum_{k=0}^{n} \sum_{s_k \in S_k^{(n)}} \frac{(-1)^k}{2^{n/2} N^k} \sum_{\bar{\sigma} \in P'^{(s_k)}_{n-k}} \bar{\mathcal{A}}^{(n)}(\bar{\sigma}) \sum_{\{a_i\}_{i=-4}^{-1}} \sum_{\{i_p, j_p\}_{p=1}^{n}} \Lambda\left(\{aij\},\bar{\sigma}\right) \\ \times \mathcal{P}_\perp^{(s_k)} \left| i_1 j_1, \ldots i_n j_n \right\rangle \bigotimes_{p=-4}^{-1} |a_p\rangle\,, \end{aligned} \tag{5.61}$$

where, similarly to what was done in eq. (5.45), we have been led to define

$$\bar{\sigma} \in P'^{(s_k)}_{n-k} \quad \Longrightarrow \quad \bar{\mathcal{A}}^{(n)}(\bar{\sigma}) = \sum_{i_1 \ldots i_k} \widehat{\mathcal{A}}^{(n)}\left(I_+^{(s_k)}(i_1,\ldots i_k)\bar{\sigma}\right)\,. \tag{5.62}$$

In eqs. (5.61) and (5.62), by $P'^{(s_k)}_{n-k}$ we have denoted the analogue of $\mathcal{F}^{(s_k)}_{2q;n-k}$, i.e. the set of $(n-k)$-gluon colour flows with indices not equal to any of the integers that belong to the set $s_k$ (the presence of the fictitious quark labels is understood, and ignored notation-wise). Furthermore, the $I_+$ operators that appear on the r.h.s. of eq. (5.62) are defined in a manner formally identical to that of eq. (5.36), with the condition that the indices $i_\alpha$ can only assume positive values here; this implies that no gluon will be inserted to the right of either quark[11] in $\bar{\sigma}$. This constraint is necessary in order to preserve the cyclicity properties of gluon-only flows. Because of it, one observes that, for any given values of $i_2 \ldots i_k$, the sum over $i_1$ in eq. (5.62) features $n-1$ terms, which are exactly those that enter into the dual Ward identity. Therefore

$$\bar{\mathcal{A}}^{(n)}(\bar{\sigma}) = 0\,, \qquad \forall\, k > 0\,, \tag{5.63}$$

$$\bar{\mathcal{A}}^{(n)}(\bar{\sigma}) = \widehat{\mathcal{A}}^{(n)}(\bar{\sigma})\,, \qquad k = 0\,, \tag{5.64}$$

[11] An exception must be made when $k = n$, when the first gluon is inserted in the only possible place, i.e. onto the then-empty first (second) quark line in the secondary R-flow (L-flow).

JHEP11(2021)045

and eq. (5.61) simplifies to read

$$|\mathcal{A}^{(n)}\rangle = \frac{1}{2^{n/2}} \sum_{\bar\sigma \in P'_n} \widehat{\mathcal{A}}^{(n)}(\bar\sigma) \sum_{\{i_p,j_p\}_{p=1}^n} \Lambda\left(\{ij\},\bar\sigma\right) |i_1 j_1,\ldots i_n j_n\rangle\,. \tag{5.65}$$

This is the anticipated, and expected, result: when the sum over flows is carried out, all of the contributions due to U(1) gluons (which are non-zero vectors that live in the $S^{(p)}_{\{ij\}} \setminus S^{(p)}_{\{a\}}$ subspaces, with $p = 1\ldots n$) add to zero. One must bear this fact in mind when claiming that, for gluon-only amplitudes, the fundamental and the flow representation are strictly equivalent.

One can finally use eq. (5.65) to arrive at the result, analogous to that of eq. (5.46), for the matrix elements

$$\mathcal{M}^{(n)} = \frac{1}{2^n} \sum_{\bar\sigma,\bar\sigma' \in P'_n} \widehat{\mathcal{A}}^{(n)}(\bar\sigma')^\star \, \widehat{\mathcal{A}}^{(n)}(\bar\sigma)\, N^{|\mathcal{L}(\bar\sigma',\bar\sigma)|}\,, \tag{5.66}$$

which again is as expected. Although the notation of eq. (5.66) bears memory of the usage of secondary flows, in the absence of U(1) gluons, and in view of the definition of colour loops adopted here, there is no difference between the original flows and the secondary ones in eq. (5.66).

## 6 Soft limits

We consider here the tree-level matrix elements relevant to processes that feature at least one gluon, in the limit in which one of such gluons is soft, while all of the other partons are hard and well separated from each other.

These soft limits have been studied in detail in ref. [23]; we shall show below how the formulae of that paper assume an extremely simple form with a geometrical character by exploiting what has been done in section 5. The results of ref. [23] were derived with the goal of employing them in the FKS subtraction formalism [30, 31] for the computation of NLO-accurate cross sections and, to that end, were given in three different forms, namely: at fixed colour configurations, and at fixed colour flows either at the real-emission or at the Born level. Since the emphasis of the current paper is on PSMCs, working at fixed colour configurations is of no interest. Fixing the colour flows at the real-emission level is also problematic. Such kinematics configurations are those one obtains after the first emission in a PSMC; therefore, information gathered from the matrix elements at that level cannot easily be used by the PSMC to generate those very same emissions. In keeping with its Markovian structure, a shower will generate emissions in its $k^{th}$ step by using information available at the $(k-1)^{th}$ step. The most convenient option, then, is to work at fixed Born-level flows, which is what we are going to do in the present section.

Consistently with the previous statement, when referring to processes that feature $n$ gluons we understand those gluon to be at the Born level. In particular, a quark-only case is that of the soft limit of a matrix element relevant to those quarks plus an additional gluon that becomes soft.

JHEP11(2021)045

### 6.1 Quark-only matrix elements

In this case, the quantity of interest is that in eq. (**I**.3.86) (with $n = 0$), which we report here for convenience

$$\mathcal{M}^{(2q;1)}_{\text{SOFT}}(\gamma', \gamma) = \frac{1}{2} g_S^2 \sum_{k,l=-2q}^{-1} \Big[k, l\Big] \mathcal{M}^{(2q;0)}_{kl}(\gamma', \gamma) \ . \tag{6.1}$$

The r.h.s. of this equation features the usual eikonal factors,

$$\Big[k, l\Big] \equiv \frac{(p_k \cdot p_l)}{(p \cdot p_k)(p \cdot p_l)} \tag{6.2}$$

where $p$ is the four-momentum of the emitted soft gluon, $p_k$ and $p_l$ are those of the corresponding quarks or antiquarks, and $\mathcal{M}^{(2q;0)}_{kl}(\gamma', \gamma)$ is the colour-linked matrix element for the given closed flow. The latter have been presented in eq. (**I**.3.115); again we report here their expressions

$$\begin{aligned}\mathcal{M}^{(2q;0)}_{kl}(\gamma', \gamma) = -2\,\widehat{\mathcal{A}}^{(2q;0)}(\gamma')^\star \Big[ & C\left(I_+(\gamma'^{-1}(k))\,\gamma',\, I_+(\gamma^{-1}(l))\,\gamma\right)(1-\delta_{\bar{q}\mathcal{I}_k})\,(1-\delta_{\bar{q}\mathcal{I}_l}) \\ & - C\left(I_+(\gamma'^{-1}(k))\,\gamma',\, I_-(\gamma^{-1}(l))\,\gamma\right)(1-\delta_{\bar{q}\mathcal{I}_k})\,(1-\delta_{q\mathcal{I}_l}) \\ & - C\left(I_-(\gamma'^{-1}(k))\,\gamma',\, I_+(\gamma^{-1}(l))\,\gamma\right)(1-\delta_{q\mathcal{I}_k})\,(1-\delta_{\bar{q}\mathcal{I}_l}) \\ & + C\left(I_-(\gamma'^{-1}(k))\,\gamma',\, I_-(\gamma^{-1}(l))\,\gamma\right)(1-\delta_{q\mathcal{I}_k})\,(1-\delta_{q\mathcal{I}_l}) \Big] \\ \times\, \widehat{\mathcal{A}}^{(2q;0)}(\gamma)\,. & \end{aligned} \tag{6.3}$$

Fuller details can be found in ref. [23]. It turns out that the explicit computation of eq. (6.1) leads to the following simple result:

$$\begin{aligned}\mathcal{M}^{(2q;1)}_{\text{SOFT}}(\gamma', \gamma) = 2 g_S^2\, \widehat{\mathcal{A}}^{(2q;0)}(\gamma')^\star \widehat{\mathcal{A}}^{(2q;0)}(\gamma)\, N^{-\rho(\gamma)-\rho(\gamma')} N^{|\mathcal{L}(\gamma',\gamma)|} \\ \times\, C_F \sum_{\ell \in \mathcal{L}(\gamma',\gamma)} \sum_{k,l \in \ell}^{k<l} (-1)^{\delta_\ell(k,l)} \Big[k, l\Big]\,. \end{aligned} \tag{6.4}$$

The proof of eq. (6.4) proceeds as follows. Start by considering eq. (6.3): of the four terms on the r.h.s., only one may not be equal to zero, owing to the fact that both $k$ and $l$ are either a quark or an antiquark, and the conclusion follows from the presence of the Kronecker $\delta$'s. Thus, a single colour factor is relevant, which we write as follows (the notation is symbolic: the appropriate choices of the signs where $\pm$ appear are given in eq. (6.3))

$$\pm\, C\left(I_\pm(\gamma'^{-1}(k))\,\gamma',\, I_\pm(\gamma^{-1}(l))\,\gamma\right) . \tag{6.5}$$

The general form of eq. (6.5) can be obtained from eq. (2.14). Taking footnote 5 into account, and denoting by $a$ the colour of the only gluon present in the process, there are only two possibilities for the colour factor of eq. (6.5), namely

$$\pm C\left(I_\pm(\gamma'^{-1}(k))\,\gamma',\, I_\pm(\gamma^{-1}(l))\,\gamma\right) \ni \sum_a \text{Tr}\,(\lambda^a)\,\text{Tr}\,(\lambda^a) = 0\,, \tag{6.6}$$

$$\pm C\left(I_\pm(\gamma'^{-1}(k))\,\gamma',\, I_\pm(\gamma^{-1}(l))\,\gamma\right) \ni \sum_a \text{Tr}\,(\lambda^a \lambda^a) = C_F\,\text{Tr}\,(I)\ . \tag{6.7}$$

JHEP11(2021)045

The case of eq. (6.6) (eq. (6.7)) occurs when the operators $I_\pm$ insert the gluon onto open colour lines which do not (do) belong to the same closed colour line.[12] It is easy to see that this happens when the partons $k$ and $l$ do not (do) belong to the same Born-level colour loop. This explains the two summations that appear on the r.h.s. of eq. (6.4). By considering the only non-trivial case, eq. (6.7), one sees that the rightmost side is equivalent to removing the gluon from the closed colour line where it appears (with the factor $C_F$ accounting for it); the remaining trace of the identity is the contribution of that closed colour line stripped of the gluon (which now contains only quarks and antiquarks, and thus coincides with a colour loop) to the overall colour factor. Then, since the other closed colour lines have not been affected by the $I_\pm$ operators, and are therefore identical to their Born-level colour-loop counterparts, eq. (6.7) can be written more precisely as follows

$$\begin{aligned}\pm C\left(I_\pm({\gamma'}^{-1}(k))\,\gamma',\, I_\pm(\gamma^{-1}(l))\,\gamma\right) &= \pm C_F\, C\left(\gamma',\gamma\right)\\ &= \pm C_F\, N^{-\rho(\gamma)-\rho(\gamma')} N^{|\mathcal{L}(\gamma',\gamma)|}\,,\end{aligned} \tag{6.8}$$

having repeated the procedure that led us to eq. (5.1). Equation (6.8) essentially completes the proof of eq. (6.4), bar the sign in front of each eikonal. In order to establish that, one observes that the sign in front of each colour-flow matrix element on the r.h.s. of eq. (6.3) (taking the overall minus sign into account) is equal to $-1$ if $k$ and $l$ are both quarks or both antiquarks, and equal to $+1$ if one of $k$ and $l$ is a quark and the other an antiquark. According to item ii. in section 3 the former case is that of $k$ and $l$ being both R-elements or L-elements, while the latter case is that of one R-element and one L-element. As such, in the former (latter) case $k$ and $l$ are separated by an odd (even) number of partons. By putting all this together, one obtains the factor $(-1)^{\delta_\ell(k,l)}$ on the r.h.s. of eq. (6.4). Finally, the factor $1/2$ in eq. (6.1) is necessary to avoid double counting, the summands on the r.h.s. being symmetric when $k \leftrightarrow l$; such a symmetry is removed in the sum on the r.h.s. of eq. (6.4) (owing to the $k < l$ constraint), and therefore no factor $1/2$ is present there. This concludes the proof.

There are two remarkable things about eq. (6.4). Firstly, it is an entirely geometrical formula; no colour algebra is required, and its expression is fully determined by the structure of the colour loops. Secondly, such loops are Born-level quantities, whereas one would expect that soft radiation pattern be determined by $(n+1)$-body matrix elements. In fact, there is no contradiction between these two statements: colour-wise, the present case is so tightly constrained (by the Kronecker $\delta$'s in eq. (6.3), and by the structure that leads to eqs. (6.6) and (6.7)) as to give a one-to-one correspondence between Born- and real-emission-level quantities.

### 6.2 Quark-gluon matrix elements

The calculation of the soft limit of interest here is trivial if one exploits the results of sections 5.2 and 6.1. The essence of the procedure of section 5.2 is to write the matrix

[12]A closed colour line is obtained by merging open colour lines, after reversing those that belong to the L-flow: starting from one given such line in the R-flow, one attaches to its end the colour line in the L-flow whose antiquark label is identical to that of the line in the R-flow; the procedure is iterated until the quark label in the colour line of the last L-flow coincides with that of the first line considered in the R-flow.

JHEP11(2021)045

elements for a quark-gluon process in terms of secondary (i.e., quark-only) flows. However, the procedure is iterative, and can be stopped after dealing with any number of gluons. This is not of particular interest when all partons are hard and well separated from each other, but in the present context it is helpful, since one can get rid of all gluons except that which becomes soft. By doing so, one is left with a sum of $q$-quark-antiquark, one-gluon contributions, each of which has a soft limit given by eq. (6.4). By putting all together one obtains

$$\begin{aligned}\mathcal{M}^{(2q;n+1)}_{\rm SOFT}(\gamma',\gamma) &= 2g_S^2\,\widehat{\mathcal{A}}^{(2q;n)}(\gamma')^\star\widehat{\mathcal{A}}^{(2q;n)}(\gamma)\,N^{-\rho(\gamma)-\rho(\gamma')}\\ &\times C_F\sum_{p=0}^{n}\sum_{s_p\in S_p^{(n)}}\frac{(-1)^p}{2^n}\,N^{-p}\,N^{\left|\mathcal{L}(\bar\gamma'_{\cancel{s_p}},\bar\gamma_{\cancel{s_p}})\right|}\\ &\times\sum_{\ell\in\mathcal{L}(\bar\gamma'_{\cancel{s_p}},\bar\gamma_{\cancel{s_p}})}\sum_{k,l\in\ell}^{k<l}(-1)^{\delta_\ell(k,l)}\Big[k,l\Big]\,.\end{aligned}\tag{6.9}$$

Equation (6.9) allows one to sum over flows, by following again the same procedure as in section 5.2. Defining

$$\mathcal{M}^{(2q;n+1)}_{\rm SOFT}=\sum_{\gamma,\gamma'\in\mathcal{F}_{2q;n}}\mathcal{M}^{(2q;n+1)}_{\rm SOFT}(\gamma',\gamma)\tag{6.10}$$

from eqs. (5.46) and (6.9) one obtains

$$\begin{aligned}\mathcal{M}^{(2q;n+1)}_{\rm SOFT} &= 2g_S^2\,C_F\sum_{p=0}^{n}\sum_{s_p\in S_p^{(n)}}\frac{(-1)^p}{2^n}\,N^{-p}\\ &\times\sum_{\bar\gamma,\bar\gamma'\in\mathcal{F}^{(s_p)}_{2q;n-p}}\bar{\mathcal{A}}^{(2q;n)}(\bar\gamma')^\star\,\bar{\mathcal{A}}^{(2q;n)}(\bar\gamma)\,N^{-\rho(\bar\gamma)-\rho(\bar\gamma')}N^{|\mathcal{L}(\bar\gamma',\bar\gamma)|}\\ &\times\sum_{\ell\in\mathcal{L}(\bar\gamma',\bar\gamma)}\sum_{k,l\in\ell}^{k<l}(-1)^{\delta_\ell(k,l)}\Big[k,l\Big]\,.\end{aligned}\tag{6.11}$$

For future use, we denote the sum in the last line of eq. (6.11) as follows:

$$\mathcal{E}(\bar\gamma',\bar\gamma)=\sum_{\ell\in\mathcal{L}(\bar\gamma',\bar\gamma)}\sum_{k,l\in\ell}^{k<l}(-1)^{\delta_\ell(k,l)}\Big[k,l\Big]\,,\tag{6.12}$$

and call it the *radiation pattern* associated with the (secondary) closed flow $(\bar\gamma',\bar\gamma)$.

The similarity between eqs. (6.9) and (6.11) on the one hand, and eq. (6.4) on the other, is remarkable, in view of the fact that, at variance with the case of a single final-state gluon, matrix elements that feature at least two gluons have a more involved colour structure, which prevents one from establishing in a straightforward manner a one-to-one correspondence between Born- and real-emission-level quantities. As is shown here, this can be done by employing secondary flows.

JHEP11(2021)045

Needless to say, eq. (6.11) can be cast in the standard[13] form for soft matrix elements summed over flows (see e.g. eq. (**I**.3.33)), namely

$$\mathcal{M}^{(2q;n+1)}_{\rm SOFT} = \frac{1}{2}\, g_S^2 \sum_{k,l=-2q}^{n} \Big[k,l\Big] \mathcal{M}^{(2q;n)}_{kl}\,, \tag{6.13}$$

where use is made of the colour-linked Borns, $\mathcal{M}^{(2q;n)}_{kl}$, whose definition can be found in eq. (**I**.3.35). These quantities satisfy the completeness relations (see e.g. eq. (3.14) of ref. [32])

$$\sum_{\substack{l\neq k\\ l=-2q}}^{n} \mathcal{M}^{(2q;n)}_{kl} = 2C(\mathcal{I}_k)\mathcal{M}^{(2q;n)}\,, \tag{6.14}$$

which, as is shown in ref. [23] using the language of the present paper, stem from the conservation of colour. In eq. (6.14) by $C(\mathcal{I}_k)$ we have denoted the Casimir factor (equal to $C_F$ for quarks and to $C_A$ for gluons) of a parton with identity $\mathcal{I}_k$.

### 6.3 Gluon-only matrix elements

The case of gluon-only matrix elements can be dealt with as has been done in section 6.2, thanks to the equivalence established in section 5.3 between gluon-only colour flows and suitably constructed quark-gluon ones, eqs. (5.48) and (5.49); in particular, the properties in eqs. (5.55)–(5.57) are crucial. Thus

$$\begin{aligned}\mathcal{M}^{(n+1)}_{\rm SOFT}(\sigma',\sigma) = {}& 2g_S^2\, \widehat{\mathcal{A}}^{(n)}(\sigma')^\star \widehat{\mathcal{A}}^{(n)}(\sigma)\\ & \times C_F \sum_{p=0}^{n} \sum_{s_p\in S_p^{(n)}} \frac{(-1)^p}{2^n}\, N^{-p}\, N^{\left|\mathcal{L}(\bar\sigma'_{qL\,\not{s}_p},\bar\sigma_{qR\,\not{s}_p})\right|}\\ & \times \sum_{\ell\in\mathcal{L}\bar\sigma'_{qL\,\not{s}_p},\bar\sigma_{qR\,\not{s}_p}} \sum_{k,l\in\ell}^{0<k<l} (-1)^{\delta_\ell(k,l)} \Big[k,l\Big]\,. \end{aligned}\tag{6.15}$$

Note that, at variance with what happens in eq. (6.9), the innermost sum on the r.h.s. of eq. (6.15) constrains the indices $k$ and $l$ to be larger than zero, which in turn implies that no quarks or antiquarks will appear in the eikonals. This is an obvious requirement, since the quarks and antiquarks introduced in eqs. (5.48) and (5.49) are unphysical.

With a procedure analogous to that which leads to eq. (5.66), from eq. (6.15) one can also obtain the soft matrix element summed over flows

$$\begin{aligned}\mathcal{M}^{(n+1)}_{\rm SOFT} = {}& \sum_{\sigma,\sigma'\in P'_n} \mathcal{M}^{(n+1)}_{\rm SOFT}(\sigma',\sigma)\\ = {}& \frac{2g_S^2\, C_F}{2^n} \sum_{\bar\sigma,\bar\sigma'\in P'_n} N^{|\mathcal{L}(\bar\sigma',\bar\sigma)|}\, \widehat{\mathcal{A}}^{(n)}(\bar\sigma')^\star\, \widehat{\mathcal{A}}^{(n)}(\bar\sigma)\\ & \times \sum_{\ell\in\mathcal{L}(\bar\sigma',\bar\sigma)} \sum_{k,l\in\ell}^{0<k<l} (-1)^{\delta_\ell(k,l)} \Big[k,l\Big]\,, \end{aligned}\tag{6.16}$$

[13] With only possible differences of notation, eq. (6.13) holds for the quark- and gluon-only cases as well, which we refrain from writing explicitly.

JHEP11(2021)045

which results from significant simplifications that stem from employing the dual Ward identities of eq. (5.63), and eq. (5.64).

### 6.4 General considerations

Equations (6.4), (6.9), and (6.15), or their flow-summed counterparts (eqs. (6.11) and (6.16), the quark-only case being trivially obtained from eq. (6.4)) tell one that processes that feature only quarks at the Born level are essentially maximally complicated as far as soft-radiation patterns are concerned. Gluons are either treated as equivalent to a quark-antiquark pair, or they just decouple completely, consistently with their interpretation as U(1) colourless objects.

These results also suggest a natural generalization of the concept of colour partner we are used to in the context of current PSMCs: given a parton (understood here as either a quark, or an antiquark, or one of the two colour indices of an SU($N$) gluon), its colour partners are all of the partons that belong to the colour loop to which that parton also belongs. This idea indeed encompasses the standard one, which is relevant to leading-$N$ contributions, where $\gamma' = \gamma$ or $\sigma' = \sigma$, and no U(1) gluons in the case of quark-gluon and gluon-only processes. In such a situation, loops are trivial, and it is easy to see that one ends up with quark and antiquarks having one colour partner, and gluons having two of them (one for the colour, and one for the anticolour), as usual. In general, however, a parton can have up to $(n-1)$ colour partners (i.e. up to $2(n-1)$ for a gluon, if its colour and anticolour are simultaneously accounted for), corresponding to a more involved radiation pattern than that relevant to a leading-$N$ picture.

It should be clear that, having chosen a parton, its colour partners are those that enter all of the eikonals that also feature the former one. From the results presented before, one sees that eikonals contribute to the cross section with either sign, and therefore cancellations must be expected. Therefore, in a refinement of the previous paragraph, one is entitled to consider as colour partners only those partons that enter the eikonals that survive algebraic simplifications.

Some such simplifications can be understood in a fairly general manner. Consider in particular the case of a gluon, and its role in colour loops, as detailed in item iv. in section 3. Suppose that the two colour indices of a given gluon, which we denote by $g^\star$, appear in the same loop. It follows that all of the eikonals that feature $g^\star$ will cancel in the physical cross section. This is because those two indices, per item iv., will correspond to one L-element and to one R-element. Any other parton $k$ in the loop will therefore result in two eikonals, both equal to $[k, g^\star]$ but with opposite signs in front — this is because the two distances $\delta_\ell(k, g^\star)$ when computed with $g^\star$ regarded as an L- or an R-element will have opposite parity. A corollary of this is that when a closed colour flow in a quark-gluon process corresponds to a single colour loop, then no eikonal that contains at least one gluon contributes to the cross section.

JHEP11(2021)045

| $(\bar\gamma',\bar\gamma)$ | $p$ | $\rho(\bar\gamma')$ | $\rho(\bar\gamma)$ | $\mathcal{L}(\bar\gamma',\bar\gamma)$ | $\lvert\mathcal{L}(\bar\gamma',\bar\gamma)\rvert$ | Col.fac. |
|---|---|---|---|---|---|---|
| $(\bar\gamma_{0,1},\bar\gamma_{0,1})$ | 0 | 0 | 0 | $\Big\{(-1,\bar1),(\bar1,\bar2),(\bar2,-2)\Big\}$ | 3 | $N^3$ |
| $(\bar\gamma_{0,1},\bar\gamma_{0,2})$ | 0 | 0 | 0 | $\Big\{(-1,\bar2,\bar1,-2,\bar2,\bar1)\Big\}$ | 1 | $N$ |
| $(\bar\gamma_{0,2},\bar\gamma_{0,2})$ | 0 | 0 | 0 | $\Big\{(-1,\bar2),(\bar2,\bar1),(\bar1,-2)\Big\}$ | 3 | $N^3$ |
| $(\bar\gamma_{1,1},\bar\gamma_{1,1})$ | 1 | 0 | 0 | $\Big\{(-1,\bar2),(\bar2,-2)\Big\}$ | 2 | $-N$ |
| $(\bar\gamma_{1,2},\bar\gamma_{1,2})$ | 1 | 0 | 0 | $\Big\{(-1,\bar1),(\bar1,-2)\Big\}$ | 2 | $-N$ |
| $(\bar\gamma_{2,1},\bar\gamma_{2,1})$ | 2 | 0 | 0 | $\Big\{(-1,-2)\Big\}$ | 1 | $N^{-1}$ |

**Table 1**. Quantities relevant to eq. (6.11) for $\bar qq\to gg$, for each non-trivial closed flow. See the text for details.

### 6.5 Examples

#### 6.5.1 $\bar qq\to gg$

The set of primary flows was given in eq. (3.4). The sets of indices of U(1) gluons, eq. (5.10), are

$$S_0^{(2)}=\Big\{\{\}\Big\}\equiv\Big\{s_0\Big\},\quad S_1^{(2)}=\Big\{\{1\},\{2\}\Big\}\equiv\Big\{s_{1,1},s_{1,2}\Big\},\quad S_2^{(2)}=\Big\{\{1,2\}\Big\}\equiv\Big\{s_2\Big\}. \tag{6.17}$$

The sets of secondary flows, eq. (5.34), are

$$\mathcal{F}_{2;2}^{(s_0)}=\{(-1;\bar1,\bar2;-2)\,,(-1;\bar2,\bar1;-2)\}\equiv\{\bar\gamma_{0,1},\bar\gamma_{0,2}\}\ , \tag{6.18}$$
$$\mathcal{F}_{2;1}^{(s_{1,1})}=\{(-1;\bar2;-2)\}\equiv\{\bar\gamma_{1,1}\}\ , \tag{6.19}$$
$$\mathcal{F}_{2;1}^{(s_{1,2})}=\{(-1;\bar1;-2)\}\equiv\{\bar\gamma_{1,2}\}\ , \tag{6.20}$$
$$\mathcal{F}_{2;0}^{(s_2)}=\{(-1;-2)\}\equiv\{\bar\gamma_{2,1}\}\ . \tag{6.21}$$

Table 1 reports the various quantities that appear in eq. (6.11), for each non-trivial secondary closed flow — number of U(1) gluons ($p$, first column), $\rho$ parameters for the L- and R-flows (second and third columns), set of colour loops (fourth column, eq. (3.1)), number of colour loops (fifth column), and colour factors (sixth column; these are defined as the products of all the $N$ monomials in eq. (6.11), times the factor $(-)^p$).

The radiation patterns (see eq. (6.12)) associated with the secondary closed flows of table 1 read as follows:

$$\mathcal{E}\left(\bar\gamma_{0,1},\bar\gamma_{0,1}\right)=\Big[-1,\bar1\Big]+\Big[\bar1,\bar2\Big]+\Big[\bar2,-2\Big]\,, \tag{6.22}$$
$$\mathcal{E}\left(\bar\gamma_{0,1},\bar\gamma_{0,2}\right)=\Big[-1,-2\Big]\,, \tag{6.23}$$
$$\mathcal{E}\left(\bar\gamma_{0,2},\bar\gamma_{0,2}\right)=\Big[-1,\bar2\Big]+\Big[\bar2,\bar1\Big]+\Big[\bar1,-2\Big]\,, \tag{6.24}$$

JHEP11(2021)045

$$\mathcal{E}\left(\bar{\gamma}_{1,1},\bar{\gamma}_{1,1}\right) = \left[-1,\bar{2}\right] + \left[\bar{2},-2\right], \tag{6.25}$$

$$\mathcal{E}\left(\bar{\gamma}_{1,2},\bar{\gamma}_{1,2}\right) = \left[-1,\bar{1}\right] + \left[\bar{1},-2\right], \tag{6.26}$$

$$\mathcal{E}\left(\bar{\gamma}_{2,1},\bar{\gamma}_{2,1}\right) = \left[-1,-2\right]. \tag{6.27}$$

Notice that (6.23) illustrates the fact, mentioned in section 6.4, that gluons appearing twice in any loop do not contribute to the associated radiation pattern. It trivially follows from eq. (3.4) that there are two independent dual amplitudes

$$\widehat{\mathcal{A}}^{(2;2)}(\gamma_1)\,, \qquad \widehat{\mathcal{A}}^{(2;2)}(\gamma_2)\,. \tag{6.28}$$

Thus, the amplitudes associated with secondary flows are the following (from eq. (5.45), and the definition of the $I_+$ operator, eq. (5.36)):

$$\bar{\mathcal{A}}^{(2;2)}\left(\bar{\gamma}_{0,1}\right) = \widehat{\mathcal{A}}^{(2;2)}\left(\gamma_1\right)\,, \tag{6.29}$$

$$\bar{\mathcal{A}}^{(2;2)}\left(\bar{\gamma}_{0,2}\right) = \widehat{\mathcal{A}}^{(2;2)}\left(\gamma_2\right)\,, \tag{6.30}$$

$$\bar{\mathcal{A}}^{(2;2)}\left(\bar{\gamma}_{1,1}\right) = \widehat{\mathcal{A}}^{(2;2)}\left(\gamma_1\right) + \widehat{\mathcal{A}}^{(2;2)}\left(\gamma_2\right)\,, \tag{6.31}$$

$$\bar{\mathcal{A}}^{(2;2)}\left(\bar{\gamma}_{1,2}\right) = \widehat{\mathcal{A}}^{(2;2)}\left(\gamma_1\right) + \widehat{\mathcal{A}}^{(2;2)}\left(\gamma_2\right)\,, \tag{6.32}$$

$$\bar{\mathcal{A}}^{(2;2)}\left(\bar{\gamma}_{2,1}\right) = \widehat{\mathcal{A}}^{(2;2)}\left(\gamma_1\right) + \widehat{\mathcal{A}}^{(2;2)}\left(\gamma_2\right)\,. \tag{6.33}$$

This leads to four quantities that enter eqs. (5.46) (tree level) and (6.11) (soft limit). Note that amplitudes associated with different sets of U(1) gluons do not interfere with each other. By direct computation, defining $s = (p_{-1} + p_{-2})^2$, $t = (p_{-1} + p_1)^2$, $u = (p_{-1} + p_2)^2$,

$$M_1 \equiv \bar{\mathcal{A}}^{(2;2)}(\bar{\gamma}_{0,1})^\star\, \bar{\mathcal{A}}^{(2;2)}(\bar{\gamma}_{0,1}) = 8 g_S^4 \left(\frac{u}{t} - 2\,\frac{u^2}{s^2}\right)\,, \tag{6.34}$$

$$M_2 \equiv 2\Re\left(\bar{\mathcal{A}}^{(2;2)}(\bar{\gamma}_{0,1})^\star\, \bar{\mathcal{A}}^{(2;2)}(\bar{\gamma}_{0,2})\right) = 16\, g_S^4 \frac{t^2 + u^2}{s^2}\,, \tag{6.35}$$

$$M_3 \equiv \bar{\mathcal{A}}^{(2;2)}(\bar{\gamma}_{0,2})^\star\, \bar{\mathcal{A}}^{(2;2)}(\bar{\gamma}_{0,2}) = 8 g_S^4 \left(\frac{t}{u} - 2\,\frac{t^2}{s^2}\right)\,, \tag{6.36}$$

$$\begin{aligned} M_4 &\equiv \left(\widehat{\mathcal{A}}^{(2;2)}(\gamma_1) + \widehat{\mathcal{A}}^{(2;2)}(\gamma_2)\right)^\star \left(\widehat{\mathcal{A}}^{(2;2)}(\gamma_1) + \widehat{\mathcal{A}}^{(2;2)}(\gamma_2)\right) \\ &= M_1 + M_2 + M_3 = 8 g_S^4 \left(\frac{t}{u} + \frac{u}{t}\right)\,. \end{aligned} \tag{6.37}$$

With this, eq. (5.46) leads to (note that $\rho(\bar{\gamma}_\alpha) = 0$ for any $\alpha$)

$$4\mathcal{M}^{(2;2)} = N^3 \left(M_1 + M_3\right) + N M_2 - \frac{2}{N}\, N^2\, M_4 + \frac{1}{N^2}\, N\, M_4\,, \tag{6.38}$$

with the first two, the third, and the fourth term on the r.h.s. stemming from the contributions associated with zero, one, and two U(1) gluons, respectively. Likewise, from

JHEP11(2021)045

eq. (6.11) we obtain

$$\frac{2\mathcal{M}^{(2;3)}_{\text{SOFT}}}{g_S^2 C_F} = N^3 \mathcal{E}\left(\bar\gamma_{0,1}, \bar\gamma_{0,1}\right) M_1 + N^3 \mathcal{E}\left(\bar\gamma_{0,2}, \bar\gamma_{0,2}\right) M_3 + N \mathcal{E}\left(\bar\gamma_{0,1}, \bar\gamma_{0,2}\right) M_2$$
$$- \frac{1}{N} N^2 \left(\mathcal{E}\left(\bar\gamma_{1,1}, \bar\gamma_{1,1}\right) + \mathcal{E}\left(\bar\gamma_{1,2}, \bar\gamma_{1,2}\right)\right) M_4$$
$$+ \frac{1}{N^2} N \mathcal{E}\left(\bar\gamma_{2,1}, \bar\gamma_{2,1}\right) M_4 \,, \tag{6.39}$$

with the first, second, and third line on the r.h.s. stemming from the contributions associated with zero, one, and two U(1) gluons, respectively.

In terms of the colour-linked Borns introduced in eq. (6.13), in this case denoted by $\mathcal{M}^{(2;2)}_{kl}$, we have

$$\mathcal{M}^{(2;3)}_{\text{SOFT}} = \frac{1}{2} g_S^2 \sum_{k,l=-2}^{2} \Big[k, l\Big] \mathcal{M}^{(2;2)}_{kl} \,, \tag{6.40}$$

and keeping in mind that $\mathcal{M}^{(2;2)}_{kl} = \mathcal{M}^{(2;2)}_{lk}$, we obtain

$$\mathcal{M}^{(2;2)}_{-2-1} = \frac{g_S^4 C_F}{32} \frac{1}{N} \left(M_2 + \frac{1}{N^2} M_4\right) \,, \tag{6.41}$$

$$\mathcal{M}^{(2;2)}_{-21} = \frac{g_S^4 C_F}{32} \left(N M_3 - \frac{1}{N} M_4\right) \,, \tag{6.42}$$

$$\mathcal{M}^{(2;2)}_{-22} = \frac{g_S^4 C_F}{32} \left(N M_1 - \frac{1}{N} M_4\right) \,, \tag{6.43}$$

$$\mathcal{M}^{(2;2)}_{-11} = \frac{g_S^4 C_F}{32} \left(N M_1 - \frac{1}{N} M_4\right) \,, \tag{6.44}$$

$$\mathcal{M}^{(2;2)}_{-12} = \frac{g_S^4 C_F}{32} \left(N M_3 - \frac{1}{N} M_4\right) \,, \tag{6.45}$$

$$\mathcal{M}^{(2;2)}_{12} = \frac{g_S^4 C_F}{32} N \Big(M_1 + M_3\Big) \,. \tag{6.46}$$

As expected, these expressions satisfy the completeness relations (6.14)

$$\sum_{\substack{l \neq k \\ l=-2}}^{2} \mathcal{M}^{(2;2)}_{kl} = 2C(\mathcal{I}_k) \mathcal{M}^{(2;2)} \,. \tag{6.47}$$

### 6.5.2 $\bar q q \to q' \bar q' g$

The process is $\bar q_{-1}\, q_{-3} \to q'_{-2} \bar q'_{-4} g_1$, with $q = 2$ and $n = 1$. The primary flows are

$$\gamma_1 = (-1; 1; -3)\,(-2; -4) \,, \tag{6.48}$$

$$\gamma_2 = (-1; 1; -4)\,(-2; -3) \,, \tag{6.49}$$

$$\gamma_3 = (-2; 1; -3)\,(-1; -4) \,, \tag{6.50}$$

$$\gamma_4 = (-2; 1; -4)\,(-1; -3) \,. \tag{6.51}$$

JHEP11(2021)045

| $(\bar\gamma', \bar\gamma)$ | $p$ | $\rho(\bar\gamma')$ | $\rho(\bar\gamma)$ | $\mathcal{L}(\bar\gamma', \bar\gamma)$ | $\lvert\mathcal{L}(\bar\gamma', \bar\gamma)\rvert$ | Col.fac. |
|---|---|---|---|---|---|---|
| $(\bar\gamma_{0,1}, \bar\gamma_{0,1})$ | 0 | 1 | 1 | $\{(-1, \bar 1), (\bar 1, -3), (-2, -4)\}$ | 3 | $N$ |
| $(\bar\gamma_{0,1}, \bar\gamma_{0,2})$ | 0 | 1 | 0 | $\{(-1, \bar 1), (\bar 1, -4, -2, -3)\}$ | 2 | $N$ |
| $(\bar\gamma_{0,1}, \bar\gamma_{0,3})$ | 0 | 1 | 0 | $\{(-2, \bar 1, -1, -4), (\bar 1, -3)\}$ | 2 | $N$ |
| $(\bar\gamma_{0,1}, \bar\gamma_{0,4})$ | 0 | 1 | 1 | $\{(-2, \bar 1, -1, -3, \bar 1, -4)\}$ | 1 | $N^{-1}$ |
| $(\bar\gamma_{0,2}, \bar\gamma_{0,2})$ | 0 | 0 | 0 | $\{(-1, \bar 1), (\bar 1, -4), (-2, -3)\}$ | 3 | $N^3$ |
| $(\bar\gamma_{0,2}, \bar\gamma_{0,3})$ | 0 | 0 | 0 | $\{(-2, \bar 1, -1, -4, \bar 1, -3)\}$ | 1 | $N$ |
| $(\bar\gamma_{0,2}, \bar\gamma_{0,4})$ | 0 | 0 | 1 | $\{(-2, \bar 1, -1, -3), (\bar 1, -4)\}$ | 2 | $N$ |
| $(\bar\gamma_{0,3}, \bar\gamma_{0,3})$ | 0 | 0 | 0 | $\{(-2, \bar 1), (\bar 1, -3), (-1, -4)\}$ | 3 | $N^3$ |
| $(\bar\gamma_{0,3}, \bar\gamma_{0,4})$ | 0 | 0 | 1 | $\{(-2, \bar 1), (\bar 1, -4, -1, -3)\}$ | 2 | $N$ |
| $(\bar\gamma_{0,4}, \bar\gamma_{0,4})$ | 0 | 1 | 1 | $\{(-2, \bar 1), (\bar 1, -4), (-1, -3)\}$ | 3 | $N$ |
| $(\bar\gamma_{1,1}, \bar\gamma_{1,1})$ | 1 | 1 | 1 | $\{(-1, -3), (-2, -4)\}$ | 2 | $-N^{-1}$ |
| $(\bar\gamma_{1,1}, \bar\gamma_{1,2})$ | 1 | 1 | 0 | $\{(-1, -4, -2, -3)\}$ | 1 | $-N^{-1}$ |
| $(\bar\gamma_{1,2}, \bar\gamma_{1,2})$ | 1 | 0 | 0 | $\{(-1, -4), (-2, -3)\}$ | 2 | $-N$ |

**Table 2**. As in table 1, for $\bar q q \to q' \bar q' g$.

The secondary flows are

$$\bar\gamma_{0,1} = (-1; \bar 1; -3)\,(-2; -4)\ , \tag{6.52}$$
$$\bar\gamma_{0,2} = (-1; \bar 1; -4)\,(-2; -3)\ , \tag{6.53}$$
$$\bar\gamma_{0,3} = (-2; \bar 1; -3)\,(-1; -4)\ , \tag{6.54}$$
$$\bar\gamma_{0,4} = (-2; \bar 1; -4)\,(-1; -3)\ , \tag{6.55}$$
$$\bar\gamma_{1,1} = (-1; -3)\,(-2; -4)\ , \tag{6.56}$$
$$\bar\gamma_{1,2} = (-1; -4)\,(-2; -3)\ . \tag{6.57}$$

The sets of associated colour loops are given in table 2, together with the computation of their colour factors according to eq. (6.11).

Defining

$$S_{ij} \equiv \frac{s_{i,j}}{s_{i,1}\, s_{j,1}}\ , \tag{6.58}$$

where $s_{i,j} = (p_i + p_j)^2$, and

$$R = 8 g_S^4 \frac{s^2_{-1,-2} + s^2_{-1,-4} + s^2_{-2,-3} + s^2_{-3,-4}}{s_{-1,-3}\, s_{-2,-4}}\ , \tag{6.59}$$

JHEP11(2021)045

we find by explicit calculation, summing over helicities,

$$M_1 = \widehat{\mathcal{A}}^{(4;1)}(\gamma_1)^\star \widehat{\mathcal{A}}^{(4;1)}(\gamma_1) = S_{-1,-3}\, R\,, \tag{6.60}$$

$$M_2 = 2\Re\left(\widehat{\mathcal{A}}^{(4;1)}(\gamma_1)^\star \widehat{\mathcal{A}}^{(4;1)}(\gamma_2)\right) = (S_{-3,-4} - S_{-1,-3} - S_{-1,-4})\, R\,, \tag{6.61}$$

$$M_3 = 2\Re\left(\widehat{\mathcal{A}}^{(4;1)}(\gamma_1)^\star \widehat{\mathcal{A}}^{(4;1)}(\gamma_3)\right) = (S_{-1,-2} - S_{-1,-3} - S_{-2,-3})\, R\,, \tag{6.62}$$

$$M_4 = 2\Re\left(\widehat{\mathcal{A}}^{(4;1)}(\gamma_1)^\star \widehat{\mathcal{A}}^{(4;1)}(\gamma_4)\right) = (S_{-1,-4} + S_{-2,-3} - S_{-1,-2} - S_{-3,-4})\, R\,, \tag{6.63}$$

$$M_5 = \widehat{\mathcal{A}}^{(4;1)}(\gamma_2)^\star \widehat{\mathcal{A}}^{(4;1)}(\gamma_2) = S_{-1,-4}\, R\,, \tag{6.64}$$

$$M_6 = 2\Re\left(\widehat{\mathcal{A}}^{(4;1)}(\gamma_2)^\star \widehat{\mathcal{A}}^{(4;1)}(\gamma_3)\right) = (S_{-1,-3} + S_{-2,-4} - S_{-1,-2} - S_{-3,-4})\, R\,, \tag{6.65}$$

$$M_7 = 2\Re\left(\widehat{\mathcal{A}}^{(4;1)}(\gamma_2)^\star \widehat{\mathcal{A}}^{(4;1)}(\gamma_4)\right) = (S_{-1,-2} - S_{-1,-4} - S_{-2,-4})\, R\,, \tag{6.66}$$

$$M_8 = \widehat{\mathcal{A}}^{(4;1)}(\gamma_3)^\star \widehat{\mathcal{A}}^{(4;1)}(\gamma_3) = S_{-2,-3}\, R\,, \tag{6.67}$$

$$M_9 = 2\Re\left(\widehat{\mathcal{A}}^{(4;1)}(\gamma_3)^\star \widehat{\mathcal{A}}^{(4;1)}(\gamma_4)\right) = (S_{-3,-4} - S_{-2,-3} - S_{-2,-4})\, R\,, \tag{6.68}$$

$$M_{10} = \widehat{\mathcal{A}}^{(4;1)}(\gamma_4)^\star \widehat{\mathcal{A}}^{(4;1)}(\gamma_4) = S_{-2,-4}\, R\,. \tag{6.69}$$

Note that only six of these are independent, owing to the property of the dual amplitudes

$$\sum_{i=1}^{4} \widehat{\mathcal{A}}^{(4;1)}(\gamma_i) = 0\,. \tag{6.70}$$

The products of secondary flow amplitudes follow from eq. (5.45), which gives in this case

$$\bar{\mathcal{A}}^{(4;1)}(\bar{\gamma}_{0,i}) = \widehat{\mathcal{A}}^{(4;1)}(\gamma_i)\,, \qquad 1 \le i \le 4\,, \tag{6.71}$$

$$\bar{\mathcal{A}}^{(4;1)}(\bar{\gamma}_{1,1}) = \widehat{\mathcal{A}}^{(4;1)}(\gamma_1) + \widehat{\mathcal{A}}^{(4;1)}(\gamma_4)\,, \tag{6.72}$$

$$\bar{\mathcal{A}}^{(4;1)}(\bar{\gamma}_{1,2}) = \widehat{\mathcal{A}}^{(4;1)}(\gamma_2) + \widehat{\mathcal{A}}^{(4;1)}(\gamma_3)\,. \tag{6.73}$$

The radiation patterns associated with the secondary closed flows are

$$\mathcal{E}(\bar{\gamma}_{0,1}, \bar{\gamma}_{0,1}) = \Big[-1, \bar{1}\Big] + \Big[\bar{1}, -3\Big] + \Big[-2, -4\Big]\,, \tag{6.74}$$

$$\begin{aligned}\mathcal{E}(\bar{\gamma}_{0,1}, \bar{\gamma}_{0,2}) = {} & \Big[-1, \bar{1}\Big] + \Big[\bar{1}, -4\Big] - \Big[\bar{1}, -2\Big] + \Big[\bar{1}, -3\Big] + \Big[-4, -2\Big] \\ & - \Big[-4, -3\Big] + \Big[-2, -3\Big]\,,\end{aligned} \tag{6.75}$$

$$\begin{aligned}\mathcal{E}(\bar{\gamma}_{0,1}, \bar{\gamma}_{0,3}) = {} & \Big[-2, \bar{1}\Big] - \Big[-2, -1\Big] + \Big[-2, -4\Big] + \Big[\bar{1}, -1\Big] - \Big[\bar{1}, -4\Big] \\ & + \Big[-1, -4\Big] + \Big[\bar{1}, -3\Big]\,,\end{aligned} \tag{6.76}$$

$$\begin{aligned}\mathcal{E}(\bar{\gamma}_{0,1}, \bar{\gamma}_{0,4}) = {} & \Big[-1, -3\Big] + \Big[-1, -4\Big] - \Big[-2, -1\Big] + \Big[-2, -3\Big] \\ & + \Big[-2, -4\Big] - \Big[-3, -4\Big]\,,\end{aligned} \tag{6.77}$$

JHEP11(2021)045

$$\mathcal{E}(\bar{\gamma}_{0,2},\bar{\gamma}_{0,2}) = \left[-1,\bar{1}\right] + \left[\bar{1},-4\right] + \left[-2,-3\right], \tag{6.78}$$

$$\begin{aligned}\mathcal{E}(\bar{\gamma}_{0,2},\bar{\gamma}_{0,3}) = &-\left[-2,-1\right] + \left[-2,-4\right] + \left[-2,-3\right] + \left[-1,-4\right] \\ &+\left[-1,-3\right] - \left[-4,-3\right],\end{aligned} \tag{6.79}$$

$$\begin{aligned}\mathcal{E}(\bar{\gamma}_{0,2},\bar{\gamma}_{0,4}) = &\left[-2,\bar{1}\right] - \left[-2,-1\right] + \left[-2,-3\right] + \left[\bar{1},-1\right] - \left[\bar{1},-3\right] \\ &+\left[-1,-3\right] + \left[\bar{1},-4\right],\end{aligned} \tag{6.80}$$

$$\mathcal{E}(\bar{\gamma}_{0,3},\bar{\gamma}_{0,3}) = \left[-2,\bar{1}\right] + \left[\bar{1},-3\right] + \left[-1,-4\right], \tag{6.81}$$

$$\begin{aligned}\mathcal{E}(\bar{\gamma}_{0,3},\bar{\gamma}_{0,4}) = &\left[-2,\bar{1}\right] + \left[\bar{1},-4\right] - \left[\bar{1},-1\right] + \left[\bar{1},-3\right] + \left[-4,-1\right] \\ &-\left[-4,-3\right] + \left[-1,-3\right],\end{aligned} \tag{6.82}$$

$$\mathcal{E}(\bar{\gamma}_{0,4},\bar{\gamma}_{0,4}) = \left[-2,\bar{1}\right] + \left[\bar{1},-4\right] + \left[-1,-3\right], \tag{6.83}$$

$$\mathcal{E}(\bar{\gamma}_{1,1},\bar{\gamma}_{1,1}) = \left[-1,-3\right] + \left[-2,-4\right], \tag{6.84}$$

$$\begin{aligned}\mathcal{E}(\bar{\gamma}_{1,1},\bar{\gamma}_{1,2}) = &\left[-1,-4\right] - \left[-1,-2\right] + \left[-1,-3\right] + \left[-4,-2\right] \\ &-\left[-4,-3\right] + \left[-2,-3\right],\end{aligned} \tag{6.85}$$

$$\mathcal{E}(\bar{\gamma}_{1,2},\bar{\gamma}_{1,2}) = \left[-1,-4\right] + \left[-2,-3\right]. \tag{6.86}$$

Here again, eqs. (6.77) and (6.79) reflect the fact that a gluon appearing twice in the same loop does not contribute to the associated radiation pattern. Using eqs. (6.60)–(6.69), eqs. (6.71)–(6.73), and the $\rho$ parameters and colour factors of table 2, from eq. (5.46) one obtains

$$\mathcal{M}^{(4;1)} = C_F\left[N^2\left(M_5+M_8\right)+M_1+M_2+M_3+M_7+M_9+M_{10}\right], \tag{6.87}$$

for the tree-level matrix element of the current process. Furthermore, by writing the flow-summed soft limit in the standard form as was done in eq. (6.13), namely

$$\mathcal{M}^{(4;2)}_{\rm SOFT} = \frac{1}{2}\,g_S^2\sum_{k,l=-4}^{1}\left[k,l\right]\mathcal{M}^{(4;1)}_{kl}, \tag{6.88}$$

and using eqs. (6.74)–(6.86) in eq. (6.11), we arrive at the following expressions for the

JHEP11(2021)045

colour-linked Borns:

$$\mathcal{M}^{(4;1)}_{-4,-3} = C_F\Big[-N\Big(M_2+M_6+M_9\Big)+\frac{1}{N}\Big(M_2+M_3-M_4+M_7+M_9\Big)\Big]\,, \tag{6.89}$$

$$\mathcal{M}^{(4;1)}_{-4,-2} = C_F\Big[N\Big(M_1+M_2+M_3+M_6\Big) \tag{6.90}$$

$$-\frac{1}{N}\Big(M_1+M_2+M_3+M_7+M_9+M_{10}\Big)\Big]\,, \tag{6.91}$$

$$\mathcal{M}^{(4;1)}_{-4,-1} = C_F\Big[N^3M_8+N\Big(M_3-M_5-M_8+M_9\Big) \tag{6.92}$$

$$-\frac{1}{N}\Big(M_2+M_3-M_4+M_7+M_9\Big)\Big]\,, \tag{6.93}$$

$$\mathcal{M}^{(4;1)}_{-4,1} = C_FN\Big[N^2M_5+M_2-M_3+M_7+M_9+M_{10}\Big]\,, \tag{6.94}$$

$$\mathcal{M}^{(4;1)}_{-3,-2} = C_F\Big[N^3M_5+N\Big(M_2-M_5+M_7-M_8\Big) \tag{6.95}$$

$$-\frac{1}{N}\Big(M_2+M_3-M_4+M_7+M_9\Big)\Big]\,, \tag{6.96}$$

$$\mathcal{M}^{(4;1)}_{-3,-1} = C_F\Big[N\Big(M_6+M_7+M_9+M_{10}\Big) \tag{6.97}$$

$$-\frac{1}{N}\Big(M_1+M_2+M_3+M_7+M_9+M_{10}\Big)\Big]\,, \tag{6.98}$$

$$\mathcal{M}^{(4;1)}_{-3,1} = C_FN\Big[N^2M_8+M_1+M_2+M_3-M_7+M_9\Big]\,, \tag{6.99}$$

$$\mathcal{M}^{(4;1)}_{-2,-1} = C_F\Big[-N\Big(M_3+M_6+M_7\Big)+\frac{1}{N}\Big(M_2+M_3-M_4+M_7+M_9\Big)\Big]\,, \tag{6.100}$$

$$\mathcal{M}^{(4;1)}_{-2,1} = C_FN\Big[N^2M_8-M_2+M_3+M_7+M_9+M_{10}\Big]\,, \tag{6.101}$$

$$\mathcal{M}^{(4;1)}_{-1,1} = C_FN\Big[N^2M_5+M_1+M_2+M_3+M_7-M_9\Big]\,. \tag{6.102}$$

Again, one can verify that these satisfy the completeness relations (6.14)

$$\sum_{\substack{l\neq k\\ l=-4}}^{1}\mathcal{M}^{(4;1)}_{kl} = 2C(\mathcal{I}_k)\mathcal{M}^{(4;1)}\,. \tag{6.103}$$

## 7 Monte Carlo simulation

In a parton shower Monte Carlo, each parton of a primary hard subprocess has the potential to generate a shower, with initial conditions to be determined by the kinematics and colour structure of the hard process. In the case of a dipole shower, each parton is paired with another to form a dipole which sets the initial scale of the shower according to its invariant mass. In a single-parton shower, the partner is again involved in setting the initial scale of evolution. For example, in an angular-ordered shower it determines the maximum angle of emission in the frame of shower evolution. In the leading-colour approximation, the parton and its partner form a colour singlet, so that the end products of the shower from a dipole, or from the angular-ordered showers of a parton and its partner, can hadronize without involving the products of showers initiated by other hard partons.

In order to reproduce the leading-order soft gluon emission pattern from an $m$-parton hard process, the dipoles or parton pairs have to be associated with eikonal terms in the

JHEP11(2021)045

soft limit of the $(m+1)$-parton process. In the leading-colour approximation, considering all partons as outgoing, every quark can be associated with an antiquark or the anticolour of a gluon, every antiquark with a quark or the colour of a gluon, and the remaining colours and anticolours of gluons with other gluons.

As we have seen, at subleading colour there are eikonal terms that pair quarks with quarks and antiquarks with antiquarks. There may also be a term where a given gluon is paired with a quark and a term where it is paired with another quark, and similarly for pairing with two antiquarks. We can still associate dipoles or parton pairs with such terms, and use them to generate showers, but the final products cannot form colour singlets without communicating with other showers. However, since colour is conserved overall, it is always possible to hadronize after some colour reconnection between showers.

In this section we discuss some strategies that could be adopted to include subleading colour contributions from the hard subprocess. Which of these strategies may prove to be the most practical or efficient probably depends on the process under consideration and remains for further investigation. A related but separate issue is the inclusion of subleading colour contributions inside parton showers, studied for example in refs. [1–6]. We envisage that the use of colour flows in matrix elements may prove useful in this case as well, since it has the advantage that it leads to an easy accounting of the number of colours, and thus renders it straightforward to see which contributions need to be taken into account to achieve any desired level of accuracy w.r.t. $N$.

### 7.1 Single-parton showers

In a single-parton shower, the eikonal pairings are used to set the evolution scales but the showering of partons is otherwise independent. To achieve this the dipoles associated with eikonal terms have to be split in some way between the two partons involved. As an example, we consider here the splitting that leads to angular-ordered shower evolution. For simplicity we adopt the original formulation used in the Herwig6 event generator [33], rather than the more sophisticated treatments in Herwig+ + [34] and Herwig7 [35, 36], which are equivalent for our purposes.

#### 7.1.1 Angular-ordered shower

For an angular-ordered parton shower, one makes the following substitution for the eikonal (6.2):

$$\left[k,l\right] = \frac{1}{\omega^2}\frac{\xi_{kl}}{\xi_k\xi_l} \quad \Longrightarrow \quad \frac{1}{\omega^2}\left[\frac{\Theta(\xi_{kl}-\xi_k)}{\xi_k}+\frac{\Theta(\xi_{kl}-\xi_l)}{\xi_l}\right], \tag{7.1}$$

where $\omega$ is the energy of the emitted soft gluon, $\xi_{kl} = 1-\cos\theta_{kl}$ where $\theta_{kl}$ is the angle between the three-momenta of partons $k$ and $l$; $\xi_k = 1-\cos\theta_k$ where $\theta_k$ is the angle between the three-momenta of the soft gluon and parton $k$, and similarly for $\xi_l$. This approximation follows from separating the collinear singularities of the eikonal and azimuthally averaging each term. The averaging destroys Lorentz invariance and so one has to perform it in the same frame as that used by the parton shower. The subsequent evolution of each shower is then ordered with respect to the relevant angular variable, $\xi_k$ or $\xi_l$. This ordering takes

JHEP11(2021)045

into account the coherence of emission from the ensemble of partons in the shower at angles smaller than the current value.

Making the substitution (7.1) in eq. (6.13), we have

$$\mathcal{M}^{(2q;n+1)}_{\rm SOFT} \quad\Longrightarrow\quad \frac{g_S^2}{\omega^2}\sum_{k,l}\mathcal{M}^{(2q;n)}_{kl}\frac{\Theta(\xi_{kl}-\xi_k)}{\xi_k}\,. \tag{7.2}$$

For any given $k$, using the phase-space element for soft gluon emission,

$$\frac{d^3p}{2(2\pi)^3\omega} = \frac{\omega}{2(2\pi)^3} d\omega\, d\xi_k d\phi_k\,, \tag{7.3}$$

and integrating over $\phi_k$, the azimuthal angle of emission with respect to parton $k$, the differential distribution of soft gluon emission becomes

$$\frac{\alpha_S}{2\pi}\sum_{k,l}\mathcal{M}^{(2q;n)}_{kl}\,\Theta(\xi_{kl}-\xi_k)\frac{d\omega}{\omega}\frac{d\xi_k}{\xi_k}. \tag{7.4}$$

The emission of non-soft collinear gluons can be included by replacing $d\omega/\omega$ by $P(z)\,dz$, where $P(z)$ is the appropriate collinear splitting function of $z = 1-\omega/p_k^0$.

In a leading-colour treatment [37], one first generates a kinematic configuration of the Born process using its full matrix element and then chooses among the leading-$N$ colour flows (if there are more than one) according to their relative contributions. In the radiation pattern of a leading-$N$ flow, all eikonal terms have coefficient $+1$, each quark or antiquark appears in only one eikonal term, and each gluon appears in two. Therefore, if $k$ is a quark or antiquark, there is a unique colour partner $l$ in eq. (7.4), which specifies the angular region of radiation from $k$, $\xi_k < \xi_{kl}$. On the other hand, if $k$ is a gluon it has two partners, say $l$ and $m$ such that $\xi_{km} < \xi_{kl}$, and it radiates with half intensity when $\xi_{km} < \xi_k < \xi_{kl}$ and full intensity when $\xi_k < \xi_{km}$.

These radiation patterns, together with subsequent multiple emissions, can be generated by a parton showering process controlled by a Sudakov form factor

$$\Delta_k(\xi_k) = \exp\left[-C(\mathcal{I}_k)\frac{\alpha_S}{2\pi}\int_{\xi_0}^{\xi_k}\frac{d\xi}{\xi}\int P(z)\,dz\right], \tag{7.5}$$

where $C(\mathcal{I}_k)$ is the relevant Casimir factor (see eq. (6.14)) and $\xi_0$ is a cutoff below which emissions are not resolved. When parton $k$ has a single colour partner $l$, the probability of no emission between the maximum angular scale $\xi_{kl}$ and some lower scale $\xi_k$ is given by $\Delta_k(\xi_{kl})/\Delta_k(\xi_k)$. In the above case of a gluon $k$ with partners $l$ and $m$ such that $\xi_{kl} > \xi_{km}$, the probability of no emission between $\xi_{kl}$ and $\xi_k > \xi_{km}$ is $\sqrt{\Delta_k(\xi_{kl})/\Delta_k(\xi_k)}$ while that between $\xi_{km}$ and $\xi_k < \xi_{km}$ is $\Delta_k(\xi_{km})/\Delta_k(\xi_k)$. In other words, the leading-colour no-emission probability for a given colour flow is controlled by

$$[\Delta_k(\xi_k)]^{p_{\rm LC}(\xi_k)} \tag{7.6}$$

where

$$p_{\rm LC}(\xi_k) = \frac{\sum_l \Theta(\xi_{kl}-\xi_k)}{\sum_l 1}, \tag{7.7}$$

JHEP11(2021)045

the sums being over all colour partners $l$ of $k$ in the chosen leading-$N$ colour flow.

Equations (5.46) and (6.11) suggest an obvious extension of (7.7) to include subleading colour. After selecting a closed flow $(\bar\gamma', \bar\gamma)$ according to its contribution to the Born matrix element in (5.46), the evolution of parton $k$ in that flow is controlled by

$$[\Delta_k(\xi_k)]^{p_{(\bar\gamma',\bar\gamma)}(\xi_k)} \tag{7.8}$$

where

$$p_{(\bar\gamma',\bar\gamma)}(\xi_k) = \frac{\sum_{\ell\in\mathcal{L}(\bar\gamma',\bar\gamma)} \sum_l (-1)^{\delta_\ell(k,l)} \Theta(\xi_{kl} - \xi_k)}{\sum_{\ell\in\mathcal{L}(\bar\gamma',\bar\gamma)} \sum_l (-1)^{\delta_\ell(k,l)}}. \tag{7.9}$$

Consider for example the process $\bar q q \to gg$. We first choose one of the six flows in table 1 according to their contributions to the Born matrix element in eq. (6.38). Note that the flows $(\bar\gamma_{1,1}, \bar\gamma_{1,1})$ and $(\bar\gamma_{1,2}, \bar\gamma_{1,2})$ make negative contributions and would have to be treated as counter-events or with a resampling method such as that of refs. [38–40]. Showering of partons that contribute to the radiation pattern of the chosen flow, according to eqs. (6.22)–(6.27), then proceeds as in leading colour. However, if any of the subleading colour flows is chosen, then one or both of the gluons do not contribute and the power (7.9) is not defined for them. These are either gluons that appear twice in the same colour loop (in $(\bar\gamma_{0,1}, \bar\gamma_{0,2})$) or else (in $(\bar\gamma_{i,j}, \bar\gamma_{i,j})$ with $i = 1, 2$) the U(1) gluons discussed in section 5.2. They do not shower and can be treated in a manner similar to the self-connected gluons to be discussed in section 7.2.2.

For our other example process $\bar q q \to q' \bar q' g$, the situation is more complicated. The radiation patterns of the 13 contributing closed flows in table 2 are given in (6.74)–(6.86). Here some of the eikonal terms in subleading flows have negative coefficients, leading to negative contributions in eq. (7.9). Note however that the denominator is always $+1$ for quarks and antiquarks, and either 2 or 0 for gluons. In the latter case the gluon is non-showering as before. In the former, the gluon may have either two partners with coefficients $+1$ or four partners, three with $+1$ and one with $-1$. Similarly a quark or antiquark may have one partner or three. Consider for example the flow $(\bar\gamma_{0,1}, \bar\gamma_{0,4})$ (which is maximally colour-suppressed). According to eq. (6.77) the gluon is non-showering, while each quark or antiquark has two partners with coefficient $+1$ and one with coefficient $-1$. For example, quark $-1$ has antiquark partners $-3$ and $-4$ with coefficients $+1$ and quark partner $-2$ with coefficient $-1$ (this shows in a specific case the implications of the generalization beyond leading colour of colour connections/colour partners, which we discussed in section 6.4). There are six possible angular orderings

$$\xi_{-1,-2} > \xi_{-1,-3} > \xi_{-1,-4} \implies (-1, 0, 1)\,, \tag{7.10}$$
$$\xi_{-1,-2} > \xi_{-1,-4} > \xi_{-1,-3} \implies (-1, 0, 1)\,, \tag{7.11}$$
$$\xi_{-1,-3} > \xi_{-1,-2} > \xi_{-1,-4} \implies (1, 0, 1)\,, \tag{7.12}$$
$$\xi_{-1,-3} > \xi_{-1,-4} > \xi_{-1,-2} \implies (1, 2, 1)\,, \tag{7.13}$$
$$\xi_{-1,-4} > \xi_{-1,-2} > \xi_{-1,-3} \implies (1, 0, 1)\,, \tag{7.14}$$
$$\xi_{-1,-4} > \xi_{-1,-3} > \xi_{-1,-2} \implies (1, 2, 1)\,. \tag{7.15}$$

JHEP11(2021)045

The last entry in each case shows the corresponding sequence of values of the power (7.9), as $\xi_{-1}$ evolves from the highest to the lowest values. Note that the power is always 1 at the lowest angles, but can be 2 (higher emission), 0 (no emission) or even $-1$ at higher values. Negative powers, corresponding to a non-negative-definite Sudakov exponent, have already been considered in the literature (see e.g. refs. [41–43]), and can be dealt with for example by using a veto and weighting technique.

The incidence of negative flow contributions and/or negative powers (7.9) could lead to low Monte Carlo efficiency, which might be improved by not preselecting a flow, thus allowing all flows to contribute to shower evolution. This corresponds to using (7.4) directly to define the evolution power

$$p_{\rm SUM}(\xi_k) = \frac{\sum_l \mathcal{M}_{kl}^{(2q;n)}\,\Theta(\xi_{kl}-\xi_k)}{\sum_l \mathcal{M}_{kl}^{(2q;n)}} = \frac{\sum_l \mathcal{M}_{kl}^{(2q;n)}\,\Theta(\xi_{kl}-\xi_k)}{2C(\mathcal{I}_k)\mathcal{M}^{(2q;n)}} \tag{7.16}$$

in place of (7.9), where we have used the completeness relation (6.14). The colour-linked Borns $\mathcal{M}_{kl}^{(2q;n)}$ are given explicitly in (6.41)–(6.46) for $\bar{q}q \to gg$ and in (6.89)–(6.102) for $\bar{q}q \to q'\bar{q}'g$. Then since the leading-colour flows contribute to all stages of showering there will be on average a lower incidence of negative powers. Statistically, at the parton level the results should be equivalent to evolving flows separately. On the other hand, all colour information is lost by the end of the shower, which could be problematic for hadronization.

### 7.2 Dipole showers

A dipole Monte Carlo implementation including subleading colour will in general involve contributions from dipole configurations of all possible types, connecting quark-antiquark, quark-quark, quark-gluon, gluon-gluon and their conjugates. With each configuration we can associate a graph specifying the dipole connections. The theory of such graphs is linked to that of flows discussed above, as elaborated in the following subsection.

#### 7.2.1 Dipole graphs

For a given process that features $q$ quark-antiquark pairs (all considered as outgoing) and $n$ gluons let

$$q_s = \Big\{-2q, -2q+1, \ldots -1\Big\}, \tag{7.17}$$

$$g_s = \Big\{1, 2, \ldots n\Big\}, \tag{7.18}$$

be the sets of the quark/antiquark and gluon labels, respectively, in keeping with eqs. (2.2)–(2.4). We define a dipole graph to be a set of $q+n$ pairs, as follows:

$$\begin{aligned}\Gamma = \Big\{(\Gamma_{1,1},\Gamma_{1,2}),\ldots(\Gamma_{q+n,1},\Gamma_{q+n,2})\;\Big|\;&\Gamma_{\alpha,\beta}\in q_s\cup g_s\,,\ \forall\alpha\ \ \Gamma_{\alpha,1}\neq\Gamma_{\alpha,2}\,,\\ &\forall\,k\in q_s\ \ \exists!\ (\alpha,\beta)\ \ \text{s.t.}\ \ k=\Gamma_{\alpha,\beta}\ \ \text{and}\\ &\forall\,k\in g_s\ \ \exists!\ (\alpha_1,\beta_1)\,,(\alpha_2,\beta_2)\ \ \text{s.t.}\ \ k=\Gamma_{\alpha_1,\beta_1}=\Gamma_{\alpha_2,\beta_2}\Big\}\,.\end{aligned} \tag{7.19}$$

In other words: a dipole graph is a set of pairs of non-identical parton labels; each quark and antiquark (gluon) label will appear in it exactly once (twice). Each pair that belongs

JHEP11(2021)045

to a dipole graph is a dipole, and its two elements are the dipole ends. The order in which the pairs enter a dipole graph is irrelevant from a physics viewpoint: they can be re-shuffled as is convenient. We denote by

$$\mathcal{G}_{2q;n} = \Big\{\Gamma \,\Big|\, \Gamma(\text{eq. } (7.19))\Big\}\,, \tag{7.20}$$

the set of all dipole graphs relevant to $q$ quark lines and $n$ gluons. By analogy with eq. (6.12), we call the quantity

$$\mathcal{D}\,(\Gamma) = \sum_{\alpha=1}^{q+n} \Big[\Gamma_{\alpha,1}, \Gamma_{\alpha,2}\Big]\,, \tag{7.21}$$

the radiation pattern associated with the dipole graph $\Gamma$.

Any dipole graph admits a representation by means of a square $(2q+n)\times(2q+n)$ matrix,[14] defined as follows:

$$m(\Gamma) = \big(m(\Gamma)_{ij}\big)_{i,j=-2q}^{n}\,, \qquad m(\Gamma)_{ij} = \sum_{\alpha=1}^{q+n}\Big[\delta(i,\Gamma_{\alpha,1})\delta(j,\Gamma_{\alpha,2}) + \delta(j,\Gamma_{\alpha,1})\delta(i,\Gamma_{\alpha,2})\Big]\,. \tag{7.22}$$

That is, each dipole in $\Gamma$ gives a unit contribution to the matrix element whose indices coincides with the dipole ends, both in the given and in the reverse order. Owing to eq. (7.19), the matrix defined in eq. (7.22) has the following properties: *a)* it is symmetric; *b)* it is traceless; *c)* its elements are equal to either 0, 1, or 2; *d)* each of $2q$ of its rows sums to one, and each of the remaining $n$ rows sums to two. By construction

$$\mathcal{D}\,(\Gamma) = \frac{1}{2}\sum_{i,j=-2q}^{n} m(\Gamma)_{ij}\Big[i,j\Big]\,. \tag{7.23}$$

We stress that eqs. (7.21)–(7.23) are invariant under reordering of the dipoles that enter the given dipole graph.

The counting of distinct matrices of the type defined in eq. (7.22) tells us the number of possible dipole configurations for $q$ quark-antiquark pairs and $n$ gluons, $|\mathcal{G}_{2q;n}|$. It is convenient to define the generating function

$$\mathcal{F}(x,y) = \sum_{q,n} |\mathcal{G}_{2q;n}| \frac{x^q}{q!}\frac{y^n}{n!}\,, \tag{7.24}$$

so that

$$|\mathcal{G}_{2q;n}| = \left.\frac{\partial^{q+n}\mathcal{F}}{\partial x^q \partial y^n}\right|_{x,y=0}\,. \tag{7.25}$$

For a process involving only $q$ quark-antiquark pairs and no gluons, the dipole matrix is a $2q\times 2q$ symmetric, traceless matrix with elements 0 or 1 and each row summing to 1. It is readily seen that the number of distinct matrices of this type is $(2q-1)!!$, so that the generating function is

$$\mathcal{F}(x,0) = \sum_q \frac{(2q-1)!!}{q!}x^q = \frac{1}{\sqrt{1-2x}}. \tag{7.26}$$

[14]In keeping with the labeling of ref. [23] (see section 2), parton labels equal to zero must be ignored.

JHEP11(2021)045

| $q \diagdown n$ | 0 | 1 | 2 | 3 | 4 | 5 | 6 |
|---|---|---|---|---|---|---|---|
| 0 | 1 | 0 | 1 | 1 | 6 | 22 | 130 |
| 1 | 1 | 1 | 3 | 10 | 46 | 252 | 1642 |
| 2 | 3 | 6 | 21 | 93 | 510 | 3306 | 24762 |
| 3 | 15 | 45 | 195 | 1050 | 6750 | 50280 | 425490 |
| 4 | 105 | 420 | 2205 | 13965 | 103110 | 867510 | 8183490 |

**Table 3**. Number of distinct dipole graphs for $q$ quark-antiquark pairs and $n$ gluons.

In the case of purely gluonic processes, $q = 0$, the corresponding matrices have all rows and columns summing to 2. The counting of such matrices is a known problem, whose solution is given by the generating function [44]

$$\mathcal{F}(0, y) = \frac{\mathrm{e}^{-y/2+y^2/4}}{\sqrt{1-y}}. \tag{7.27}$$

For processes that involve both quarks and gluons, we can suppose that $n-k$ of the gluons are connected amongst themselves while $k$ are inserted onto one or more of the quark lines. All the permutations of the latter are distinct, and the number of ways of distributing them is the number of ways of locating $q-1$ subset boundaries amongst $k+q-1$ objects, so the number of distinct dipole graphs is[15]

$$|\mathcal{G}_{2q;n}| = \sum_{k=0}^{n} \binom{n}{k} \binom{k+q-1}{q-1} k!\, |\mathcal{G}_{0;n-k}|\, |\mathcal{G}_{2q;0}|\,, \tag{7.28}$$

which yields the generating function

$$\begin{aligned} \mathcal{F}(x, y) &= \mathcal{F}(0, y)\, \mathcal{F}(x/[1-y], 0) \\ &= \frac{\mathrm{e}^{-y/2+y^2/4}}{\sqrt{1-2x-y}}. \end{aligned} \tag{7.29}$$

This gives the numbers shown in table 3.

### 7.2.2 Self-connected gluons

At leading order in colour, and also at subleading order for processes involving only quarks or only gluons, the eikonal combinations that appear in the differential cross section for emission of an additional soft gluon can be represented by a weighted sum of dipole graphs involving all the partons of the primary process. In processes involving both quarks and gluons, however, at subleading order in colour, it is in general necessary to include graphs with fewer participating gluons than in the primary process. In essence, this is due to the presence of gluons both of whose colour labels enter the same colour loop; as is shown in section 6.4, such gluons (denoted by $g^\star$ there) cannot contribute to radiation patterns. Further discussions on this point will be given in section 7.4 and appendix B.

[15]Note therefore that this is analogous to the counting problem that leads one to eq. (2.11).

JHEP11(2021)045

| $q\diagdown n$ | 0 | 1 | 2 | 3 | 4 | 5 | 6 |
|---|---|---|---|---|---|---|---|
| 0 | 1 | 1 | 2 | 5 | 17 | 73 | 388 |
| 1 | 1 | 2 | 6 | 23 | 109 | 618 | 4096 |
| 2 | 3 | 9 | 36 | 177 | 1035 | 7029 | 54462 |
| 3 | 15 | 60 | 300 | 1785 | 12315 | 96720 | 852630 |
| 4 | 105 | 525 | 3150 | 21945 | 173985 | 1546965 | 15250200 |

**Table 4**. Number of distinct dipole graphs for $q$ quark-antiquark pairs and $n$ gluons, allowing any number of self-connected gluons (see eq. (7.32)).

Although such non-participating gluons are not associated with eikonal terms in the soft gluon cross section, they cannot be altogether ignored in a Monte Carlo implementation, since to do so would violate momentum conservation. Instead they can be treated as having self-connected colour and anticolour. Such a self-connected gluon $i$ is associated with a null eikonal $[i,i]$, which cannot radiate: it propagates without showering and must be hadronized through momentum transfer with other showers in the same event.

To make connection with the theory of dipole graphs, for any $0 \le p \le n$ and $s_p \in S_p^{(n)}$ as in eq. (5.11), we define

$$\mathcal{G}_{2q;n-p,s_p} = \left\{\Gamma \;\middle|\; \Gamma(\text{eq. } (7.19), g_s \longrightarrow g_s \setminus s_p)\right\} \tag{7.30}$$

to be the set of $q$-quark, $(n-p)$-gluon dipole graphs, where the gluon labels do not assume the values in the set $s_p$. The gluons whose labels belong to the set $s_p$ are understood to be self-connected gluons. Note that

$$p = 0 \quad \Longrightarrow \quad s_0 = \{\} \quad \Longrightarrow \quad \mathcal{G}_{2q;n,s_0} \equiv \mathcal{G}_{2q;n} \,. \tag{7.31}$$

For a process involving $q$ quark-antiquark pairs and $n$ gluons, the number of relevant dipole graphs where we allow any number of self-connected gluons is then (see eq. (5.13))

$$\sum_{p=0}^{n} \left|\mathcal{G}_{2q;n-p,s_p}\right| = \sum_{p=0}^{n} \binom{n}{p} \left|\mathcal{G}_{2q;n-p}\right| \,. \tag{7.32}$$

If we denote the generating function for this by $\widetilde{\mathcal{F}}(x,y)$, then

$$\widetilde{\mathcal{F}}(x,y) = \mathrm{e}^{y}\mathcal{F}(x,y) = \frac{\mathrm{e}^{y/2+y^2/4}}{\sqrt{1-2x-y}}, \tag{7.33}$$

which gives the numbers shown in table 4.

## 7.3 Examples

### 7.3.1 $\bar{q}q \to gg$

Consider again the process $\bar{q}q \to gg$. According to tables 3 and 4 there are six relevant dipole graphs, $\Gamma_1 \ldots \Gamma_6$, of which three involve self-connected gluons, corresponding to the six dipole configurations shown in figure 2. The corresponding radiation patterns are

JHEP11(2021)045

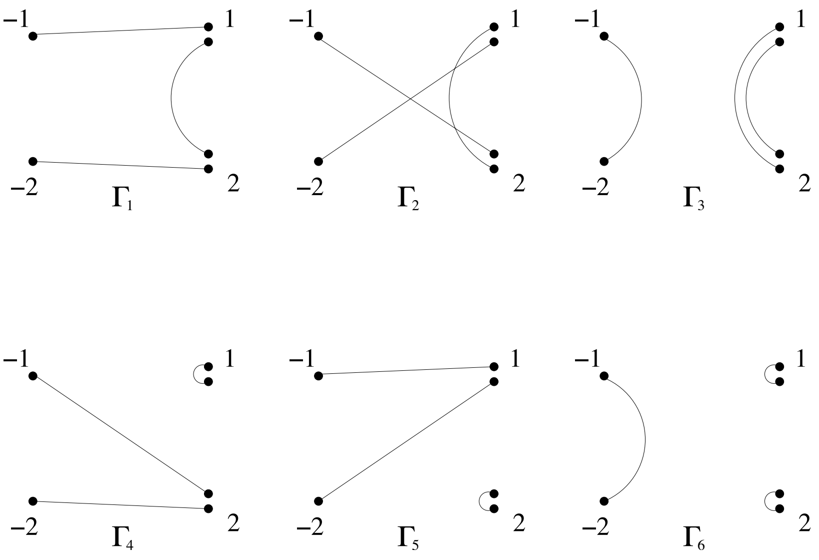

**Figure 2**. Dipole graphs for $q\bar{q} \to gg$. In the cases of $\Gamma_4$, $\Gamma_5$, and $\Gamma_6$ the corresponding self-connected gluons are also depicted.

$$\mathcal{D}\left(\Gamma_1\right) = \Big[-1,1\Big] + \Big[-2,2\Big] + \Big[1,2\Big]\,, \tag{7.34}$$

$$\mathcal{D}\left(\Gamma_2\right) = \Big[-1,2\Big] + \Big[-2,1\Big] + \Big[1,2\Big]\,, \tag{7.35}$$

$$\mathcal{D}\left(\Gamma_3\right) = \Big[-1,-2\Big] + 2\Big[1,2\Big]\,, \tag{7.36}$$

$$\mathcal{D}\left(\Gamma_4\right) = \Big[-1,2\Big] + \Big[-2,2\Big]\,, \tag{7.37}$$

$$\mathcal{D}\left(\Gamma_5\right) = \Big[-1,1\Big] + \Big[-2,1\Big]\,, \tag{7.38}$$

$$\mathcal{D}\left(\Gamma_6\right) = \Big[-1,-2\Big]\,. \tag{7.39}$$

Note that these are not independent, since

$$\mathcal{D}\left(\Gamma_1\right) + \mathcal{D}\left(\Gamma_2\right) + \mathcal{D}\left(\Gamma_6\right) = \mathcal{D}\left(\Gamma_3\right) + \mathcal{D}\left(\Gamma_4\right) + \mathcal{D}\left(\Gamma_5\right). \tag{7.40}$$

The closed-flow radiation patterns (6.22)–(6.27) can be expressed in terms of the above dipole radiation patterns as follows:

$$\mathcal{E}(\bar{\gamma}_{0,1},\bar{\gamma}_{0,1}) = \mathcal{D}\left(\Gamma_1\right)\,, \tag{7.41}$$

$$\mathcal{E}(\bar{\gamma}_{0,1},\bar{\gamma}_{0,2}) = \mathcal{D}\left(\Gamma_6\right)\,, \tag{7.42}$$

$$\mathcal{E}(\bar{\gamma}_{0,2},\bar{\gamma}_{0,2}) = \mathcal{D}\left(\Gamma_2\right)\,, \tag{7.43}$$

JHEP11(2021)045

$$\mathcal{E}(\bar{\gamma}_{1,1}, \bar{\gamma}_{1,1}) = \mathcal{D}\,(\Gamma_4)\ , \tag{7.44}$$
$$\mathcal{E}(\bar{\gamma}_{1,2}, \bar{\gamma}_{1,2}) = \mathcal{D}\,(\Gamma_5)\ , \tag{7.45}$$
$$\mathcal{E}(\bar{\gamma}_{2,1}, \bar{\gamma}_{2,1}) = \mathcal{D}\,(\Gamma_6)\ . \tag{7.46}$$

Thus, for the soft radiation matrix element (6.39) we now have

$$\begin{aligned}\frac{2\mathcal{M}^{(2;3)}_{\text{SOFT}}}{g_S^2 C_F} &= N^3\mathcal{D}\,(\Gamma_1)\,M_1 + N^3\mathcal{D}\,(\Gamma_2)\,M_3 + N\mathcal{D}\,(\Gamma_6)\,M_2 \\ &\quad - \frac{1}{N}\,N^2\left[\mathcal{D}\,(\Gamma_4) + \mathcal{D}\,(\Gamma_5)\right]M_4 + \frac{1}{N^2}\,N\mathcal{D}\,(\Gamma_6)\,M_4\,.\end{aligned} \tag{7.47}$$

Using (7.40) we may, for example, eliminate $\mathcal{D}\,(\Gamma_6)$ which involves two self-connected gluons.

#### 7.3.2 $\bar{q}q \to q'\bar{q}'g$

There are 9 relevant dipole graphs, shown in figure 3, with associated radiation patterns

$$\mathcal{D}\,(\Gamma_1) = \Big[-1, 1\Big] + \Big[1, -3\Big] + \Big[-2, -4\Big]\,, \tag{7.48}$$
$$\mathcal{D}\,(\Gamma_2) = \Big[-1, -3\Big] + \Big[-2, 1\Big] + \Big[1, -4\Big]\,, \tag{7.49}$$
$$\mathcal{D}\,(\Gamma_3) = \Big[-1, 1\Big] + \Big[1, -4\Big] + \Big[-2, -3\Big]\,, \tag{7.50}$$
$$\mathcal{D}\,(\Gamma_4) = \Big[-1, -4\Big] + \Big[-2, 1\Big] + \Big[1, -3\Big]\,, \tag{7.51}$$
$$\mathcal{D}\,(\Gamma_5) = \Big[-1, 1\Big] + \Big[1, -2\Big] + \Big[-3, -4\Big]\,, \tag{7.52}$$
$$\mathcal{D}\,(\Gamma_6) = \Big[-1, -2\Big] + \Big[-3, 1\Big] + \Big[1, -4\Big]\,, \tag{7.53}$$
$$\mathcal{D}\,(\Gamma_7) = \Big[-1, -3\Big] + \Big[-2, -4\Big]\,, \tag{7.54}$$
$$\mathcal{D}\,(\Gamma_8) = \Big[-1, -4\Big] + \Big[-2, -3\Big]\,, \tag{7.55}$$
$$\mathcal{D}\,(\Gamma_9) = \Big[-1, -2\Big] + \Big[-3, -4\Big]\,. \tag{7.56}$$

The closed-flow radiation patterns (6.74)–(6.86) can be expressed in terms of these as follows:

$$\mathcal{E}(\bar{\gamma}_{0,1}, \bar{\gamma}_{0,1}) = \mathcal{D}\,(\Gamma_1)\ , \tag{7.57}$$

JHEP11(2021)045

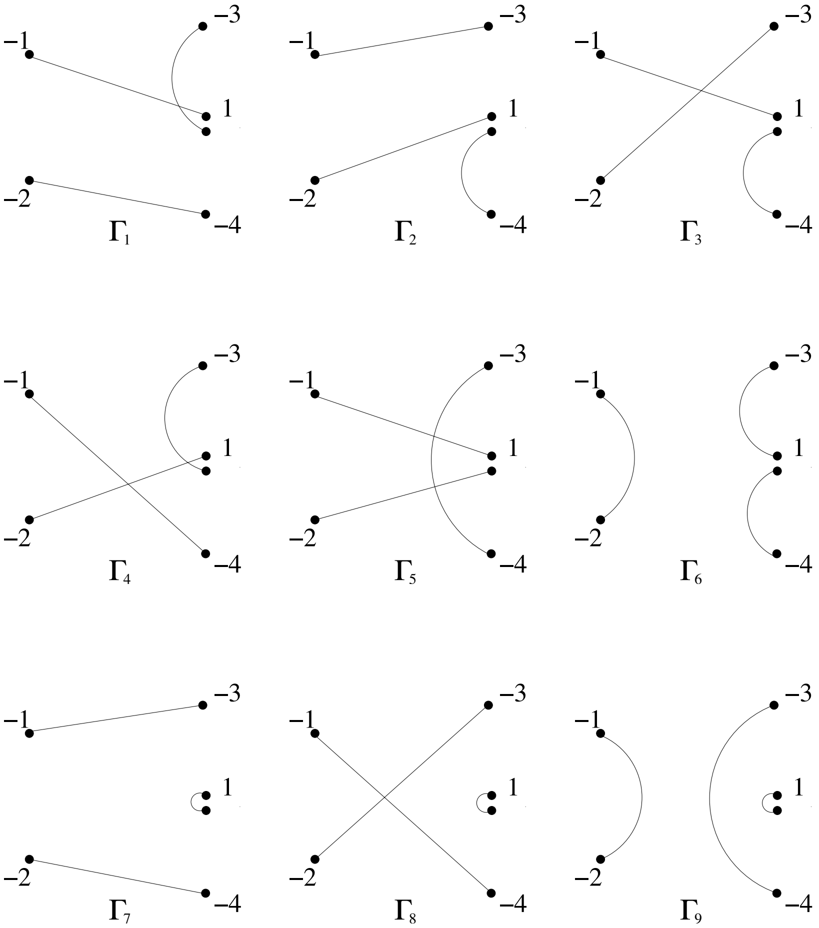

**Figure 3**. Dipole graphs for $q\bar{q} \to q'\bar{q}'g$. In the cases of $\Gamma_7$, $\Gamma_8$, and $\Gamma_9$ the self-connected gluon is also depicted.

$$\mathcal{E}(\bar{\gamma}_{0,1}, \bar{\gamma}_{0,2}) = \mathcal{D}\left(\Gamma_1\right) + \mathcal{D}\left(\Gamma_3\right) - \mathcal{D}\left(\Gamma_5\right) \,, \tag{7.58}$$

$$\mathcal{E}(\bar{\gamma}_{0,1}, \bar{\gamma}_{0,3}) = \mathcal{D}\left(\Gamma_1\right) + \mathcal{D}\left(\Gamma_4\right) - \mathcal{D}\left(\Gamma_6\right) \,, \tag{7.59}$$

$$\mathcal{E}(\bar{\gamma}_{0,1}, \bar{\gamma}_{0,4}) = \mathcal{D}\left(\Gamma_7\right) + \mathcal{D}\left(\Gamma_8\right) - \mathcal{D}\left(\Gamma_9\right) \,, \tag{7.60}$$

$$\mathcal{E}(\bar{\gamma}_{0,2}, \bar{\gamma}_{0,2}) = \mathcal{D}\left(\Gamma_3\right) \,, \tag{7.61}$$

$$\mathcal{E}(\bar{\gamma}_{0,2}, \bar{\gamma}_{0,3}) = \mathcal{D}\left(\Gamma_7\right) + \mathcal{D}\left(\Gamma_8\right) - \mathcal{D}\left(\Gamma_9\right) \,, \tag{7.62}$$

$$\mathcal{E}(\bar{\gamma}_{0,2}, \bar{\gamma}_{0,4}) = \mathcal{D}\left(\Gamma_2\right) + \mathcal{D}\left(\Gamma_3\right) - \mathcal{D}\left(\Gamma_6\right) \,, \tag{7.63}$$

$$\mathcal{E}(\bar{\gamma}_{0,3}, \bar{\gamma}_{0,3}) = \mathcal{D}\left(\Gamma_4\right) \,, \tag{7.64}$$

$$\mathcal{E}(\bar{\gamma}_{0,3}, \bar{\gamma}_{0,4}) = \mathcal{D}\left(\Gamma_2\right) + \mathcal{D}\left(\Gamma_4\right) - \mathcal{D}\left(\Gamma_5\right) \,, \tag{7.65}$$

$$\mathcal{E}(\bar{\gamma}_{0,4}, \bar{\gamma}_{0,4}) = \mathcal{D}\left(\Gamma_2\right) \,, \tag{7.66}$$

$$\mathcal{E}(\bar{\gamma}_{1,1}, \bar{\gamma}_{1,1}) = \mathcal{D}\left(\Gamma_7\right) \,, \tag{7.67}$$

$$\mathcal{E}(\bar{\gamma}_{1,1}, \bar{\gamma}_{1,2}) = \mathcal{D}\left(\Gamma_7\right) + \mathcal{D}\left(\Gamma_8\right) - \mathcal{D}\left(\Gamma_9\right) \,, \tag{7.68}$$

$$\mathcal{E}(\bar{\gamma}_{1,2}, \bar{\gamma}_{1,2}) = \mathcal{D}\left(\Gamma_8\right) \,. \tag{7.69}$$

JHEP11(2021)045

We note the following identities:

$$\mathcal{D}(\Gamma_1) + \mathcal{D}(\Gamma_2) + \mathcal{D}(\Gamma_8) = \mathcal{D}(\Gamma_3) + \mathcal{D}(\Gamma_4) + \mathcal{D}(\Gamma_7) \;, \tag{7.70}$$

$$\mathcal{D}(\Gamma_3) + \mathcal{D}(\Gamma_4) + \mathcal{D}(\Gamma_9) = \mathcal{D}(\Gamma_5) + \mathcal{D}(\Gamma_6) + \mathcal{D}(\Gamma_8) \;, \tag{7.71}$$

which allow one to eliminate two (but not all) of the dipole graphs with a self-connected gluon.

### 7.4 Closed colour flows versus dipole graphs

In this section, we shall show how the radiation patterns that stem from closed colour flows, eq. (6.12), can be represented in terms of analogous quantities, defined as properties of suitably-chosen dipole graphs, which involve their radiation patterns of eq. (7.21).

In order to do so, we start by observing that the radiation pattern of any closed flow $(\bar\gamma', \bar\gamma)$ can be decomposed into the radiation patterns of the individual colour loops associated with that flow, thus

$$\mathcal{E}(\bar\gamma', \bar\gamma) = \sum_{\ell \in \mathcal{L}(\bar\gamma', \bar\gamma)} \mathcal{E}_\ell(\bar\gamma', \bar\gamma) \,, \qquad \mathcal{E}_\ell(\bar\gamma', \bar\gamma) = \sum_{k,l\in\ell}^{k<l} (-1)^{\delta_\ell(k,l)} \Big[k, l\Big] \,. \tag{7.72}$$

Furthermore, the reader must bear in mind that the number of elements in a colour loop is an even number; half of the elements are L-elements, the other half are R-elements (see section 3); they are alternating, beginning with an R-element. We write this as follows:

$$\ell \in \mathcal{L}(\bar\gamma', \bar\gamma) \,, \qquad \ell = (e_1, e_2, \ldots e_{2m_\ell}) \,, \tag{7.73}$$

so that $2m_\ell$ is the number of elements in the loop, and

$$\mathcal{E}_\ell\left(\bar\gamma', \bar\gamma\right) = \sum_{i=1}^{2m_\ell-1} \sum_{j=1}^{\lceil (2m_\ell - i)/2 \rceil} \Big[e_i, e_{i+2j-1}\Big] - \sum_{i=1}^{2m_\ell-1} \sum_{j=1}^{\lfloor (2m_\ell - i)/2 \rfloor} \Big[e_i, e_{i+2j}\Big] \tag{7.74}$$

$$= \mathcal{E}_\ell^{+}\left(\bar\gamma', \bar\gamma\right) - \mathcal{E}_\ell^{-}\left(\bar\gamma', \bar\gamma\right) \,. \tag{7.75}$$

There are

$$\sum_{k=1}^{2m_\ell - 1} \left\lceil \frac{k}{2} \right\rceil = m_\ell^2 \tag{7.76}$$

and

$$\sum_{k=1}^{2m_\ell - 1} \left\lfloor \frac{k}{2} \right\rfloor = m_\ell\,(m_\ell - 1) \tag{7.77}$$

summands in $\mathcal{E}_\ell^{+}(\bar\gamma', \bar\gamma)$ and $\mathcal{E}_\ell^{-}(\bar\gamma', \bar\gamma)$, respectively. Note that

$$m_\ell^2 + m_\ell(m_\ell - 1) = \frac{2m_\ell(2m_\ell - 1)}{2} \,, \tag{7.78}$$

is indeed the total number of summands in $\mathcal{E}_\ell(\bar\gamma', \bar\gamma)$ (before any algebraic simplifications).

Since the two sums in eq. (7.75) are linear combinations of eikonals with coefficients all equal to one, we see the possible emergence of radiation patterns of dipole graphs

JHEP11(2021)045

in eq. (7.72). More precisely, the argument goes as follows. Equation (7.76) suggests organizing the parton labels associated with the summands in $\mathcal{E}_\ell^+(\bar\gamma',\bar\gamma)$ in $m_\ell$ sets, each composed of $m_\ell$ pairs of labels. Likewise, from eq. (7.77) one obtains $m_\ell-1$ sets, each composed of $m_\ell$ pairs of labels, relevant to $\mathcal{E}_\ell^-(\bar\gamma',\bar\gamma)$. These sets of pairs of labels are candidates to be subsets of suitable dipole graphs. For this to happen, all quark and antiquark labels that belong to the colour loop $\ell$ must appear exactly once in each of such sets, whereas gluon labels must appear at least once and at most twice. These properties must be fulfilled for each of the loops in $\mathcal{L}(\bar\gamma',\bar\gamma)$, so that when the candidate subsets are combined one forms properly-defined dipole graphs. This can happen since

$$\sum_\ell m_\ell = q+n\,, \tag{7.79}$$

that is, the number of elements in the joined candidate subsets is equal to the number of dipoles in the dipole graphs. The fact that candidate subsets emerging from different loops have disjoint sets of labels is trivially true for quarks and antiquarks, but not necessarily so for gluons (see items i.–iv. in section 3). We point out that there are

$$\prod_\ell m_\ell\,,\qquad \prod_\ell\left(m_\ell-1\right)\,, \tag{7.80}$$

possible ways of joining the candidate subsets that emerge from different loops, for $\mathcal{E}_\ell^+(\bar\gamma',\bar\gamma)$ and $\mathcal{E}_\ell^-(\bar\gamma',\bar\gamma)$, respectively.

In appendix B we shall sketch a proof of the equivalence of colour flows and dipole graphs. Here, we limit ourselves to stating that the consequence of this equivalence is the existence, for any given closed colour flow $(\bar\gamma',\bar\gamma)$, of two sets of dipole graphs

$$\Big\{\Gamma_a\Big\}^+_{(\bar\gamma',\bar\gamma)}\,,\qquad \Big\{\Gamma_b\Big\}^-_{(\bar\gamma',\bar\gamma)}\,, \tag{7.81}$$

such that

$$\sum_{\ell\in\mathcal{L}(\bar\gamma',\bar\gamma)}\mathcal{E}_\ell^+(\bar\gamma',\bar\gamma)=\sum_a c^+\left(\Gamma_a\right)\,\mathcal{D}\left(\Gamma_a\right)\,, \tag{7.82}$$

$$\sum_{\ell\in\mathcal{L}(\bar\gamma',\bar\gamma)}\mathcal{E}_\ell^-(\bar\gamma',\bar\gamma)=\sum_b c^-\left(\Gamma_b\right)\,\mathcal{D}\left(\Gamma_b\right)\,, \tag{7.83}$$

for suitable *integer* coefficients $c^\pm(\Gamma)$ (which, as we shall show in appendix B, are typically equal to one). We point out that the sets in eq. (7.81) are not necessarily disjoint, and that their choice is not necessarily unique.

While the proof of eqs. (7.82) and (7.83) is complicated in general, an exception is that of a diagonal closed flow (i.e. one for which the L- and R-flow coincide, $(\bar\gamma,\bar\gamma)$). In that case, there are $q+n$ colour loops,[16] each with two elements; that is, $m_\ell=1\ \forall\,\ell$, and

$$\mathcal{E}_\ell^-(\bar\gamma,\bar\gamma)=0\quad\Longrightarrow\quad\mathcal{E}_\ell(\bar\gamma,\bar\gamma)=\mathcal{E}_\ell^+(\bar\gamma,\bar\gamma)\,. \tag{7.84}$$

[16] In order to be definite, here we consider the case of zero U(1) gluons. The results can then be applied to the case of $p$ U(1) gluons by means of the formal replacement $n\to n-p$.

JHEP11(2021)045

Using eqs. (2.5) and (2.6)

$$\mathcal{L}(\bar{\gamma},\bar{\gamma}) = \bigcup_{p=1}^{q}\Big\{\Big(-p,\sigma(t_{p-1}+1)\Big),\Big(\sigma(t_{p-1}+1),\sigma(t_{p-1}+2)\Big),\ldots$$
$$\Big(\sigma(t_p-1),\sigma(t_p)\Big),\Big(\sigma(t_p),\mu(-p-q)\Big)\Big\}. \qquad (7.85)$$

The set on the r.h.s. of eq. (7.85) is a dipole graph that belongs to $\mathcal{G}_{2q;n}$; therefore, the leftmost set in eq. (7.81) contains a single element, and the rightmost one is empty.

When the L- and R-flows do not coincide the sums on the r.h.s. of eqs. (7.82) and (7.83) are non-trivial. Without knowing their explicit forms, one can still establish a rather general property, namely that the leftmost set in eq. (7.81) is not a subset of $\mathcal{G}_{2q;n}$, but rather of $\cup_{p,s_p}\mathcal{G}_{2q;n-p,s_p}$, for suitable values of $p$ and $s_p$. The argument is the following: a necessary condition for the following relations to hold true

$$\Big\{\Gamma_a\Big\}^{+}_{(\bar{\gamma}',\bar{\gamma})} \subseteq \mathcal{G}_{2q;n} \qquad (7.86)$$

is the absence of self-connected gluons. Indeed, when this is not the case, of the $m_\ell^2$ summands in the leftmost sum in eq. (7.74) there are $n_{g^\star,\ell}$ which are equal to zero, being self-eikonals. Here, by $n_{g^\star,\ell}$ we have denoted the number of gluon labels that appear twice in the loop $\ell$. Therefore, in general out of $m_\ell^2 - n_{g^\star,\ell}$ pairs one cannot identify an integer number of non-overlapping sets (the candidate subsets of suitable dipole graphs), each with $m_\ell$ pairs of labels. The fact that in general $n_{g^\star,\ell} \neq 0$ for some $\ell$ implies that eq. (7.86) does not hold true, and must be modified as follows:

$$\Big\{\Gamma_a\Big\}^{+}_{(\bar{\gamma}',\bar{\gamma})} \subseteq \bigcup_{p,s_p}\mathcal{G}_{2q;n-p,s_p}\,. \qquad (7.87)$$

We point out that this is necessary in spite of the absence of U(1) gluons in the flow (or, more precisely, for any given number of U(1) gluons — see footnote 16), which further clarifies the fact that the $p$ gluons missing from the sets on the r.h.s. of eq. (7.87) are not U(1) gluons, but rather the self-connected ones introduced in section 7.2.2. In order to avoid any confusion, when possible we shall number the self-connected and U(1) gluons by means of labels $k$ and $p$, respectively.

Thus, for each $0 \le k \le n$ and $s_k \in S_k^{(n)}$, we construct the subsets

$$\overline{\mathcal{G}}_{q;n-k,s_k} \subseteq \mathcal{G}_{2q;n-k,s_k} \qquad (7.88)$$

so that

$$\bigcup_{p=0}^{n}\ \bigcup_{s_p\in S_p^{(n)}}\ \bigcup_{\bar{\gamma},\bar{\gamma}'\in\mathcal{F}_{2q;n-p}^{(s_p)}}\left(\Big\{\Gamma_a\Big\}^{+}_{(\bar{\gamma}',\bar{\gamma})}\bigcup\Big\{\Gamma_b\Big\}^{-}_{(\bar{\gamma}',\bar{\gamma})}\right) = \bigcup_{k=0}^{n}\ \bigcup_{s_k\in S_k^{(n)}}\overline{\mathcal{G}}_{2q;n-k,s_k}\,. \qquad (7.89)$$

By inserting eqs. (7.82) and (7.83) into eq. (6.11) we arrive at an alternative expression for the soft limit of a generic quark-gluon matrix element

$$\mathcal{M}^{(2q;n+1)}_{\rm SOFT} = 2g_S^2\,C_F\sum_{k=0}^{n}\ \sum_{s_k\in S_k^{(n)}}\ \sum_{\Gamma\in\overline{\mathcal{G}}_{2q;n-k,s_k}}\widetilde{\mathcal{M}}^{(2q;n)}\left(\Gamma\right)\mathcal{D}\left(\Gamma\right)\,, \qquad (7.90)$$

JHEP11(2021)045

where

$$\widetilde{\mathcal{M}}^{(2q;n)}(\Gamma) = \sum_{p=0}^{n} \sum_{s_p \in S_p^{(n)}} \frac{(-1)^p}{2^n} N^{-p} \sum_{\bar{\gamma},\bar{\gamma}' \in \mathcal{F}_{2q;n-p}^{(s_p)}} \bar{\mathcal{A}}^{(2q;n)}(\bar{\gamma}')^\star \bar{\mathcal{A}}^{(2q;n)}(\bar{\gamma})$$
$$\times N^{-\rho(\bar{\gamma})-\rho(\bar{\gamma}')} N^{|\mathcal{L}(\bar{\gamma}',\bar{\gamma})|}$$
$$\times \left[ c^+(\Gamma)\, \delta\Big(\Gamma \in \big\{\Gamma_a\big\}^+_{(\bar{\gamma}',\bar{\gamma})}\Big) - c^-(\Gamma)\, \delta\Big(\Gamma \in \big\{\Gamma_b\big\}^-_{(\bar{\gamma}',\bar{\gamma})}\Big)\right]. \tag{7.91}$$

We point out that the matrix elements defined in eq. (7.91), at variance with all of their counterparts used so far, generally contain contributions associated with different powers of $N$. Because of this, it is necessary to include the monomials in $N$ into their definitions, again contrary to what was done so far. There is an ultimate physical reason for that. Namely, the structure of dipole graphs, although it extends that of the colour connections employed in current leading-colour PSMCs, is still based on a picture which takes a single side of the Cutkosky cut into account. However, the results of section 6 show that beyond leading colour it is necessary to include simultaneously the information on both sides of the cut, which can be done by means of closed colour loops. If the more complicated structure of such loops is traded for the simpler one of the dipole graphs, this entails a set of more complicated matrix elements.

By construction, the sets on the l.h.s. of eq. (7.88) may not be unique. However, different choices of $\overline{\mathcal{G}}_{q,n-k,s_k}$, while changing the individual summands on the r.h.s. of eq. (7.90), will leave the sum invariant — in other words, there are subsets of dipole graphs whose radiation patterns are not linearly independent of one another, as we have seen in the examples of section 7.3. With increasing numbers of quarks and gluons, the possibilities of choosing different $\overline{\mathcal{G}}_{q,n-k,s_k}$ also increase. This flexibility may turn out to be useful, since a suitable choice could lead to a reduction of negative weights.

### 7.5 Subevents

In order to implement a dipole shower simulation including subleading colour, one needs to take account of all relevant dipole graphs for a given hard process configuration, and then combine the results. This is necessary because, as we have seen, contributions from graphs with self-connected gluons cannot in general be avoided. Simply choosing a single one out of the set of dipole graphs according to their relative weights would lead to final states where hard hadrons appear that stem from non-showering self-connected gluons, and are mostly isolated. Instead, those hadrons can be more conveniently represented as components of a jet that includes showering of other contributing graphs. We call each dipole graph contribution a *subevent* of that hard configuration. The final state of that event is then the result of summing all subevents.

As an example, consider the process $\bar{q}q \to gg$ studied in section 7.3.1. For any given Born configuration, one would generate six subevents by showering and hadronization starting from the dipole configurations in graphs $\Gamma_1 \dots \Gamma_6$ of figure 2, respectively. These subevents would then be weighted by the corresponding coefficients $N^3 M_1 \dots M_4/N$ in

JHEP11(2021)045

eq. (7.47), then combined to produce a complete Monte Carlo event from that Born configuration. Each final-state object would be entered into histograms with the weight of the subevent that generated it. In this example, all the products of dual amplitudes $M_1 \dots M_4$, given in eqs. (6.34)–(6.37), are positive, so (7.47) implies that only the subevents from graphs $\Gamma_4$ and $\Gamma_5$ have negative weights, while $\Gamma_3$ does not contribute. Alternatively, we may use eq. (7.40) to eliminate $\Gamma_4$ and $\Gamma_5$ (or $\Gamma_6$), but then other subevents will get negative weights for certain Born configurations. This flexibility in the dipole graph approach may be useful for optimizing Monte Carlo efficiency. A possible way to deal with the remaining negative weights could be a resampling method such as that of refs. [38–40].

A similar approach would appear necessary in single-parton shower implementations that treat individual colour flows separately, as in eq. (7.9). Flows that do not involve one or more gluons from the Born process will again give rise to isolated hard hadrons coming from such non-showering gluons. If the result of showering each flow is treated as a subevent from the same Born configuration, these hadrons become a subleading correction to the hard component of the associated gluon jet.

## 8 The mixed QCD-QED case

An interesting aspect of the formulation presented in this paper is that it applies, essentially unchanged, to a mixed-coupling scenario, i.e. one in which perturbative expansions are obtained as power series in two parameters, both of which are regarded as "small". The foremost candidate is that of QCD+QED, where the two parameters alluded to before are the coupling constants, $\alpha_S$ and $\alpha$, of the respective theories; in order to be definite, in the following we shall understand this case. We point out that while $\alpha \ll \alpha_S$, the existence of a clear hierarchy between the expansion parameters is not a necessary condition for this formulation to work. We also remind the reader that matrix elements are proportional to $\alpha_S^n \alpha^m$; at the LO, $n$ and $m$ are constrained to obey $n+m = k_0$, with $k_0$ a process-dependent integer, while at the NLO $n + m = k_0 + 1$, and so forth. The interested reader can find in ref. [32] a classification scheme for the various contributions to a given N$^k$LO accuracy.

In the QCD+QED case, several kinds of results become relevant at the LO and NLO accuracy for any given combination $\alpha_S^n \alpha^m$:

*a)* tree-level matrix elements;

*b)* soft-gluon emission patterns;

*c)* soft-photon emission patterns.

The statement we make is that the formulae of section 5 can be used, with minimal or no changes, to deal with item *a)*, and those of section 6 to deal with item *b)*; as far as item *c)* is concerned, its treatment entails a significantly simplified version of its gluon-emission counterpart.

A detailed discussion of the items above is beyond the scope of this paper. Therefore, here we shall limit ourselves to presenting a simple example, which should be sufficient to give the reader an idea of the procedures to follow in a general case.

JHEP11(2021)045

• *Example.* Consider the following process:

$$0 \longrightarrow u(-1)\,\bar{u}(-3)\,u(-2)\,\bar{u}(-4)\,. \tag{8.1}$$

Incidentally, the fact that there are two quark-antiquark pairs of the same flavour allows us to show how to handle in practice such a case, which has been neglected so far in the interest of a simpler notation. Following ref. [23], we start by distinguishing flavour-line configurations

$$f_A = \{-1,-3\}\{-2,-4\}\,, \tag{8.2}$$
$$f_B = \{-1,-4\}\{-2,-3\}\,, \tag{8.3}$$

i.e. $f_A$ ($f_B$) corresponds to the graphs where there is a fermion line that connects quark $-1$ with antiquark $-3$ ($-4$), and quark $-2$ with antiquark $-4$ ($-3$). Conventionally, we identify $f_A$- and $f_B$-type graphs with $s$- and $t$-channel exchanges, respectively.

Although the two flavour-line configurations of eqs. (8.2) and (8.3) induce the same colour flows, such flows need to be distinguished, because their contributions to the matrix elements must be accounted for separately (also in view of the fact that the associated colour factors might differ). In the case of gluon exchange we have

$$\gamma_{1,A} = (-1,-4)(-2-3) \equiv \gamma_{2,B}\,, \tag{8.4}$$
$$\gamma_{2,A} = (-1,-3)(-2-4) \equiv \gamma_{1,B}\,, \tag{8.5}$$

whereas in the case of a photon exchange

$$\gamma_{3,A} \equiv \gamma_{2,A}\,, \tag{8.6}$$
$$\gamma_{3,B} \equiv \gamma_{1,A}\,. \tag{8.7}$$

According to eq. (3.32) of ref. [23]

$$\rho(\gamma_{1,A}) = 0\,, \qquad \rho(\gamma_{2,A}) = 1\,, \tag{8.8}$$
$$\rho(\gamma_{1,B}) = 0\,, \qquad \rho(\gamma_{2,B}) = 1\,, \tag{8.9}$$
$$\rho(\gamma_{3,A}) = 0\,, \qquad \rho(\gamma_{3,B}) = 0\,. \tag{8.10}$$

In view of eqs. (8.4)–(8.7), the only independent colour-loop structures are the following:

$$\mathcal{L}\left(\gamma_{1,A},\gamma_{1,A}\right) = \Big\{(-1,-4),(-2,-3)\Big\}\,, \tag{8.11}$$
$$\mathcal{L}\left(\gamma_{2,A},\gamma_{1,A}\right) = \Big\{(-1,-4,-2,-3)\Big\}\,, \tag{8.12}$$
$$\mathcal{L}\left(\gamma_{2,A},\gamma_{2,A}\right) = \Big\{(-1,-3),(-2,-4)\Big\}\,, \tag{8.13}$$

whence one derives the independent radiation patterns

$$\mathcal{E}\left(\gamma_{1,A},\gamma_{1,A}\right) = \Big[-1,-4\Big] + \Big[-2,-3\Big]\,, \tag{8.14}$$
$$\mathcal{E}\left(\gamma_{2,A},\gamma_{1,A}\right) = \Big[-1,-4\Big] - \Big[-1,-2\Big] + \Big[-1,-3\Big] \tag{8.15}$$
$$+ \Big[-4,-2\Big] - \Big[-4,-3\Big] + \Big[-2,-3\Big]\,, \tag{8.16}$$
$$\mathcal{E}\left(\gamma_{2,A},\gamma_{2,A}\right) = \Big[-1,-3\Big] + \Big[-2,-4\Big]\,. \tag{8.17}$$

JHEP11(2021)045

All of the other combinations are identical to those above, according to the identifications of eqs. (8.4)–(8.7). Conversely, as far as the dual amplitudes associated with the colour flows are concerned, we have the following:

$$\widehat{\mathcal{A}}^{(4;0)}(\gamma_{1,A}) = \mathcal{A}_s\,, \qquad \widehat{\mathcal{A}}^{(4;0)}(\gamma_{2,A}) = -\mathcal{A}_s\,, \tag{8.18}$$
$$\widehat{\mathcal{A}}^{(4;0)}(\gamma_{1,B}) = -\mathcal{A}_t\,, \qquad \widehat{\mathcal{A}}^{(4;0)}(\gamma_{2,B}) = \mathcal{A}_t\,, \tag{8.19}$$
$$\widehat{\mathcal{A}}^{(4;0)}(\gamma_{3,A}) = \mathcal{A}_s\,, \qquad \widehat{\mathcal{A}}^{(4;0)}(\gamma_{3,B}) = -\mathcal{A}_t\,. \tag{8.20}$$

Here, we have denoted by $\mathcal{A}_s$ and $\mathcal{A}_t$ the $s$- and $t$-channel amplitudes, stripped of charge and colour factors (contrary to the conventions used so far), so that

$$\mathcal{M}_s \equiv \mathcal{A}_s^\star\,\mathcal{A}_s = \frac{t^2+u^2}{s^2}\,, \tag{8.21}$$
$$\mathcal{M}_t \equiv \mathcal{A}_t^\star\,\mathcal{A}_t = \frac{s^2+u^2}{t^2}\,, \tag{8.22}$$
$$\mathcal{M}_{st} \equiv \Re\left[\mathcal{A}_s^\star\,\mathcal{A}_t\right] = \frac{u^2}{st}\,. \tag{8.23}$$

There are three tree-level LO matrix elements, understood to be multiplied by the factor $\alpha_S^n\alpha^m$ with $n+m=2$, which we denote by

$$\mathcal{M}^{(4;0)}_{(n,m)}\,. \tag{8.24}$$

In order to compute them, it is sufficient to apply eq. (5.2), with the sums over flows determined by the specific contribution we need to derive. Explicitly

$$\mathcal{M}^{(4;0)}_{(2,0)} = \sum_{i,j=1}^{2}\sum_{a,b=A,B}\widehat{\mathcal{A}}^{(4;0)}(\gamma_{i,a})^\star\,\widehat{\mathcal{A}}^{(4;0)}(\gamma_{j,b})\,N^{-\rho(\gamma_{i,a})-\rho(\gamma_{j,b})}\,N^{\left|\mathcal{L}(\gamma_{i,a},\gamma_{j,b})\right|}\,, \tag{8.25}$$
$$\mathcal{M}^{(4;0)}_{(1,1)} = \sum_{i=1}^{2}\sum_{a,b=A,B}\widehat{\mathcal{A}}^{(4;0)}(\gamma_{i,a})^\star\,\widehat{\mathcal{A}}^{(4;0)}(\gamma_{3,b})\,N^{-\rho(\gamma_{i,a})-\rho(\gamma_{3,b})}\,N^{\left|\mathcal{L}(\gamma_{i,a},\gamma_{3,b})\right|}+\text{c.c.}\,, \tag{8.26}$$
$$\mathcal{M}^{(4;0)}_{(0,2)} = \sum_{a,b=A,B}\widehat{\mathcal{A}}^{(4;0)}(\gamma_{3,a})^\star\,\widehat{\mathcal{A}}^{(4;0)}(\gamma_{3,b})\,N^{-\rho(\gamma_{3,a})-\rho(\gamma_{3,b})}\,N^{\left|\mathcal{L}(\gamma_{3,a},\gamma_{3,b})\right|}\,, \tag{8.27}$$

so that

$$\mathcal{M}^{(4;0)}_{(2,0)} = \left(N^2-1\right)\left(\mathcal{M}_s+\mathcal{M}_t+\frac{2}{N}\mathcal{M}_{st}\right), \tag{8.28}$$
$$\mathcal{M}^{(4;0)}_{(1,1)} = -4\left(N^2-1\right)\mathcal{M}_{st}\,, \tag{8.29}$$
$$\mathcal{M}^{(4;0)}_{(0,2)} = N^2\left(\mathcal{M}_s+\mathcal{M}_t-\frac{2}{N}\mathcal{M}_{st}\right), \tag{8.30}$$

which can be easily verified to agree with the results obtained by means of standard computational techniques. As far as the soft-gluon limits are concerned, we need to employ

JHEP11(2021)045

eq. (6.4), with the colour flows summed as in eqs. (8.25)–(8.27). We obtain

$$\begin{aligned}\mathcal{M}^{(4;1)}_{(3,0)\text{SOFT}} = 2C_F\Bigg\{ & \Big[(N^2-1)\Big(\Big[-1,-4\Big]+\Big[-2,-3\Big]\Big)+\Xi\Big]\mathcal{M}_s \\ & +\Big[(N^2-1)\Big(\Big[-1,-3\Big]+\Big[-2,-4\Big]\Big)+\Xi\Big]\mathcal{M}_t \\ & +\frac{2}{N}\Big[(N^2-1)\Big(\Big[-1,-2\Big]+\Big[-3,-4\Big]\Big)+\Xi\Big]\mathcal{M}_{st}\Bigg\},\end{aligned} \tag{8.31}$$

$$\begin{aligned}\mathcal{M}^{(4;1)}_{(2,1)\text{SOFT}} = 4C_F\Bigg\{ & N\Big(\Big[-1,-4\Big]+\Big[-2,-3\Big]-\Big[-1,-2\Big]-\Big[-3,-4\Big]\Big)\mathcal{M}_s \\ & +N\Big(\Big[-1,-3\Big]+\Big[-2,-4\Big]-\Big[-1,-2\Big]-\Big[-3,-4\Big]\Big)\mathcal{M}_t \\ & -\Big[N^2\Big(\Big[-1,-3\Big]+\Big[-2,-4\Big]+\Big[-1,-4\Big]+\Big[-2,-3\Big]\Big)+2\Omega\Big]\mathcal{M}_{st}\Bigg\},\end{aligned} \tag{8.32}$$

$$\begin{aligned}\mathcal{M}^{(4;1)}_{(1,2)\text{SOFT}} = 2C_F\Bigg\{ & N^2\Big(\Big[-1,-3\Big]+\Big[-2,-4\Big]\Big)\mathcal{M}_s \\ & +N^2\Big(\Big[-1,-4\Big]+\Big[-2,-3\Big]\Big)\mathcal{M}_t \\ & +2N\Omega\mathcal{M}_{st}\Bigg\},\end{aligned} \tag{8.33}$$

having defined

$$\begin{aligned}\Omega = & \Big[-1,-2\Big]+\Big[-3,-4\Big]-\Big[-1,-3\Big] \\ & -\Big[-1,-4\Big]-\Big[-2,-3\Big]-\Big[-2,-4\Big],\end{aligned} \tag{8.34}$$

$$\Xi = \Big[-1,-2\Big]+\Big[-3,-4\Big]+\Omega\,. \tag{8.35}$$

As for the LO matrix elements, one can verify that the results of eqs. (8.31)–(8.33) coincide with those obtained with standard techniques.

It is instructive to compare eq. (8.29) with eqs. (8.28) and (8.30). In all three cases, the leading-$N$ contributions stem from a closed flow in which the L- and R-flows coincide (bearing in mind the identifications in eqs. (8.4)–(8.7)). Such contributions would constitute the building blocks for the determination of the shower initial conditions in a leading-$N$ PSMC, e.g. according to the prescription of ref. [37]. But while for eqs. (8.28) and (8.30) the relevant kinematical quantities are "squared" matrix elements (either $\mathcal{M}_s$ or $\mathcal{M}_t$), for eq. (8.29) what is relevant is the interference between the $s$- and $t$-channel amplitudes ($\mathcal{M}_{st}$). This shows that, even at leading $N$, when the matrix elements that underpin the hard events that initiate the QCD showers do not have the maximal power of $\alpha_S$ one must consider objects that are not necessarily positive definite. What is important is that the nature of such objects is determined by closed colour flows, and not by their kinematical characteristics. Direct evidence of this fact can be seen in the comparison of eqs. (8.28)–(8.30) with eqs. (8.31)–(8.33): the hierarchy in $N$ of the radiation patterns in

JHEP11(2021)045

the latter equations is reflected in that of the contributions to the former ones, and the correspondence is driven by the underlying closed colour flows.

We conclude this section by considering soft-photon emission patterns. This case is significantly simpler than its soft-gluon counterpart, owing to the fact that the charge-linked Borns (i.e. the matrix elements that multiply suitable linear combinations of eikonals) are all proportional to the LO tree-level matrix elements. As far as the linear combinations of eikonals are concerned, they are entirely determined by the charges of external partons that participate in the hard process. Thus, from eq. (3.12) of ref. [32], one defines the photon radiation pattern, i.e. the QED analogue of eq. (6.12)

$$\mathcal{E}_{\rm QED} = \sum_{k<l} e_k\, e_l \left[k, l\right], \tag{8.36}$$

with $e_i$ the charge of parton $i$, the sums are extended to all external partons, and, in keeping with the rest of this paper, all partons are outgoing. With this, the analogues of eqs. (8.31)–(8.33) read

$$\mathcal{M}^{(4;1)\gamma}_{(n,m+1){\rm SOFT}} = \left(\frac{2}{3}\right)^2 \Omega\, \mathcal{M}^{(4;0)}_{(n,m)}\,, \tag{8.37}$$

since for the process of eq. (8.1), the QED radiation pattern of eq. (8.36) coincides with eq. (8.34) up to an overall factor equal to the charge of the $u$ quark squared.

The fact that on the r.h.s. of eq. (8.36) one sums over all pairs of external partons implies that this radiation pattern is quite similar to a soft-gluon one relevant to a closed flow with a single colour loop that includes all the partons of the process. This is remarkable, because it puts QED and QCD radiation on the same footing, and allows one to treat the former in the same way as (one of the cases of) the latter. However, in general the two patterns are not identical, since the eikonals in $\mathcal{E}_{\rm QED}$ have coefficients that, in absolute value, are not all equal to each other, which does not happen in QCD. While this entails only minor modifications to the angular-ordered showering approach outlined in section 7.1.1, it is potentially more problematic for dipole showering, since the equivalence of the radiation pattern of eq. (8.36) and dipole graphs must be re-considered.

## 9 Conclusions

In this paper we have investigated the role that colour flows play in both the calculation of tree-level matrix elements and of their soft limits, and in the inclusion of effects beyond leading colour in parton shower Monte Carlos.

For these applications, and in general for any kind of non-approximate approach, it is appropriate to work with what we have called closed colour flows (section 2), which generalize the colour flows usually employed in leading-$N$ computations (which are therefore more appropriately referred to as open colour flows). In essence, closed colour flows are based on a picture that features a double copy of the colour/anticolour labels of the partons that participate in the process (one copy for each side of the Cutkosky cut; each such copy is an open colour flow). Leading-$N$ contributions are (possibly a subset of) those for which the orderings of the labels in the two open flows that form a closed flow are identical to each other.

Colour loops, which we have defined algorithmically in section 3, give a representation of closed colour flows that can be conveniently used in matrix element computations and PSMCs alike. By means of colour loops, one generalizes the concept of colour connection that is used at the leading $N$ (see in particular section 6.4). We have discussed how colour loops can in turn be equivalently described in terms of dipole graphs (section 7), which are an alternative to the former in PSMC implementations. Interestingly, the counting of the numbers of dipole graphs gives rise to integer sequences that, to the best of our knowledge, are presented here for the first time (sections 7.2.1 and 7.2.2), and for which we give generating functions.

In the case of purely gluonic processes, the colour loops as defined in this paper are seen (appendix A) to correspond to quantities emerging from the mathematics of permutations (in particular, the alternating cycles of a cycle graph). This is interesting, because it could pave the way to exploiting results that are increasingly important in other branches of science (mainly genome studies — the curious reader can find a list of classical mathematics papers, relevant to biology, for example in chapter 9 of ref. [45]).

While when working with colour loops each colour or anticolour label may have as many as $m-1$ colour partners ($m$ being the number of partons of the process), dipole graphs are more similar to the colour connections one is used to at the leading $N$, in that they feature a single partner per (anti)quark, and two partners per gluon. Furthermore, while colour loops require a double copy of the partons (as the closed colour flows from which they stem), dipole graphs need a single copy, and thus are topologically identical to any set of colour connections employed in existing PSMCs. At variance with the latter, however, dipole graphs feature colour-colour and anticolour-anticolour connections, in addition to the usual colour-anticolour ones. We stress again that, in spite of their topological differences, colour loops and dipole graphs give equivalent representations of the same thing. The simpler topological structure of the latter is compensated by the necessity of working with more complicated objects at the level of amplitudes squared.

Regardless of their possible use in the context of PSMCs, colour flows give an organising principle for the computation of tree-level matrix elements (section 5). We have shown that the treatment of any process is in fact no more complicated than that of one featuring only quarks and antiquarks, thanks to the introduction of the idea of secondary flows (section 5.2); these allow one to express the results for processes that include gluons in terms of the formulae relevant to the no-gluon case.

Although from tree-level matrix elements results it might seem that secondary flows are a computational device, the results for the soft limits (section 6) show that they are in fact more fundamental objects, in that they control the soft-radiation patterns with a clear hierarchy in $N$. Furthermore, through secondary flows one recovers a fundamental property of the leading-$N$ PSMCs, namely the fact that the radiation pattern of the first emission is entirely controlled by Born-level quantities. This property, which is trivially true at leading $N$ but not beyond it, allows one to incorporate subleading-$N$ effects in PSMCs in a Markovian way.

JHEP11(2021)045

For matrix element computations we often make use of vector spaces (that represent colour degrees of freedom), which are separable into spaces relevant to individual partons (section 4). In the presence of gluons, we have shown how the so-called fundamental and flow representations are associated with vectors in an $(N^2-1)$- and $N^2$-dimensional space respectively for each gluon, the former of which can be seen as a subspace of the latter, and whose complement in the latter is interpreted as representative of the degrees of freedom of U(1) gluons.

Matrix elements for processes that feature both quarks and gluons can be written as the incoherent sum of non-null contributions associated with different sets of U(1) gluons, in the same way as for amplitude-level quantities (see in particular eqs. (5.46) and (6.11)). In turn, these sets are associated with non-overlapping sets of secondary flows, thereby confirming the physical nature of the latter. U(1) gluons do appear also in purely-gluonic processes but, as is well known, they do not contribute to the cross section. However, this is true only after summing over all possible flows; conversely, if one restricts oneself to a subset of the flows, U(1)-gluon contributions are generally different from zero.

In section 7 we have discussed possible applications of colour flows to parton shower Monte Carlo simulations. In particular, we have proposed ways in which the subleading colour structure of Born matrix elements could be included in the matching of hard processes to parton showers. One possible approach, based on eq. (6.11), is to select closed flows according to their relative contributions to the Born process and then use the radiation pattern of each flow to influence the evolution of the associated showers. This could be done by using a modified Sudakov factor that reflects the sequence of scales associated with the colour connections of the evolving partons in the selected flow. In the case of an angular-ordered shower, the eikonal terms in the radiation pattern of the flow define a sequence of angular regions for the evolution of each participating parton, the rate of evolution in each region being controlled by the relevant colour-linked Born matrix elements.

Another possible Monte Carlo approach is more suited to the evolution of dipole showers, being based on dipole graphs and eq. (7.90) rather than directly on colour flows. We showed that each flow can be represented by a linear combination of dipoles, which can serve as initiators of dipole showers.

An issue that will affect the implementation of any scheme for including subleading colour contributions is that these can have either sign, leading to negative Monte Carlo weights. There are established methods for handling such weights [38–43]; which of them is most suitable in this case will probably depend on the process under study. A less familiar issue that we have highlighted is the appearance of self-connected gluons in both the single-parton and dipole implementations. Rather than treating these as non-showering objects that would give rise to isolated hard hadrons, we have suggested interpreting them as corrections to gluon jets generated by more leading contributions from the same momentum configuration of the Born process. In this approach, each Born configuration would be used to generate several "subevents" corresponding to different flows or dipole graphs.

The inclusion of subleading colour contributions via subevents, based on either colour flows or dipole graphs, will require some modification of hadronization models, since, as

JHEP11(2021)045

we have seen, the concept of colour partners is generalized to include quark-quark and antiquark-antiquark pairs, as well as gluons that connect to two quarks or two antiquarks. However, since colour is conserved overall, it is always possible to hadronize after allowing some colour reconnection among showers within each subevent.

We have also briefly discussed (section 8) how the techniques developed here in QCD can be applied with minor or no changes to the case where two perturbative couplings are relevant. Presently, the most important application of this is in the context of QCD+QED computations and, for matrix elements that do not have a maximal power of $\alpha_S$, it affects PSMC simulations even at the leading $N$.

We have usually presented our results both at fixed (Born-level) flows and after summing over flows. While only flow-summed predictions are physical, it is not a foregone conclusion that they must be obtained by means of analytically-summed formulae. In particular, when the number of partons involved becomes large, a random sum might be the only viable option, and this can be carried out only by using fixed-flow expressions. We point out that, even in the context of an MC@NLO matching [46], where a local connection between the short-distance cross sections and the PSMC must be established, there is no loss of efficiency, if suitable flow-based NLO-subtraction formulae are employed, e.g. as proposed in ref. [23].[17] Conversely, a random sum behaves in any case poorly from a statistics viewpoint unless one is capable of understanding *a priori* which flows give the dominant contributions in $N$ (or, more in general, some selected $N^k$ contributions), and of generating them with high efficiency. While the generation of flows is a topic not directly relevant to this paper, since flows are assumed to be given here, it may become important for the application of our results to multi-parton processes, and it is well worth pursuing.

## Acknowledgments

SF is grateful to Fabio Maltoni for collaborating during the early stages of this work, and for the endless and very entertaining brainstorming sessions during one of the Covid-19 lockdowns. Conversations with Stefan Prestel are also gratefully acknowledged. BRW thanks members of the Cambridge Pheno Working Group for valuable comments. This work was partially supported by STFC Consolidated HEP grants ST/P000681/1 and ST/T000694/1.

## A On gluon-only colour flows and colour factors

In this appendix we discuss some properties of colour flows relevant to gluon-only processes, and their associated colour factors. Where necessary, we shall exploit the following property:

$$C\left(\sigma', \sigma\right) = C\left(I, \sigma'^{-1} \circ \sigma\right) = C\left(\sigma^{-1} \circ \sigma', I\right) \tag{A.1}$$

of the colour-flow matrix element (see eqs. (2.34) and (5.53))

$$C(\sigma', \sigma) = \sum_{\{a_i\}_{i=1}^n} \mathrm{Tr}\left(\lambda^{a_{\sigma'(1)}} \ldots \lambda^{a_{\sigma'(n)}}\right)^\star \mathrm{Tr}\left(\lambda^{a_{\sigma(1)}} \ldots \lambda^{a_{\sigma(n)}}\right). \tag{A.2}$$

[17] A flow-based representation must also be chosen for the finite virtual contributions, the discussion of which was not within the scope of ref. [23].

JHEP11(2021)045

In eq. (A.1), by the symbol $\circ$ we have denoted the composition of permutations, to be applied from right to left, i.e.:

$$\Big(\sigma_A \circ \sigma_B\Big)(i) = \sigma_A\left(\sigma_B(i)\right) \,, \tag{A.3}$$

for any two permutations $\sigma_A$ and $\sigma_B$.

### A.1 Colour flows

A gluon-only closed flow $(\sigma', \sigma)$ has an interesting property. Define the permutation

$$\varsigma(\sigma', \sigma) = \sigma^{-1} \circ \sigma'_{\rightarrow} \circ \sigma'^{-1} \circ \sigma_{\leftarrow} \,, \tag{A.4}$$

where the subscripts $\rightarrow$ and $\leftarrow$ indicate that the corresponding permutation has to be rotated by one position to the right and to the left, respectively, i.e.

$$\sigma_A = \Big(\sigma_A(1), \sigma_A(2), \ldots \sigma_A(n)\Big) \tag{A.5}$$

$$\Longrightarrow \quad \sigma_{A\rightarrow} = \Big(\sigma_A(n), \sigma_A(1), \ldots \sigma_A(n-1)\Big) \,, \tag{A.6}$$

$$\sigma_{A\leftarrow} = \Big(\sigma_A(2), \sigma_A(3), \ldots \sigma_A(1)\Big) \,. \tag{A.7}$$

The definition in eq. (A.4) is motivated by what has been presented in section 3; more precisely, the four permutations on the r.h.s. of eq. (A.4) correspond, from right to left, to items 2, 3, 4, and 5 in the definition of colour loops. With this, one obtains

$$|\mathcal{L}(\sigma', \sigma)| = \Big|\text{cyc}\Big[\varsigma(\sigma', \sigma)\Big]\Big| \,. \tag{A.8}$$

That is, the number of colour loops in the closed flow $(\sigma', \sigma)$ is equal to the number of ordinary cycles[18] of the permutation $\varsigma(\sigma', \sigma)$ defined in eq. (A.4). Equation (A.8) stems from a more fundamental property, which is the following. For any colour loop

$$\ell = (\sigma(i_1), \sigma'(j_1), \sigma(i_2), \ldots) \in \mathcal{L} \,, \tag{A.9}$$

then

$$\exists!\, c \in \text{cyc}\left[\varsigma\left(\sigma', \sigma\right)\right] \quad \text{s.t.} \quad c = (i_1, i_2, \ldots) \,. \tag{A.10}$$

That is: for a given closed flow, each colour loop is in a one-to-one correspondence with a cycle of the permutation defined in eq. (A.4). Furthermore, the labels of such a cycle are mapped onto the labels of the R-elements of the corresponding colour loop by means of the R-flow permutation.

By establishing a direct connection between colour loops and ordinary cycles of permutations (which are a much better studied subject) these results are helpful in that they give an alternative viewpoint on colour loops. Note that eq. (A.8) is manifestly invariant under the operations on the r.h.s. of eq. (A.1).

[18]Here, the cycles of a permutation include explicitly all of its 1-cycles; these are sometimes understood in the mathematics literature.

JHEP11(2021)045

There is a further remarkable fact: the idea of colour loops, and in particular their construction given in steps 0–7 of section 3, is essentially identical to that of *alternating cycles*, which (as far as we know) has been introduced in ref. [47] in the context of the mathematics of permutations, and which is nowadays a powerful tool in the study of genome re-arrangements; a good summary can be found e.g. in chap. 9 of ref. [45]. The essence is that, given a permutation $p$, one constructs a double-edged graph, denoted by $G(p)$ and called the *cycle graph* of $p$, which connects the various elements of $p$. Such connections are represented as ordered sets of elements, called the alternating cycles of $p$, whose number is usually denoted by $c(G(p))$. The interested reader can easily verify that the rules to construct a cycle graph and its alternating cycles are very closed related to the rules given in section 3. An immediate consequence is therefore[19]

$$\mathcal{L}\left(\sigma, I\right) = c\left(G(p)\right) . \tag{A.11}$$

It is intuitively clear that this equation leads to eq. (A.8); in fact, we shall show below that the analogy is even stricter.

The advantage of being able to establish a direct connection between colour loops and alternating cycles lies in the ability to exploit the growing (thanks to the interest in biology) number of mathematics results relevant to the latter. A first example is that of counting. From definition 9.12 of ref. [45]:

- The number of $n$-element permutations with $k$ alternating cycles is the Hultman number [48] $\mathcal{S}_{\rm H}\left(n, k\right)$.

Therefore, thanks to eq. (A.11), we have

$$\sum_{\sigma\in P_n'} 1\cdot\delta\Big(k, |\mathcal{L}(\sigma, I)|\Big) = \mathcal{S}_{\rm H}\left(n-1, k\right) , \qquad 1 \le k \le n . \tag{A.12}$$

In other words, the number of closed colour flows that correspond to a given number ($k$) of $n$-gluon colour loops is equal to the Hultman number, computed at $(n-1, k)$. Fortunately, Hultman numbers are known in a closed form [49]

$$\mathcal{S}_{\rm H}\left(n, k\right) = \begin{bmatrix} n+2 \\ k \end{bmatrix} \Big/ \begin{pmatrix} n+2 \\ 2 \end{pmatrix} , \qquad n-k \text{ odd} , \tag{A.13}$$

$$\mathcal{S}_{\rm H}\left(n, k\right) = 0 , \qquad n-k \text{ even} , \tag{A.14}$$

where $[n\ k]$ is the unsigned Stirling number of the first kind, which counts the number of $n$-element permutations with $k$ ordinary cycles. Note that from eq. (A.12) one must have

$$\sum_{k=1}^{n} \mathcal{S}_{\rm H}\left(n-1, k\right) = (n-1)! , \tag{A.15}$$

[19] Theorems on permutations usually include cyclic ones. Since any non-cyclic $n$-element permutation (say, $\sigma$) is in one-to-one correspondence with an $(n-1)$-element permutations (say, $p$), a relabelling (e.g. $\sigma(i) = p(i-1)$, with $2 \le i \le n$), plus the insertion of a conventional fixed point (e.g. $\sigma(1) = 1$), allows one to straightforwardly apply the results of such theorems to colour flows. This is understood in eq. (A.11), and is the reason for the $n-1$ argument on the r.h.s. of eq. (A.12).

JHEP11(2021)045

which can indeed be found with eqs. (A.13) and (A.14). Note, also, that eqs. (A.12) and (A.14) confirm that the number of colour loops for any given flow has the same parity as the number of gluons.

A theorem by Doignon and Labarre [49] (specifically, theorem 8 there) is directly relevant to the counting of colour loops. It reads as follows:

- Let[20] $\alpha = (0 \ldots n-1)$. The mapping

$$F: \quad \Big\{\pi \in S_{n-1} | c(G(\pi)) = k\Big\} \longrightarrow \tag{A.16}$$
$$\Big\{\sigma \in S(n) | c(\Gamma(\sigma)) = k \& \exists \rho \in S(n) : c(\Gamma(\rho)) = 1 \& \alpha = \rho \circ \sigma\Big\}$$

$$F: \quad \pi \longmapsto \mathring{\pi} \tag{A.17}$$

is bijective.

As is customary, for the map $F$ one introduces the domain and codomain (eq. (A.16)) and a symbol ($\mathring{\pi} \equiv F(\pi)$) for its result when acting on a specific element ($\pi$) of the domain (eq. (A.17)). As before, $c(G(\pi))$ is the number of alternating cycles of $\pi$, while $c(\Gamma(\rho))$ is the number of ordinary cycles of $\rho$. $S_{n-1}$ is the set of $n$-object permutations; $S(n)$ is the set of factorizations of a given $n$-cycle (in this case, $\alpha$) into some $n$-cycle ($\rho$) and some permutation ($\sigma$). By taking into account the different labelling conventions of ref. [49] and of the present paper, and by observing that

$$I_{\leftarrow} = \alpha \,(\,[49]) \,, \tag{A.18}$$

the relationships between the quantities of eqs. (A.16) and (A.17) and those introduced here relevant to $\mathcal{L}(\sigma, I)$ are the following (see footnote 19):

$$\sigma \longleftrightarrow \pi \,(\,[49]) \,, \tag{A.19}$$

$$\varsigma(\sigma, I) \longleftrightarrow \sigma \,, \mathring{\pi} \,(\,[49]) \,, \tag{A.20}$$

$$\Big(\sigma_{\rightarrow} \circ \sigma^{-1}\Big)^{-1} \longleftrightarrow \rho \,(\,[49]) \,. \tag{A.21}$$

The bijective nature of the map $F$ and eq. (A.11) give a formal proof of eq. (A.8) (taking the property of eq. (A.1) into account).

We point out that ref. [49] need only introduce $\rho$, and not the combination on the l.h.s. of eq. (A.21), which in our case stems directly from the construction of colour loops. The consistency of the two procedures follows from the following fact:

- For any given $n$-element permutation $\sigma$, the quantity $\sigma_{\rightarrow} \circ \sigma^{-1}$ is an $n$-cycle.

*Proof:* first observe that cycles must be invariant under a general index relabelling; in other words, label indices by $\sigma(i)$ rather than by $i$. Then[21]

$$\sigma_{\rightarrow} \circ \sigma^{-1}(\sigma(i)) = \sigma(i \ominus 1) \,, \tag{A.22}$$

[20] As is standard in the mathematics of permutations, $(\ldots)$ is the cycle notation. Furthermore, ref. [49] labels elements starting from zero, as opposed to one as is done here.

[21] The $\oplus$ and $\ominus$ symbols are normal addition and subtraction operators, augmented by a cyclicity condition: $n \oplus 1 = 1$ and $1 \ominus 1 = n$.

JHEP11(2021)045

whence

$$\left(\sigma_{\rightarrow} \circ \sigma^{-1}\right)^{k} (\sigma(i)) = \sigma(i \ominus k)\,. \tag{A.23}$$

Thus

$$\left(\sigma_{\rightarrow} \circ \sigma^{-1}\right)^{k} (\sigma(i)) = \sigma(i) \quad \Longleftrightarrow \quad k = n \qquad \forall\, i\,, \tag{A.24}$$

which concludes the proof.

We finally quote two theorems relevant to permutations that could be useful in dealing with closed colour flows. The first is reported as theorem 9.9 of ref. [45], and stems from ref. [50]. It uses the concept of *block interchange distance* $\mathrm{bid}(\sigma)$ for a given permutation $\sigma$, defined as the smallest number of block interchanges that sort $\sigma$ (i.e. transform $\sigma$ into the identity). That theorem states that (in the language of this paper)

$$\mathrm{bid}(\sigma) = \frac{n - |\mathcal{L}(\sigma, I)|}{2}\,. \tag{A.25}$$

This gives an interesting geometric interpretation to the number of colour loops which, as is explicit in eq. (A.25), decreases linearly with the block interchange distance.[22]

The second theorem alluded to before is reported as theorem 9.22 of ref. [45]. Again using the language of this paper, it states that

$$\langle |\mathcal{L}| \rangle = \left( \left\lfloor \frac{n+1}{2} \right\rfloor \right)^{-1} + H_1(n-1)\,, \tag{A.26}$$

with $H_1$ the first harmonic number, and the average on the l.h.s. is computed over all $n$-gluon flows. From eq. (A.12) it then follows that

$$\left( \left\lfloor \frac{n+1}{2} \right\rfloor \right)^{-1} + H_1(n-1) = \frac{1}{(n-1)!} \sum_{k=1}^{n} k\, \mathcal{S}_{\mathrm{H}}\,(n-1, k)\;. \tag{A.27}$$

Furthermore, eq. (A.26) implies

$$\langle |\mathcal{L}| \rangle \quad \stackrel{n\to\infty}{\longrightarrow} \quad \log n\,. \tag{A.28}$$

Since $\max(|\mathcal{L}|) = n$, eq. (A.28) tells one that the larger $n$, the more unlikely that a flat random generation results in a flow with a large number of colour loops. Conversely, a flat random generation should be relatively efficient in producing highly-suppressed colour contributions.

## A.2 Colour factors

We now turn to the discussion of some features of the colour factors. Firstly, the colour-flow matrix elements of eq. (A.2) on the diagonal (i.e. $\sigma' = \sigma$) can be easily computed by means of secondary flows, by employing eqs. (5.54) and (5.60) since, for any $n$-gluon colour flow $\sigma$, we have

$$|\mathcal{L}(\bar\sigma_{q\mathrm{L}\not{s}_k}, \bar\sigma_{q\mathrm{R}\not{s}_k})| = \begin{cases} n-k & \quad 0 \le k \le n-1 \\ 2 & \quad k = n \end{cases} \qquad \forall\, s_k \in S_k^{(n)}\,. \tag{A.29}$$

[22]The block interchange distance can be defined between two permutations, exploiting the same property as in eq. (A.1). Therefore, eq. (A.25) holds in the case of a generic closed colour flow, as it should for consistency.

JHEP11(2021)045

Thus

$$C(\sigma,\sigma) = \sum_{k=0}^{n} \frac{(-1)^k}{2^n} \binom{n}{k} N^{-k} \left[\Theta(k \le n-1)\, N^{n-k} + \delta_{kn} N^2\right]$$
$$= \frac{1}{2^n N^n} (N^2-1) \left((N^2-1)^{n-1} + (-1)^n\right). \tag{A.30}$$

The rightmost side of eq. (A.30) exhibits the usual factor

$$f_N = N^2 - 1\,. \tag{A.31}$$

On top of that, it is easy to show analytically for any $n$ that

$$C(\sigma,\sigma) = (N^2-1)\,(N^2-2)\,q(N)/N^n \quad \Longleftrightarrow \quad n \text{ odd}\,, \tag{A.32}$$

for some polynomial $q(N)$. We stress that while the factorization of $f_N$ occurs also for off-diagonal elements, this is *not* the case for the factor $N^2 - 2$.

As is the case for the diagonal colour-flow matrix elements, for the off-diagonal ones the emergence of the factor $f_N$ is not immediately apparent, the r.h.s. of eq. (5.60) being a linear combinations of monomials $N^p$. It is the contributions of the U(1) gluons that add and subtract suitable terms to the leading $N^n$ monomial, so that $f_N$ eventually appears. This poses an interesting question, namely: the matrix elements summed over flows, with the colour factors computed with traces of $\lambda$ matrices, manifestly factor out $f_N$ because each of the products of two traces of $\lambda$ does so. On the other hand, as has been shown in eq. (5.66), such matrix elements can be computed by simply counting colour loops, and by ignoring U(1)-gluon contributions. Therefore, in view of the fact that it is those contributions that cause the emergence of the $f_N$ factor *prior to summing* over flows, where does $f_N$ come from if eq. (5.66) is employed?

In order to answer that question, we re-write eq. (5.66) more compactly as follows:

$$\mathcal{M}^{(n)} = \frac{1}{2^n} \sum_{i,j=1}^{(n-1)!} \mathcal{M}_{ij}^{(n)}\,, \tag{A.33}$$
$$\mathcal{M}_{ij}^{(n)} = N^{|\mathcal{L}_{ij}|}\, \widehat{\mathcal{A}}_i^{(n)\star}\, \widehat{\mathcal{A}}_j^{(n)}\,, \tag{A.34}$$

i.e. we temporarily denote the flows by integer indices. Next, consider one of the contributions to eq. (A.33) stemming from summing over columns after choosing a row — to be definite, take the $i = 1$ row

$$X = \frac{1}{\widehat{\mathcal{A}}_1^{(n)\star}} \sum_{j=1}^{(n-1)!} \mathcal{M}_{1j}^{(n)} = \sum_{j=1}^{(n-1)!} N^{|\mathcal{L}_{1j}|}\, \widehat{\mathcal{A}}_j^{(n)}\,. \tag{A.35}$$

In view of eq. (A.12) and of the properties of the Hultman numbers in eqs. (A.13) and (A.14), eq. (A.35) can be re-cast as follows:

$$X = \sum_{k=n}^{k_{\min}}{}^{\bigstar}\; N^k \sum_{j=\mathcal{T}_{\rm H}(n-1,k+2)+1}^{\mathcal{T}_{\rm H}(n-1,k)} \widehat{\mathcal{A}}_j^{(n)}\,, \tag{A.36}$$

JHEP11(2021)045

generally after a relabelling of the flows, so that those with the same number of colour loops are contiguous. The ★ symbol restricts the sum over $k$ to run over values whose parity is that same as that of $n$. In eq. (A.36) we have used the sequence $\mathcal{T}_{\rm H}\,(n,k)$ that is monotonically increasing for decreasing $k$

$$\mathcal{T}_{\rm H}\,(n,k) = \sum_{j=n+1}^{k} \mathcal{S}_{\rm H}\,(n,j)\,, \qquad 1 \le k \le n+1\,, \tag{A.37}$$

$$\mathcal{T}_{\rm H}\,(n,k) = 0\,, \qquad k \ge n+2\,, \tag{A.38}$$

and

$$k_{\rm min} = 2 - {\rm mod}(n,2)\,. \tag{A.39}$$

Note that, thanks to eqs. (A.14) and (A.15)

$$\mathcal{T}_{\rm H}\,(n-1,k_{\rm min}) = (n-1)!\,, \tag{A.40}$$

so that the sums on the r.h.s.'s of eqs. (A.35) and (A.36) indeed contain the same number of terms. Because of the dual Ward identities, there are many ways to collect $(n-1)$ amplitudes whose sum is equal to zero. However, it is not guaranteed that the corresponding colour factors will all be equal to each other. In other words, for some $(n-1)$ flows labelled by $j_1 \ldots j_{n-1}$, there might not exist a value $k$ such that

$$j_1 \ldots j_{n-1} \;\in\; \Big[\mathcal{T}_{\rm H}\,(n-1,k+2)+1\,,\mathcal{T}_{\rm H}\,(n-1,k)\Big]\,. \tag{A.41}$$

Therefore, the dual Ward identity

$$\widehat{\mathcal{A}}_{j_1}^{(n)} + \ldots + \widehat{\mathcal{A}}_{j_{n-1}}^{(n)} = 0 \tag{A.42}$$

cannot be easily exploited in eq. (A.36). On the other hand, eq. (A.42) implies

$$\sum_{j=1}^{(n-1)!} \widehat{\mathcal{A}}_j^{(n)} = 0\,, \tag{A.43}$$

and therefore

$$\sum_{j=\mathcal{T}_{\rm H}(n-1,k_{\rm min}+2)+1}^{\mathcal{T}_{\rm H}(n-1,k_{\rm min})} \widehat{\mathcal{A}}_j^{(n)} = -\sum_{k=n}^{k_{\rm min}+2} \;\sum_{j=\mathcal{T}_{\rm H}(n-1,k+2)+1}^{\mathcal{T}_{\rm H}(n-1,k)} \widehat{\mathcal{A}}_j^{(n)}\,, \tag{A.44}$$

whence

$$X = \sum_{k=n}^{k_{\rm min}+2} \left(N^k - N^{k_{\rm min}}\right) \sum_{j=\mathcal{T}_{\rm H}(n-1,k+2)+1}^{\mathcal{T}_{\rm H}(n-1,k)} \widehat{\mathcal{A}}_j^{(n)}\,. \tag{A.45}$$

One finally exploits the fact that, for any $n$ and $k$, one can write

$$k \text{ even}: \qquad N^k - N^{k_{\rm min}} = N^2\left(N^{2p}-1\right)\,, \tag{A.46}$$

$$k \text{ odd}: \qquad N^k - N^{k_{\rm min}} = N\left(N^{2p}-1\right)\,. \tag{A.47}$$

JHEP11(2021)045

For any integer $p$

$$N^{2p} - 1 = \left(N^2 - 1\right) \sum_{m=0}^{p-1} N^{2m}\,, \tag{A.48}$$

which shows that $f_N$ indeed factors out of eq. (A.45), as was expected.

It is also easy to see that eq. (A.43) is not only a sufficient condition for the emergence of $f_N$, but also a necessary one. Indeed, if the polynomial $X$ of $N$ factors out $f_N$, then $X(N = 1) = 0$. Thus, from eq. (A.35)

$$X(N=1) = \sum_{j=1}^{(n-1)!} N^{|\mathcal{L}_{1j}|} \widehat{\mathcal{A}}_j^{(n)} \Bigg|_{N=1} = \sum_{j=1}^{(n-1)!} \widehat{\mathcal{A}}_j^{(n)} = 0\,, \tag{A.49}$$

which proves the statement above.

These arguments about $f_N$ allow us to point out that, while the fundamental and the flow representations are equivalent for flow-summed gluon-only quantities, this is not true in the intermediate steps of any computation. Whether this has any practical consequences depends on which role such intermediate steps play in attaining the final result. An example relevant to the case discussed here is the following: suppose one performs the sum over flows by means of Monte Carlo methods. The difference between the results obtained with the fundamental (where U(1)-gluon contributions are kept non-null) and the flow representation will vanish only in the infinite-statistics limit. At finite statistics, it is likely that the flow representation will induce a larger number of cancellations among different terms, because this is the only manner in which the factor $f_N$ can arise. In order to increase the efficiency, then, it is wise to set up a flow-representation computation in a way that incorporates the dual Ward identities exactly and in each step of the procedure.

## B Equivalence of closed colour flows and dipole graphs

In this appendix, we sketch the proof of the equivalence between closed colour flows and dipole graphs that underpins eq. (7.90). Although we are unable to prove formally this equivalence for any number of quarks and gluons, we show a possible way to do this. We stress that, in all the cases we have explicitly dealt with, the equivalence has been found to hold true. We also show that, should the equivalence break down for some values of $q$ and $n$, there is an alternative whereby one still employs eq. (7.90), by means of a minor extension of the definition of dipole graphs.

We start by observing that the radiation patterns of eq. (7.74) can be represented as a matrix, whose row and column indices are the positions of the elements in the colour loop, and whose entries are equal to either $+1$ or $-1$, according to the sign in front of the eikonal associated with those elements; the diagonal elements are set equal to zero (since they correspond to self eikonals). This matrix being symmetric, we limit ourselves to writing

JHEP11(2021)045

its upper right triangle. Therefore

$$
\begin{array}{c} \\ M(\mathcal{E}_\ell(\bar{\gamma}',\bar{\gamma})) = \end{array}
\begin{array}{c}
\begin{array}{cccccc} e_1 & e_2 & e_3 & \cdots & e_{2m_\ell-1} & e_{2m_\ell} \end{array} \\
\begin{pmatrix}
0 & +1 & -1 & \dots & -1 & +1 \\
 & 0 & +1 & \dots & +1 & -1 \\
 & & 0 & \dots & -1 & +1 \\
 & & & \ddots & \vdots & \vdots \\
 & & & & 0 & +1 \\
 & & & & & 0
\end{pmatrix}
\end{array}
\begin{array}{c} e_1 \\ e_2 \\ e_3 \\ \dots \\ e_{2m_\ell-1} \\ e_{2m_\ell} \end{array}
\tag{B.1}
$$

so that

$$
\mathcal{E}_\ell(\bar{\gamma}',\bar{\gamma}) = \frac{1}{2}\sum_{i,j=1}^{2m_\ell} M(\mathcal{E}_\ell(\bar{\gamma}',\bar{\gamma}))_{ij}\left[e_i, e_j\right] . \tag{B.2}
$$

Equation (B.1) shows that the two sums on the r.h.s. of eq. (7.74) coincide with those of the matrix elements with like signs on the northwest-southeast diagonals.

The basic idea is that of exploiting the matrix representation of eq. (B.1) for $\mathcal{E}^+$ and $\mathcal{E}^-$ separately, and to show that it can be written as a sum of matrices that are representations of dipole graphs, eq. (7.22).

In order to do so, we start from a case that features only quarks and antiquarks. Therefore, from eq. (7.79)

$$
2q = \sum_\ell 2m_\ell . \tag{B.3}
$$

The $(2q) \times (2q)$ matrices[23]

$$
M\left(\mathcal{E}^+\right) = M\left(\sum_\ell \mathcal{E}_\ell^+\right) , \qquad M(\mathcal{E}^-) = M\left(\sum_\ell \mathcal{E}_\ell^-\right) , \tag{B.4}
$$

are block matrices constructed by starting from the $(2m_\ell) \times (2m_\ell)$ matrices

$$
M\left(\mathcal{E}_\ell^+\right) , \qquad M\left(\mathcal{E}_\ell^-\right) , \tag{B.5}
$$

and by embedding them (as blocks) in a $(2q) \times (2q)$ null matrix, so that we can formally write

$$
M\left(\sum_\ell \mathcal{E}_\ell^+\right) = \sum_\ell M\left(\mathcal{E}_\ell^+\right) , \qquad M\left(\sum_\ell \mathcal{E}_\ell^-\right) = \sum_\ell M\left(\mathcal{E}_\ell^-\right) , \tag{B.6}
$$

This property stems from item iii. in section 3: for any given quark or antiquark, there exists a single colour loop that contains its (anti)colour label. Equation (B.6) allows one to work with an individual $M(\mathcal{E}_\ell^\pm)$ matrix. From eq. (B.1), we see that

$$
M\left(\mathcal{E}_\ell^+\right)_{ij} = \delta(1, \mathrm{mod}(i-j, 2)) , \tag{B.7}
$$

$$
M\left(\mathcal{E}_\ell^-\right)_{ij} = \delta(0, \mathrm{mod}(i-j, 2))\,(1-\delta_{ij}) , \tag{B.8}
$$

[23]Henceforth, in order to simplify the notation we often understand the closed flow $(\bar{\gamma}', \bar{\gamma})$, which is given and fixed.

so that, in keeping with eqs. (7.75) and (B.2)

$$M(\mathcal{E}_\ell)_{ij} = M\left(\mathcal{E}_\ell^+\right)_{ij} - M\left(\mathcal{E}_\ell^-\right)_{ij}\,, \tag{B.9}$$

and therefore

$$\mathcal{E}_\ell^\pm(\bar\gamma',\bar\gamma) = \frac{1}{2}\sum_{i,j=1}^{2m_\ell} M\left(\mathcal{E}_\ell^\pm(\bar\gamma',\bar\gamma)\right)_{ij}\left[e_i,e_j\right], \tag{B.10}$$

which are the analogues of eq. (B.2). Let us first consider the case of $\mathcal{E}_\ell^+$. We define the following $m_\ell$ matrices:

$$M\left(\mathcal{E}_{\ell,k}^+\right)_{ij} = \delta(j, 1+\mathrm{mod}(1-k-i, 2m_\ell))\,, \qquad 1\leq k\leq 2m_\ell-1\,, \qquad k \text{ odd}\,. \tag{B.11}$$

These matrices are symmetric, traceless, and have the following properties:

$$\sum_k M\left(\mathcal{E}_{\ell,k}^+\right) = M\left(\mathcal{E}_\ell^+\right)\,, \tag{B.12}$$

$$\sum_{i=1}^{2m_\ell} M\left(\mathcal{E}_{\ell,k}^+\right)_{ij} = \sum_{j=1}^{2m_\ell} M\left(\mathcal{E}_{\ell,k}^+\right)_{ij} = 1\,. \tag{B.13}$$

It should be clear, then, that each of the matrices defined in eq. (B.11) represents what in section 7.4 has been called a candidate subset of a dipole graph. This essentially concludes the argument, since each of the matrices $M(\mathcal{E}_{\ell,k}^+)$ can be transformed into a $(2q)\times(2q)$ block matrix as was done above, so that

$$M\left(\mathcal{E}^+\right) = \sum_\ell\sum_k M\left(\mathcal{E}_{\ell,k}^+\right) = \sum_{k_1,\ldots k_{|\mathcal{L}|}}\sum_\ell M\left(\mathcal{E}_{\ell,\{k_\ell\}}^+\right)\,. \tag{B.14}$$

In the rightmost side of this equation we have exchanged the order of the summations over the loops and the index that identifies the individual matrices introduced in eq. (B.11). In doing so, we need to have one such index for each block; in other words, a choice of the values of the indices $k_1,\ldots k_{|\mathcal{L}|}$ corresponds to choosing one of the matrices of eq. (B.11) for each of the blocks. In this way, for any given $k_1,\ldots k_{|\mathcal{L}|}$ set of values, the matrix

$$M\left(\mathcal{E}_{\{k_\ell\}}^+\right) := \sum_\ell M\left(\mathcal{E}_{\ell,\{k_\ell\}}^+\right) \tag{B.15}$$

is a matrix representative of a dipole graph. In summary, by replacing eq. (B.14) into (the positive-eikonal part of) eq. (B.2) one shows that the radiation pattern of $\mathcal{E}^+$ can be written as the sum of the radiation patterns of the dipoles $\mathcal{E}_{\{k_\ell\}}^+$. Thus, eq. (B.14) is the matrix form of eq. (7.82), and also proves that all the $c^+$ coefficients in the latter equation are equal to one.

It is important to stress again that not only is eq. (B.11) not expected to be unique, but also that the decomposition of the radiation pattern of a closed flow into its positive- and negative-definite components is not mandatory. Therefore, it is in general possible to achieve a representation of a closed flow in terms of dipoles, with the number of the latter

JHEP11(2021)045

smaller than those in eq. (7.80);[24] we shall give an explicit example of this fact later in this appendix. However, this is not important here, since what is crucial is that the above proves that there is *at least* a way in which this representation can be achieved.

Unfortunately, things are not as simple for the radiation patterns of eikonals with negative signs, eq. (B.8), since we have not been able to find matrices analogous to those of eq. (B.11) that have all the properties of the latter. We note in particular that, when $q$ is an odd number, the analogue of eq. (B.12) cannot be true: some contributions must have opposite signs, so as to achieve a cancellation. This is because the radiation pattern $\mathcal{E}^-$ arises necessarily from quark-quark and antiquark-antiquark eikonals (owing to the properties of colour loops, section 3), but never from quark-antiquark eikonals. For this to correspond to a set of dipole graphs fulfilling the analogue of eq. (B.12), each such graph would therefore contain $q/2$ pairs of quark indices and $q/2$ pairs of antiquark indices, which is impossible since $q/2$ is not in general an integer number.

Having said that, we observe that the $m_\ell - 1$ matrices

$$M\left(\mathcal{E}^-_{\ell,k}\right)_{ij} = \delta(j, 1 + \mathrm{mod}(i + k - 1, 2m_\ell))\,, \qquad 2 \le k \le 2m_\ell - 2\,, \qquad k \text{ even}\,, \tag{B.16}$$

are traceless and fulfill properties identical to those in eqs. (B.12) and (B.13), namely

$$\sum_k M\left(\mathcal{E}^-_{\ell,k}\right) = M\left(\mathcal{E}^-_\ell\right)\,, \tag{B.17}$$

$$\sum_{i=1}^{2m_\ell} M\left(\mathcal{E}^-_{\ell,k}\right)_{ij} = \sum_{j=1}^{2m_\ell} M\left(\mathcal{E}^-_{\ell,k}\right)_{ij} = 1\,, \tag{B.18}$$

but *not being symmetric* (except when $k = m_\ell$; thus, this happens only if $m_\ell$ is even) cannot be representative of (a subset of) any dipole graph. However, by means of eq. (B.16) we can construct the following set of $\lfloor m_\ell/2 \rfloor$ matrices:

$$M'\left(\mathcal{E}^-_{\ell,k}\right) = \frac{1}{1+\delta_{km_\ell}}\left(M\left(\mathcal{E}^-_{\ell,k}\right) + M\left(\mathcal{E}^-_{\ell,2m_\ell-k}\right)\right) \qquad 2 \le k \le m_\ell\,, \qquad k \text{ even}\,. \tag{B.19}$$

These matrices are traceless and symmetric, and obey

$$\sum_k M'\left(\mathcal{E}^-_{\ell,k}\right) = M'\left(\mathcal{E}^-_\ell\right)\,, \tag{B.20}$$

$$\sum_{i=1}^{2m_\ell} M'\left(\mathcal{E}^-_{\ell,k}\right)_{ij} = \sum_{j=1}^{2m_\ell} M'\left(\mathcal{E}^-_{\ell,k}\right)_{ij} = \delta_{km_\ell} + 2\left(1 - \delta_{km_\ell}\right)\,. \tag{B.21}$$

This implies that they can be used in a dipole-like representation of the $\mathcal{E}^-$ radiation pattern, provided that one extends the definition of dipole graphs to include the cases where each quark and each antiquark label appears in exactly two (as opposed to one) pairs. This corresponds to allowing, in a PSMC implementation, any quark and any antiquark to have two colour partners.

The procedure described so far cannot be directly applied in the presence of gluons, since a gluon may appear in two different loops, which renders invalid the construction of

[24] This will of course also change the individual values of the $c^\pm$ coefficients.

JHEP11(2021)045

the block matrices as was done thus far. However, one may treat the colour and anticolour labels of a gluon as a quark and an antiquark. One then proceeds as above and, at the very end, merges the two rows and columns relevant to each gluon (by summing their contents). The resulting rows and columns will sum to two, rather than to one (to four rather than to two in the case of the $M'(\mathcal{E}^-_{\ell,k})$ matrices), with the symmetry and traceless properties obviously unspoilt. One thus obtains again matrices that represent dipole graphs (or generalized dipole graphs, in the case of $\mathcal{E}^-$) that include gluons, *except* for the following important detail. By treating, during the construction, the colour and anticolour indices of a gluon as independent from each other, one obtains self-eikonals which are not on the diagonal (see e.g. eq. (B.23)). However, such a case is easy to diagnose, because after merging the rows and columns it results in an entry equal to two on the diagonal. When this happens, that gluon has to be removed, and thus interpreted as a self-connected gluon. After the removal, one is left with a dipole graph of dimension smaller than the original one, which is what has already been discussed in section 7.2.2.

**Example.** We consider the case of the closed flow $(\bar{\gamma}_{0,1}, \bar{\gamma}_{0,2})$ in table 1. Equation (B.1) reads (there is a single loop, so the index $\ell$ is omitted)

$$M(\mathcal{E}(\bar{\gamma}_{0,1}, \bar{\gamma}_{0,2})) = \begin{array}{c} \begin{matrix} -1 & \bar{2} & \bar{1} & -2 & \bar{2} & \bar{1} \end{matrix} \\ \begin{pmatrix} 0 & +1 & -1 & +1 & -1 & +1 \\ & 0 & +1 & -1 & +1 & -1 \\ & & 0 & +1 & -1 & +1 \\ & & & 0 & +1 & -1 \\ & & & & 0 & +1 \\ & & & & & 0 \end{pmatrix} \end{array} \begin{matrix} -1 \\ \bar{2} \\ \bar{1} \\ -2 \\ \bar{2} \\ \bar{1} \end{matrix} \tag{B.22}$$

and, equivalently

$$\begin{aligned} \mathcal{E}^+(\bar{\gamma}_{0,1}, \bar{\gamma}_{0,2}) = {} & \left[-1, \bar{2}\right] + \left[\bar{2}, \bar{1}\right] + \left[\bar{1}, -2\right] + \left[-2, \bar{2}\right] + \left[\bar{2}, \bar{1}\right] + \\ & \left[-1, -2\right] + \left[\bar{2}, \bar{2}\right] + \left[\bar{1}, \bar{1}\right] + \\ & \left[-1, \bar{1}\right], \end{aligned} \tag{B.23}$$

$$\begin{aligned} \mathcal{E}^-(\bar{\gamma}_{0,1}, \bar{\gamma}_{0,2}) = {} & \left[-1, \bar{1}\right] + \left[\bar{2}, -2\right] + \left[\bar{1}, \bar{2}\right] + \left[-2, \bar{1}\right] + \\ & \left[-1, \bar{2}\right] + \left[\bar{2}, \bar{1}\right]. \end{aligned} \tag{B.24}$$

The three lines of eq. (B.23) correspond to the three northwest-southeast diagonals (from left to right) with positive signs, and the two lines of eq. (B.24) to the two diagonals (from left to right) with negative signs, of the matrix in eq. (B.22). The sum of the r.h.s.'s of eqs. (B.23) and (B.24) is equal to the r.h.s. of eq. (6.23), as it should by construction. In this example, we have

$$q = 1\,, \quad n = 2\,, \quad m = 3\,, \quad n_{g^\star} = 2\,, \tag{B.25}$$

JHEP11(2021)045

and indeed, in keeping with eqs. (7.76) and (7.77),

$$m^2 = 9\,, \qquad m(m-1) = 6\,, \tag{B.26}$$

are the number of terms in eq. (B.23) and the number of terms in eq. (B.24), respectively.

Since $n_{g^\star} \neq 0$, we expect the radiation pattern $\mathcal{E}^+$ to be given in terms of dipoles graphs at least one of which will feature a number of gluons smaller than two (the "missing" gluons being self-interacting ones). Furthermore, we shall see that the radiation pattern $\mathcal{E}^-$ can also be expressed in terms of dipole graphs, notwithstanding the lack of formal proof that this is possible. However, before going into that, we observe that by applying the merging-line procedure advocated above to the each of the gluons[25] that appear in the matrix in eq. (B.22) we obtain the following:

$$\begin{array}{cccccc} -1 & \bar{2} & \bar{1} & -2 & \bar{2} & \bar{1} \end{array}$$
$$\left(\begin{array}{cccccc} 0 & +1 & -1 & +1 & -1 & +1 \\ & 0 & +1 & -1 & +1 & -1 \\ & & 0 & +1 & -1 & +1 \\ & & & 0 & +1 & -1 \\ & & & & 0 & +1 \\ & & & & & 0 \end{array}\right) \begin{array}{c} -1 \\ \bar{2} \\ \bar{1} \\ -2 \\ \bar{2} \\ \bar{1} \end{array} \longrightarrow \begin{array}{c} \begin{array}{cccc} -1 & \bar{2} & \bar{1} & -2 \end{array} \\ \left(\begin{array}{cccc} 0 & 0 & 0 & 1 \\ & 2 & 0 & 0 \\ & & 2 & 0 \\ & & & 0 \end{array}\right) \end{array} \begin{array}{c} -1 \\ \bar{2} \\ \bar{1} \\ -2 \end{array} . \tag{B.27}$$

As has been anticipated in the discussion given before, in the matrix on the r.h.s. of eq. (B.27) we see the presence of two elements equal to two on the diagonal, which is equivalent to saying that there are two self-connected gluons. Indeed, it is immediate to see that this matrix is representative of the dipole graph $\Gamma_6$ of figure 2, with the corresponding radiation pattern of eq. (6.23).

We now consider the matrices of eq. (B.11), which we construct as explained before, namely by first treating the colour and anticolour of each gluon as a quark and an antiquark, and by merging the relevant lines afterwards. Thus

$$M\left(\mathcal{E}_1^+\right) = \begin{array}{c} \begin{array}{cccccc} -1 & \bar{2} & \bar{1} & -2 & \bar{2} & \bar{1} \end{array} \\ \left(\begin{array}{cccccc} 0 & 1 & 0 & 0 & 0 & 0 \\ & 0 & 0 & 0 & 0 & 0 \\ & & 0 & 0 & 0 & 1 \\ & & & 0 & 1 & 0 \\ & & & & 0 & 0 \\ & & & & & 0 \end{array}\right) \end{array} \begin{array}{c} -1 \\ \bar{2} \\ \bar{1} \\ -2 \\ \bar{2} \\ \bar{1} \end{array} \longrightarrow \begin{array}{c} \begin{array}{cccc} -1 & \bar{2} & \bar{1} & -2 \end{array} \\ \left(\begin{array}{cccc} 0 & 1 & 0 & 0 \\ & 0 & 0 & 1 \\ & & 2 & 0 \\ & & & 0 \end{array}\right) \end{array} \begin{array}{c} -1 \\ \bar{2} \\ \bar{1} \\ -2 \end{array} , \tag{B.28}$$

[25] That is, row/column number 2 and 3 is merged with row/column number 5 and 6, respectively.

JHEP11(2021)045

$$
M\left(\mathcal{E}_3^+\right) = \begin{array}{c} \begin{matrix} -1 & \bar{2} & \bar{1} & -2 & \bar{2} & \bar{1} \end{matrix} \\ \begin{pmatrix} 0 & 0 & 0 & 1 & 0 & 0 \\ & 0 & 1 & 0 & 0 & 0 \\ & & 0 & 0 & 0 & 0 \\ & & & 0 & 0 & 0 \\ & & & & 0 & 1 \\ & & & & & 0 \end{pmatrix} \end{array} \begin{matrix} -1 \\ \bar{2} \\ \bar{1} \\ -2 \\ \bar{2} \\ \bar{1} \end{matrix} \longrightarrow \begin{array}{c} \begin{matrix} -1 & \bar{2} & \bar{1} & -2 \end{matrix} \\ \begin{pmatrix} 0 & 0 & 0 & 1 \\ & 0 & 2 & 0 \\ & & 0 & 0 \\ & & & 0 \end{pmatrix} \end{array} \begin{matrix} -1 \\ \bar{2} \\ \bar{1} \\ -2 \end{matrix} , \qquad \text{(B.29)}
$$

$$
M\left(\mathcal{E}_5^+\right) = \begin{array}{c} \begin{matrix} -1 & \bar{2} & \bar{1} & -2 & \bar{2} & \bar{1} \end{matrix} \\ \begin{pmatrix} 0 & 0 & 0 & 0 & 0 & 1 \\ & 0 & 0 & 0 & 1 & 0 \\ & & 0 & 1 & 0 & 0 \\ & & & 0 & 0 & 0 \\ & & & & 0 & 0 \\ & & & & & 0 \end{pmatrix} \end{array} \begin{matrix} -1 \\ \bar{2} \\ \bar{1} \\ -2 \\ \bar{2} \\ \bar{1} \end{matrix} \longrightarrow \begin{array}{c} \begin{matrix} -1 & \bar{2} & \bar{1} & -2 \end{matrix} \\ \begin{pmatrix} 0 & 0 & 1 & 0 \\ & 2 & 0 & 0 \\ & & 0 & 1 \\ & & & 0 \end{pmatrix} \end{array} \begin{matrix} -1 \\ \bar{2} \\ \bar{1} \\ -2 \end{matrix} . \qquad \text{(B.30)}
$$

We see that the rightmost matrices in eqs. (B.28), (B.29), and (B.30) correspond to the dipole graphs $\Gamma_4$, $\Gamma_3$, and $\Gamma_5$ of figure 2, respectively, with the first and the third being associated with one self-connected gluon (with label $\bar{1}$ and $\bar{2}$ respectively). This concludes the explicit construction of the r.h.s. of eq. (7.82); as was expected from eq. (B.11), all coefficients $c^+$ that appear in the former equation are equal to one.

We now turn to the radiation pattern $\mathcal{E}^-$. We construct a regular dipole representation, thereby avoiding the use of the matrices in eq. (B.16). Explicitly

$$
M\left(\mathcal{E}_{b_1}^-\right) = \begin{array}{c} \begin{matrix} -1 & \bar{2} & \bar{1} & -2 & \bar{2} & \bar{1} \end{matrix} \\ \begin{pmatrix} 0 & 1 & 0 & 0 & 0 & 0 \\ & 0 & 0 & 0 & 0 & 0 \\ & & 0 & 0 & 1 & 0 \\ & & & 0 & 0 & 1 \\ & & & & 0 & 0 \\ & & & & & 0 \end{pmatrix} \end{array} \begin{matrix} -1 \\ \bar{2} \\ \bar{1} \\ -2 \\ \bar{2} \\ \bar{1} \end{matrix} \longrightarrow \begin{array}{c} \begin{matrix} -1 & \bar{2} & \bar{1} & -2 \end{matrix} \\ \begin{pmatrix} 0 & 1 & 0 & 0 \\ & 0 & 1 & 0 \\ & & 0 & 1 \\ & & & 0 \end{pmatrix} \end{array} \begin{matrix} -1 \\ \bar{2} \\ \bar{1} \\ -2 \end{matrix} , \qquad \text{(B.31)}
$$

$$
M\left(\mathcal{E}_{b_2}^-\right) = \begin{array}{c} \begin{matrix} -1 & \bar{2} & \bar{1} & -2 & \bar{2} & \bar{1} \end{matrix} \\ \begin{pmatrix} 0 & 0 & 0 & 0 & 1 & 0 \\ & 0 & 0 & 0 & 0 & 1 \\ & & 0 & 1 & 0 & 0 \\ & & & 0 & 0 & 0 \\ & & & & 0 & 0 \\ & & & & & 0 \end{pmatrix} \end{array} \begin{matrix} -1 \\ \bar{2} \\ \bar{1} \\ -2 \\ \bar{2} \\ \bar{1} \end{matrix} \longrightarrow \begin{array}{c} \begin{matrix} -1 & \bar{2} & \bar{1} & -2 \end{matrix} \\ \begin{pmatrix} 0 & 1 & 0 & 0 \\ & 0 & 1 & 0 \\ & & 0 & 1 \\ & & & 0 \end{pmatrix} \end{array} \begin{matrix} -1 \\ \bar{2} \\ \bar{1} \\ -2 \end{matrix} , \qquad \text{(B.32)}
$$

JHEP11(2021)045

$$
M\left(\mathcal{E}^-_{b_3}\right) = \begin{array}{c} \begin{array}{cccccc} -1 & \bar{2} & \bar{1} & -2 & \bar{2} & \bar{1} \end{array} \\ \left(\begin{array}{cccccc} 0 & 0 & 1 & 0 & 0 & 0 \\ & 0 & 0 & 1 & 0 & 0 \\ & & 0 & 0 & 0 & 0 \\ & & & 0 & 0 & 0 \\ & & & & 0 & 1 \\ & & & & & 0 \end{array}\right) \begin{array}{c} -1 \\ \bar{2} \\ \bar{1} \\ -2 \\ \bar{2} \\ \bar{1} \end{array} \end{array} \longrightarrow \begin{array}{c} \begin{array}{cccc} -1 & \bar{2} & \bar{1} & -2 \end{array} \\ \left(\begin{array}{cccc} 0 & 0 & 1 & 0 \\ & 0 & 1 & 1 \\ & & 0 & 0 \\ & & & 0 \end{array}\right) \begin{array}{c} -1 \\ \bar{2} \\ \bar{1} \\ -2 \end{array} \end{array}, \tag{B.33}
$$

$$
M\left(\mathcal{E}^-_{b_4}\right) = \begin{array}{c} \begin{array}{cccccc} -1 & \bar{2} & \bar{1} & -2 & \bar{2} & \bar{1} \end{array} \\ \left(\begin{array}{cccccc} 0 & 1 & 0 & 0 & 0 & 0 \\ & 0 & 0 & 0 & 0 & 0 \\ & & 0 & 1 & 0 & 0 \\ & & & 0 & 0 & 0 \\ & & & & 0 & 1 \\ & & & & & 0 \end{array}\right) \begin{array}{c} -1 \\ \bar{2} \\ \bar{1} \\ -2 \\ \bar{2} \\ \bar{1} \end{array} \end{array} \longrightarrow \begin{array}{c} \begin{array}{cccc} -1 & \bar{2} & \bar{1} & -2 \end{array} \\ \left(\begin{array}{cccc} 0 & 1 & 0 & 0 \\ & 0 & 1 & 0 \\ & & 0 & 1 \\ & & & 0 \end{array}\right) \begin{array}{c} -1 \\ \bar{2} \\ \bar{1} \\ -2 \end{array} \end{array}, \tag{B.34}
$$

so that

$$
M\left(\mathcal{E}^-\right) = M\left(\mathcal{E}^-_{b_1}\right) + M\left(\mathcal{E}^-_{b_2}\right) + M\left(\mathcal{E}^-_{b_3}\right) - M\left(\mathcal{E}^-_{b_4}\right) \tag{B.35}
$$

$$
= M\left(\mathcal{E}^-_{b_1}\right) + M\left(\mathcal{E}^-_{b_3}\right)\,, \tag{B.36}
$$

with the matrices on the r.h.s. understood to be those obtained by line-merging.

The present case being derived from that of a 3-quark-line process, we know (see the comment before eq. (B.16)) that the equivalent dipole graphs must include quark-antiquark eikonals. These are the elements of the matrices on the l.h.s. of eqs. (B.31)–(B.33) whose row and column indices have opposite parities. Since these eikonals cannot stem from $\mathcal{E}^-$, they are subtracted by the matrix on the l.h.s. of eq. (B.34), whose elements are all of this kind. We point out that while this implies that the $c^-$ coefficients on the r.h.s. of eq. (7.83) do not have the same sign for the 3-quark-line process, this is not the case after line-merging. As we see from the r.h.s. of eqs. (B.31)–(B.34), such an operation leads to degenerate results, and thus to immediate cancellations and to the final result of eq. (B.36), i.e. to a linear combination of dipole graphs with coefficients all equal to one.

The rightmost matrices in eqs. (B.31) and (B.33) correspond to the dipole graphs $\Gamma_2$ and $\Gamma_1$ of figure 2, respectively. Using these identifications, those relevant to $\mathcal{E}$ and $\mathcal{E}^+$ found after eq. (B.27) and (B.30), as well as eq. (B.9), we arrive at

$$
\mathcal{D}(\Gamma_6) = \mathcal{D}(\Gamma_4) + \mathcal{D}(\Gamma_3) + \mathcal{D}(\Gamma_5) - \Big[\mathcal{D}(\Gamma_1) + \mathcal{D}(\Gamma_2)\Big]\,, \tag{B.37}
$$

which coincides with eq. (7.40). This is a direct manifestation of the non-uniqueness of the choices in eq. (7.88), although of limited practical interest (since the present example is relevant to a single closed flow, rather than to the set of all closed flows that one needs to consider in physical applications).

JHEP11(2021)045

**Open Access.** This article is distributed under the terms of the Creative Commons Attribution License (CC-BY 4.0), which permits any use, distribution and reproduction in any medium, provided the original author(s) and source are credited.
